# Fabrication of a protein microarray by fluorous-fluorous interactions

**DOI:** 10.1038/s41598-017-07571-4

**Published:** 2017-08-01

**Authors:** Ben-Yuan Li, Duane S. Juang, Avijit K. Adak, Kuo-Chu Hwang, Chun-Cheng Lin

**Affiliations:** Department of Chemistry, National Tsing Hua University, Hsinchu, Taiwan

## Abstract

Fluorous-modified surfaces have emerged as a powerful tool for the immobilization of fluorous-tagged biomolecules based on their specificity and the strength of fluorous-fluorous interactions. To fabricate a fluorous-based protein microarray, we designed two strategies for site-specific modification of proteins with a fluorous tag: attaching the fluorous tag to the C-termini of expressed proteins by native chemical ligation (NCL) or to the Fc domain of antibodies through boronic acid (BA)-diol interactions. The perfluoro-tagged proteins could be easily purified by fluorous-functionalized magnetic nanoparticles (MNPs) and immobilized on a fluorous chip with minimal non-specific adsorption. Importantly, proteins immobilized on the solid support through non-covalent fluorous-fluorous interactions were sufficiently stable to withstand continuous washing. We believe that this fluorous-fluorous immobilization strategy will be a highly valuable tool in protein microarray fabrication.

## Introduction

One of the most powerful tools in proteomics is the protein microarray, which utilizes immobilized proteins on a solid support for high-throughput analysis of biochemical properties and biological activities^1^. However, a protein’s activity may be significantly influenced by its orientation on the solid support. Additionally, the non-specific adsorption of proteins from biological samples to the solid support may result in high background noise and consequently interfere with the accurate interpretation and analysis of results^2^. Therefore, fabricating a high-activity protein microarray with suppressed non-specific interactions is the most critical task in this field.

The immobilization of proteins via covalent attachment has been thoroughly studied. However, the most common strategies for protein immobilization rely on random covalent bond formation^3, 4^ (for example, amide-bond formation and reductive amination), which could dramatically reduce the activity of the target protein. Hence, many chemists have developed oriented and orthogonal immobilization methods to help resolve the potential problems of protein activity loss; these methods include Diels-Alder ligation^5^, Staudinger ligation^6^, Cu(I)-catalyzed 1,2,3-triazole formation^7^, thiol-ene ligation^8^, enzymatic post-translational modification^9^, and cyanobenzothiazole (CBT) condensation^10^. In contrast, many oriented immobilization strategies utilize non-covalent interactions^4, 11^. Popular examples include DNA-directed immobilization^12^, avidin-biotin interaction^13^, and affinity immobilization of His-tagged fusion proteins to a metal-chelate support^14, 15^. However, such methods have potential drawbacks like the non-specific adsorption of non-target proteins in complex biological samples to the solid support, and insufficient strength of non-covalent interactions, which make the immobilized proteins susceptible to falling off after prolonged incubation under complex biological conditions (such as serum), or continuous vigorous washing. Nevertheless, to take advantage of the high specificity of noncovalent binding strategies, the immobilization surface must be able to resist the adsorption of other proteins that lack an affinity tag. Usually, during the glass preparation process, the glass surface is coated by poly(ethylene) glycol to minimize protein non-specific adsorption^16^. However, both the quality of the glass surface and also the strength of the affinity ligand can affect the immobilization results. Thus, generating a convenient, non-specific binding-resistant, and easily prepared glass surface for the oriented and noncovalent immobilization of proteins is highly challenging.

Fluorous-based substrates have emerged as an attractive candidate for biomolecule immobilization as they possess the unique characteristic of having specific and strong affinity to molecules containing a fluorous group, yet also being highly resistant to non-specific adsorption from non-fluorous molecules^17–19^. Recently, Pohl and co-workers created a fluorous carbohydrate microarray utilizing highly specific fluorous affinity interactions between fluorous-tagged saccharides and fluorous-modified glass surfaces^20, 21^. Spring *et al*.^22^ and Schreiber *et al*.^23^ also developed fluorous small molecule microarrays for analyzing small molecule-protein interactions. More recently, Willi Bannwarth and co-workers^24^ attached a tri-perfluorotag to the enzymes RNase H and rcHRP via random amide bond formation and then immobilized them on fluorous glass surfaces via fluorous-fluorous interactions. These fluorous-based microarrays have the following advantages: (i) high signal-to-noise ratios, (ii) low non-specific binding, (iii) low and uniform background fluorescence, and (iv) simple fabrication workflows.

**Figure 1:**
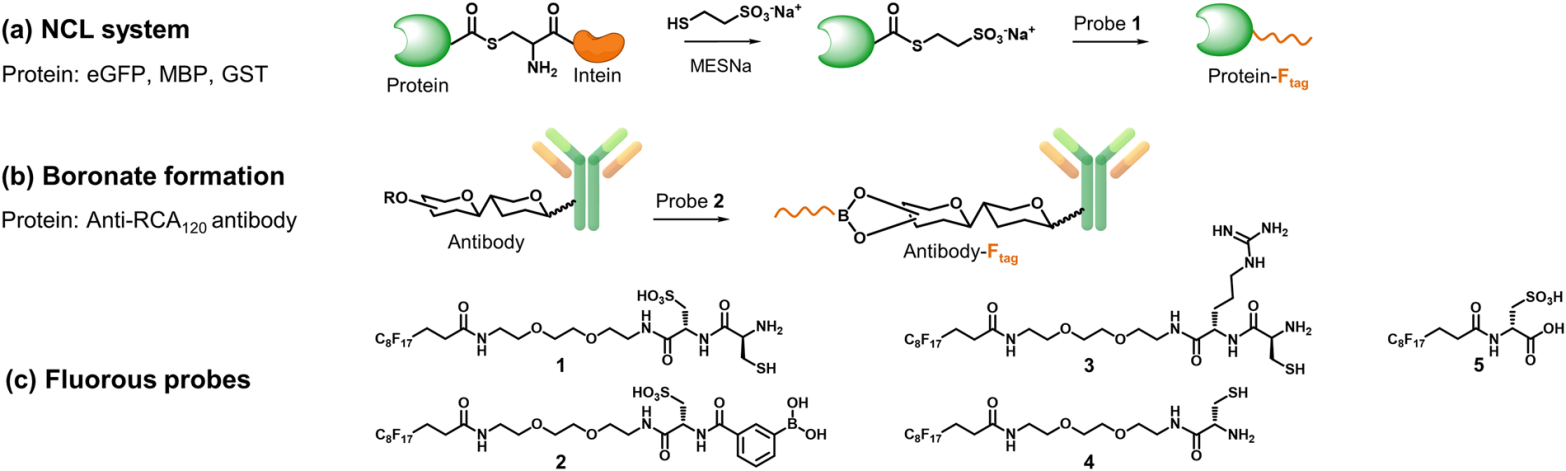
Protein modification strategies. (**a**) Combination of the intein expression system and NCL for the C-terminal modification of enhanced green fluorescent protein (eGFP), maltose binding protein (MBP), and glutathione transferase (GST) with fluorous tags. (**b**) An anti-*Ricinus communis* agglutinin 120 (anti-RCA_120_) antibody was modified with a fluorous tag by forming a boronate ester with a BA-containing fluorous tag. (**c**) Fluorous probes 1 and 2 were used for labeling in this study. Probes 3 and 4 were not used because of their poor solubility in water. Probe 5 was used for the purification of fluorous proteins.

Boronic acids (BAs) are known to rapidly form reversible cyclic boronate esters with cis-diols^25^. Accordingly, BA has been applied as a probe for recognizing various saccharides and glycoproteins in numerous applications^26^. Previously, we reported the use of BA-functionalized glass slides to fabricate oriented protein microarrays by targeting the glycan chain of Fc-fusion proteins^27^. Herein, we developed a straightforward protein microarray fabrication method utilizing a fluorous-based surface for the oriented and noncovalent immobilization of proteins on the surface through fluorous-fluorous interactions. Two types of fluorous probes were designed for the site-specific labeling of proteins with a fluorous tag: one involving the combination of expressed protein through ligation with their N-terminal cysteine residue and the other via boronate ester formation between a BA-probe and an antibody’s glycan moieties (Fig. 1). To make the ligation feasible in buffer solution, ethylene glycol and cysteic acid were incorporated into the fluorous probe to improve its hydrophilicity. Proteins could be easily modified with the fluorous probes by either native chemical ligation (NCL) (for expressed proteins, Fig. 1a) or boronate formation (for antibodies, Fig. 1b) and then immobilized on fluorous surfaces via fluorous interactions with minimal background.

As a proof of concept, eGFP was chosen as a target protein for a model study. eGFP was expressed using the IMPACT system (New England Biolabs)^28^ and was modified by NCL to incorporate a fluorous tag on the protein C-terminal end (F_tag_-protein). To achieve a high ligation yield, the fluorous probe must have sufficient water solubility because the protein labeling yield is positively correlated with the probe’s hydrophilicity.

## Results and Discussion

### Design and synthesis of fluorous tagged probes 1–4

**Figure 2:**
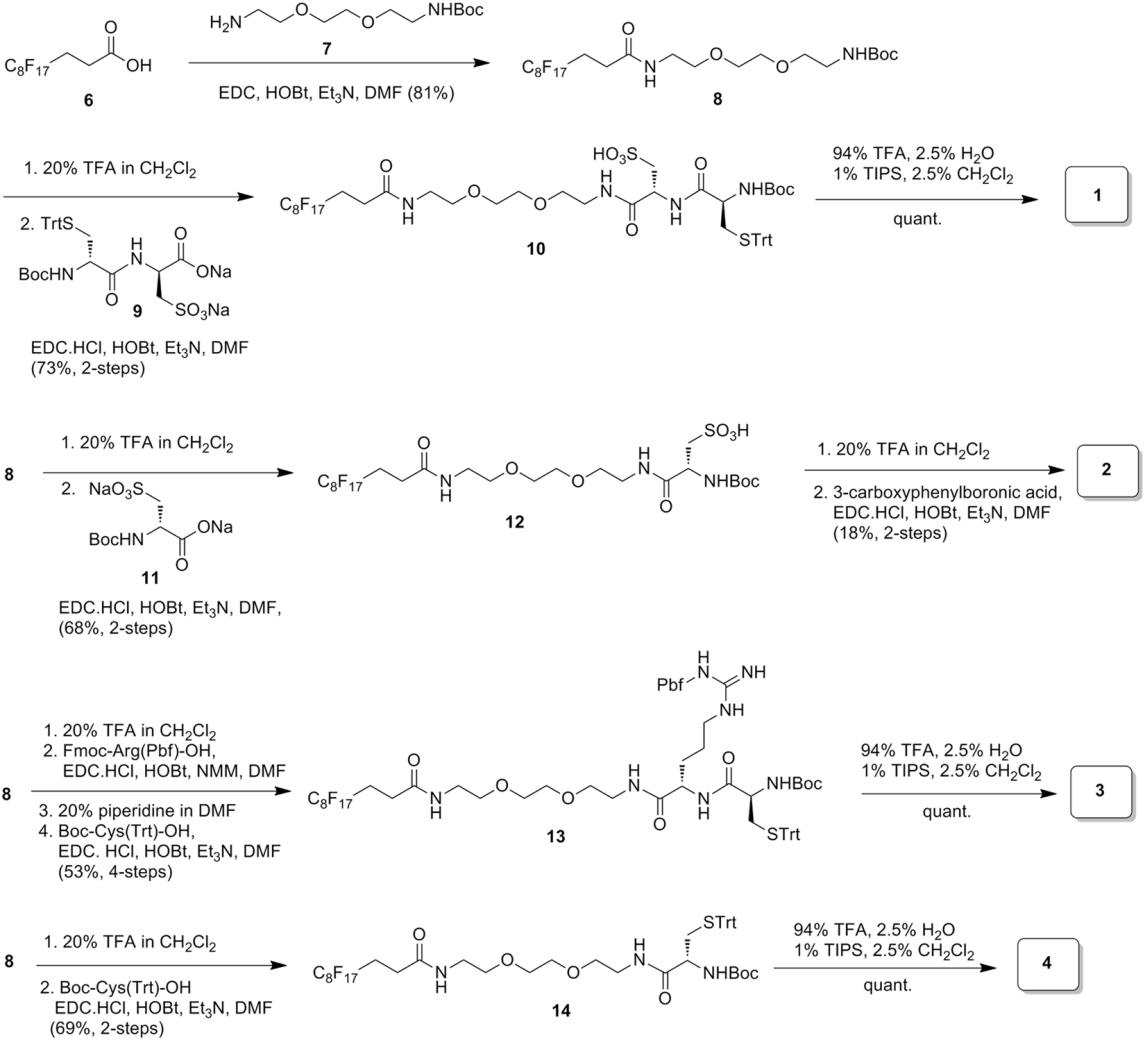
Synthesis of probes **1–4**.

Five probes, as shown in Fig. 1c, were designed and synthesized. In the design of probes **1–4**, we incorporated a short-chain tri(ethylene glycol) linker within the probe, a common reagent generally used for increasing solubility in buffered-aqueous solutions. We also insert polar and charged amino acid residues including cysteic acid to further reduce the hydrophobicity of the fluorous-tagged probes. The preparation of probes **1–4** started from fluorous carboxylic acid **6**, which was first coupled to N-Boc-2,2′-(ethylenedioxy)bis(ethylamine) (**7**) to provide compound **8** in 81% yield (Fig. 2). The Boc-protected derivative **8** with a fluorous tail could serve as a common building block for further functionalization. Thus, removal of the Boc group from **8** by trifluoroacetic acid (TFA) generated an amine, which was reacted with **9** (Supplementary Scheme 1) to give **10** in 73% yield over two-steps. Deprotection of trityl ether was smoothly occurred under acidic conditions (aq. TFA, CH_2_Cl_2_) in the presence of 2.5% triisopropylsilane (TIPS) as a scavenger, which also affected cleavage of the Boc group in **10**, producing probe **1** in essentially quantitative yield. To access BA-containing probe, Boc-protected cysteic acid linker (**11**) was incorporated on amine form of **8** to afford an intermediate **12** (68% over two steps), which was followed by Boc deprotecion and subsequent 1-ethyl-3-(3-dimethylaminorpopyl)carbodiimide hydrochloride (EDC-HCl)/1-hydroxybenzotriazole (HOBT)-mediated coupling with 3-carboxyphenylboronic acid to afford fluorous-tagged BA probe **2** in a modest 18% yield over two-steps. For the synthesis of arginine-containing probe, Fmoc-Arg(Pbf)-OH was first reacted with the amine generated from flourous tag **8**, and extension of the synthesis of Fmoc-protected intermediate was then carried out using 20% piperidine in DMF as the Fmoc deprotecting agent followed by coupling to the carboxylic acid of the incoming amino acid Boc-Cys(Trt)-OH affording **13** (53%, 4-steps). In a similar manner, compound **14** was synthesized in 69% yield starting from **8** and using Boc-Cys(Trt)-OH. Simultaneous cleavage (aq. TFA in CH_2_Cl_2_, 2.5% TIPS) of the trityl ether, Pbf, and Boc protecting groups in **13** and in **14** straightforwardly produced the target probes **3**, and **4**, respectively. The fluorous-tagged small molecule **5** was used for purification of fluorous-modified proteins in this study (see below and, for the synthesis, see Scheme S1).

Herein, we tested multiple combinations of ethylene glycol, with the amino acids arginine and cysteic acid to find an ideal structure for improving the solubility of the fluorous probe (Fig. 1c). In the water-solubility tests (5 mg of compound/1 mL of buffer), only compound **1** completely dissolved in 4-(2-hydroxyethyl)-1-piperazineethanesulfonic acid (HEPES) buffer. At pH 8.0, the sulfonic acid group of cysteic acid acquires a negative charge, which can improve the water solubility of the fluorous probe.

### Protein modification with fluorous tag and fluorous-directed immobilization on fluorous slides

After capturing the expressed fusion protein on a chitin column, 5-mM synthetic fluorous probe **1** was added to the column in the presence of an auxiliary thiol reagent (2-mercaptoethanolsulfonic acid) to allow on-column cleavage and subsequent covalent labeling of the protein with the fluorous probe via NCL (Fig. 1a). The reaction was performed in HEPES buffer (pH 8.0) at 4 °C for 16 h, and the resulting eluent contained F_tag_-protein. To investigate whether the non-covalent F-F interaction is specific or not, a fluorous-modified glass surface was spotted with native eGFP and a mixture of F_tag_-eGFP and native eGFP via a microcontact printer. The results showed that non-covalent affinity was only detected in the presence of a C-terminal fluorous tag (Supplementary Fig. S1), suggesting that the immobilization of proteins using a fluorous tag is indeed highly specific.

### Fluorous tag as a strategy for protein purification using Magnetic nanoparticles (MNPs)

**Figure 3:**
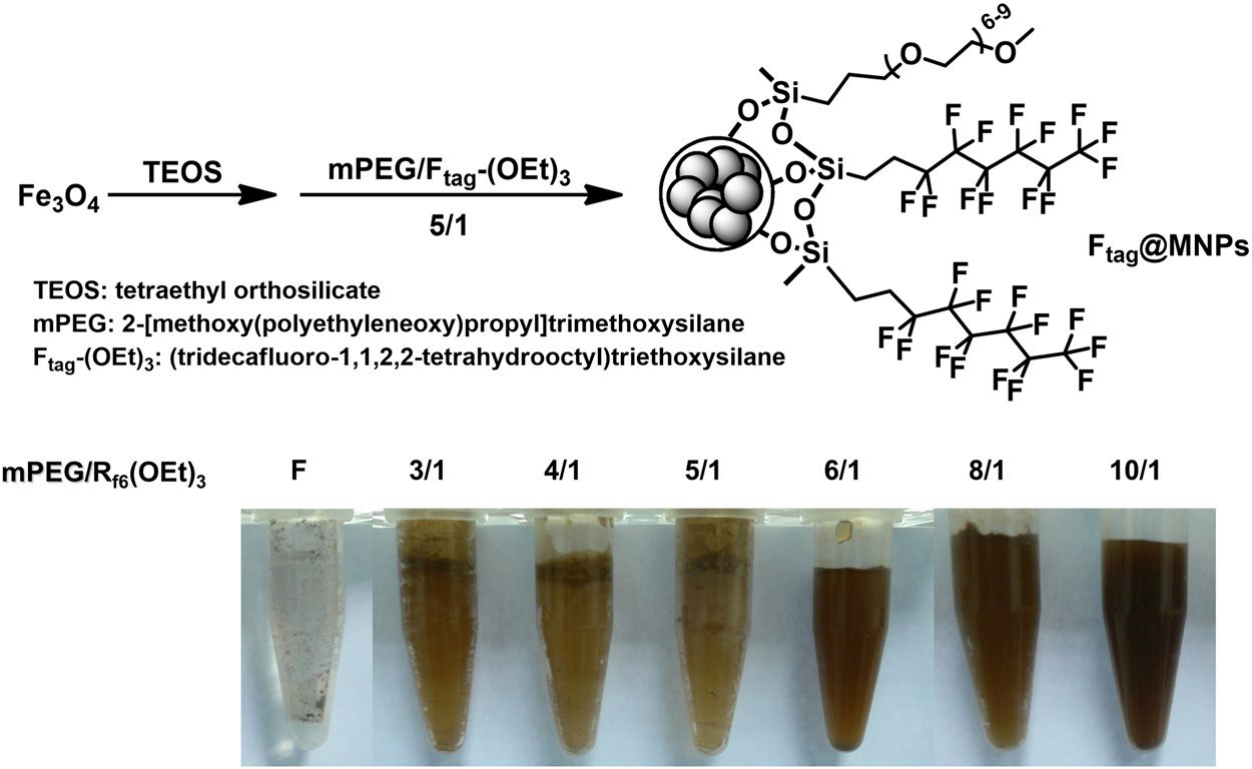
Preparation of the F_tag_@MNPs. The particle surface was functionalized by a sol-gel process using TEOS with mPEG and a fluorous silane. Various ratios of mPEG to fluorous silane were tested (3:1, 4:1, 5:1, 6:1, 8:1, and 10:1) to find an optimal ratio that is soluble in buffer solutions.

The labeling of a protein with a specific functional group is important for studying its interactions with small molecules, DNA, and other proteins. Thus, developing a tool to facilitate the isolation of such modified proteins is imperative because the labeling efficiency is often less than 100%. Magnetic nanoparticles (MNPs) are a well-developed platform for biochemical research^29, 30^ because of their various advantageous properties, such as large surface-area-to-volume ratios, ease of modification, and magnetic behavior. As demonstrated previously^17–24^, non-covalent fluorous-fluorous interactions are highly specific and can effectively reduce the non-specific adsorption of proteins; therefore, fluorous MNPs were proposed as a purification platform for F_tag_-proteins. To make the fluorous functionalized MNPs (F_tag_@MNPs) water compatible, the nanoparticle surface was incorporated with ethylene glycol, which is also able to suppress non-specific protein adsorption. The F_tag_@MNPs were synthesized by modifying our previously reported method^31^. A fluorous functionality was introduced onto the particle surface through a sol-gel process using tetraethyl orthosilicate (TEOS) followed by the addition of 2-[methoxy(polyethyleneoxy)propyl]trimethoxysilane (mPEG) and (tridecafluoro-1,1,2,2-tetrahydrooctyl)triethoxysilane (F_tag_-(OEt)_3_) at various volume ratios—3:1, 4:1, 5:1, 6:1, 8:1, and 10:1—to give F_tag_@MNPs, as illustrated in Fig. 3. The solubility of the resulting F_tag_@MNPs increased as the amount of mPEG increased, and the non-specific adsorption decreased as the amount of (F_tag_-(OEt)_3_) increased (Supplementary Fig. S2). At a mPEG/F_tag_-(OEt)_3_ volume ratio of 6:1, the F_tag_@MNPs become soluble in buffer. It should be noted that the poor solubility of the F_tag_@MNPs can also be improved by the addition of detergent (discussed in the next paragraph). Therefore, to maintain the solubility of the MNPs while simultaneously maximizing the amount of available fluorous tags on the MNPs, F_tag_@MNPs with a mPEG/F_tag_-(OEt)_3_ volume ratio of 5:1 were chosen for use in target protein purification.

**Figure 4:**
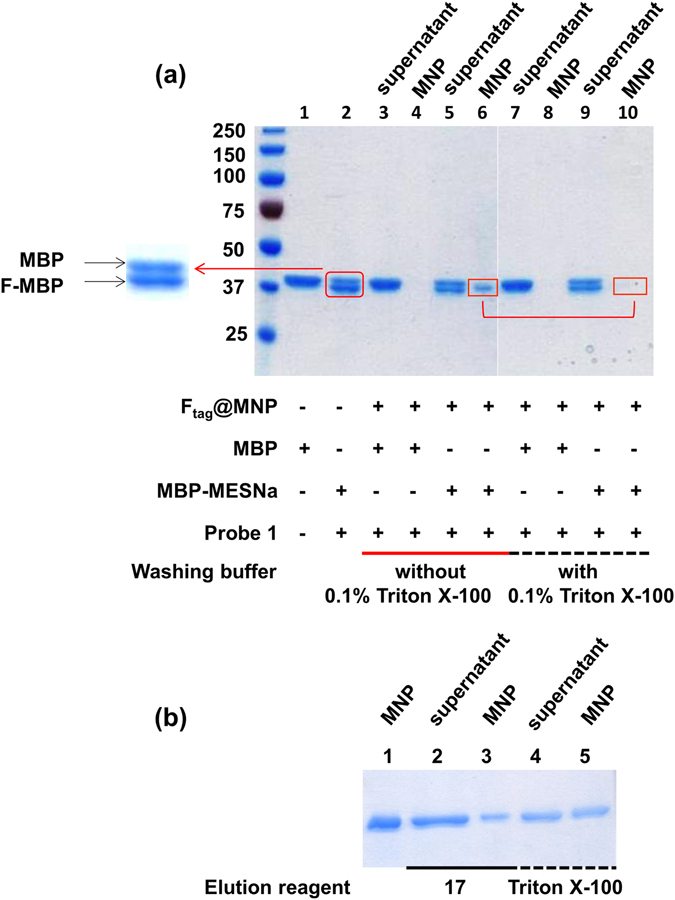
Specific non-covalent interactions between the F_tag_@MNPs and perfluoro-tagged-MBP. (**a**) Lane 1: MBP; lane 2: MBP-MESNa incubated with probe 1; lanes 3, 4, 7 and 8: MBP incubated with probe 1 followed by F_tag_@MNP enrichment; and lanes 5, 6, 9, and 10: MBP-MESNa incubated with probe 1 followed by F_tag_@MNP enrichment. (**b**) Lane 1: MBP-MESNa incubated with probe 1 followed by F_tag_@MNP enrichment and washing with HEPES buffer; lanes 2 and 3: MBP-MESNa incubated with probe 1 followed by F_tag_@MNP enrichment and elution with compound 5; and lanes 4 and 5: MBP-MESNa incubated with probe 1 followed by F_tag_@MNP enrichment and elution with triton X-100. Lanes 3 and 5 were both obtained by eluting MNP only once.

To evaluate the protein purification efficiency achieved using the F_tag_@MNPs, MBP was chosen as a model protein and modified with the fluorous NCL probe (**1**) as described previously. We noted that after concentrating the fluorous tag-modified eGFP to a high concentration, the proteins tended to precipitate, possibly because of fluorous tag self-assembly in the aqueous solution (data not shown). Native polyacrylamide gel electrophoresis (PAGE) analysis was used to investigate the purification results (Fig. 4). As expected, proteins containing the fluoro-tag (F_tag_-MBP) were captured by the F_tag_@MNPs (Fig. 4a, lane 4 vs. lane 6). Interestingly, the presence of 0.1% triton X-100 detergent in the washing buffer inhibited the fluorous-fluorous interactions, resulting in the removal of F_tag_-MBPs from the F_tag_-MNPs (lane 10). Encouraged by these results, we designed two strategies for eluting F_tag_-MBP from F_tag_@MNPs by the addition of 1-mM (or 0.65%) compound **5** (to give F_tag_-MBP_F_) or 0.1% triton X-100 (to give F_tag_-MBP_triton_) in the elution buffer (Fig. 4b, lanes 2 and 4). It should be noted that elution using compound **5** yielded better results than that using triton X-100 (Fig. 4b, lane 3 vs. lane 5), although multiple elutions were required to completely remove the F_tag_-MBPs from the MNPs. To further confirm that the eluted protein was indeed fluoro-tagged, the resulting eluent was analyzed by matrix-assisted laser desorption/ionization time-of-flight mass spectrometry (MALDI-TOF MS); this analysis gave a molecular weight (MW) of 43971 Da, in agreement with the calculated MW of 43905 Da (Supplementary Fig. S3). We also noted that most of the MBPs were ligated with only one fluorous tag, with a smaller proportion ligated with two fluorous tags (because of the formation of the S-S bond), as revealed by the MALDI-TOF MS analysis. Additionally, 1 mg of F_tag_@MNPs can capture an estimated 3.1 μg of F_tag_-protein from solution (Supplementary Fig. S4) with quantitative recovery.

### Specificity of fluorous-fluorous interactions as a strategy for fabrication of fluorous protein microarrays

**Figure 5:**
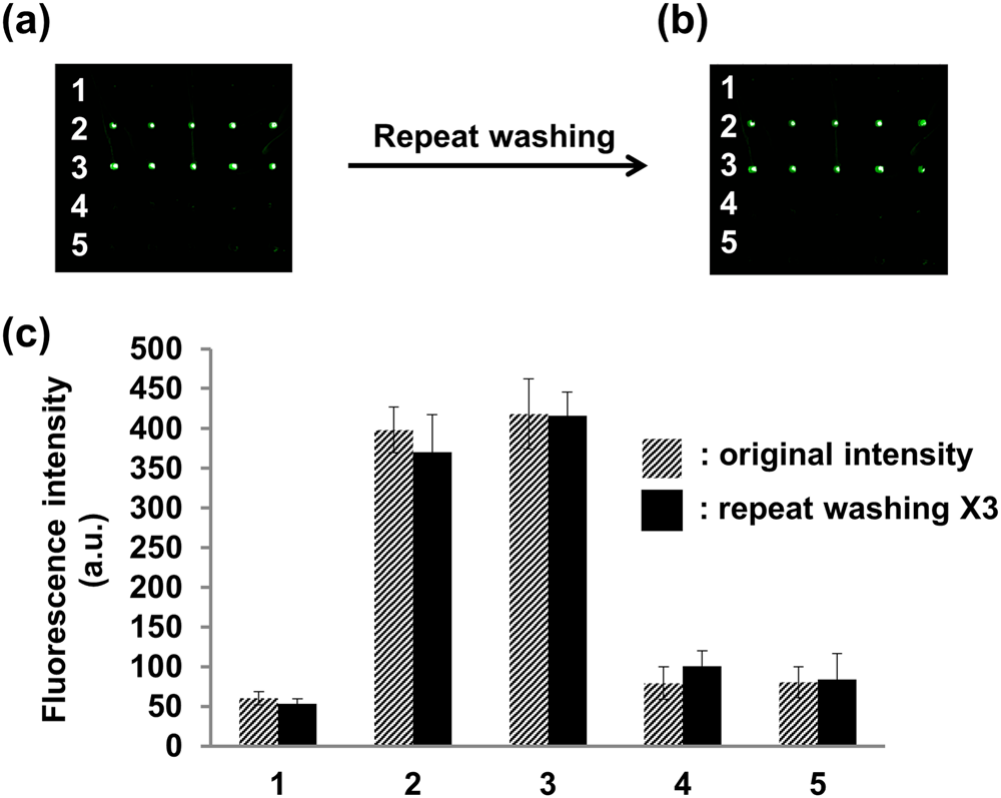
The fluorescence intensities of MBP (row 1), MBP_F_ (row 2), a mixture of F_tag_-MBP_F_ and MBP (row 3), F_tag_-MBP_triton_ (row 4), and a mixture of F_tag_-MBP_triton_ and MBP (row 5) (**a**) before and (**b**) after washing three times. (**c**) The quantified fluorescence intensities of (**a**,**b**).

To investigate non-specific adsorption of the fluorous lipids on the slide surface, MBP (Fig. 5, row 1), F_tag_-MBP_F_ (purified by compound **5**, Fig. 5, row 2), F_tag_-MBP_triton_ (purified by triton X-100, Fig. 5, row 4), and a mixture of native MBP with either F_tag_-MBP_F_ (Fig. 5, row 3) or F_tag_-MBP_triton_ (Fig. 5, row 5) were printed on a fluorous slide. The presence and activity of immobilized MBP were visualized by staining with biotinylated anti-MBP antibody followed by the addition of streptavidin-Cy3. The fluorescence images reveal that only protein with the fluorous tag displayed an emission signal (Fig. 5, rows 2 and 3), while F_tag_-MBP_triton_ exhibited very weak emission. These results indicate that the non-covalent fluorous-fluorous interactions are specific. The weak interactions between F_tag_-MBP_triton_ and the fluorous slide may be attributable to triton X-100 contamination in the protein solution (Fig. 5, rows 4 and 5). Notably, we encountered difficulty in removing triton X-100 by PD MidiTrap G-25 centrifugation, whereas compound **5** was easily removed. Furthermore, the fluorescent signal obtained from a mixture of F_tag_-MBP_F_ and MBP is similar to that of purified F_tag_-MBP_F_, indicating that the presence of non-fluorous modified proteins did not affect the fluorous-fluorous interactions (Fig. 5, rows 2 and 3). Indeed, the fluorescence interactions remained stable even after multiple washes followed by fluorescent staining (Fig. 5b) or incubation under complex biological sample conditions (10% fetal bovine serum [FBS]) for 1 h (Supplementary Fig. S5), showing that the affinity of the fluorous-tagged proteins for the fluorous surface is strong and irreversible, as previously suggested^32^.

To find the suitable concentration of perfluoro-tagged-protein for the reliable fabrication of protein microarrays, various protein sample concentrations were spotted on slides for binding assays. We found that as little as 0.05-μg/μL perfluoro-tagged-MBP was sufficient to obtain a detectible signal and that a concentration of 0.1 μg/μL resulted in a saturated signal (Supplementary Fig. S6). We further estimated that the binding affinity (Kd) of F_tag_-MBP to the fluorous slide surface is 1.04 × 10^−6^ M, although the affinity is likely highly protein dependent (Supplementary Fig. S7). To demonstrate generality of the developed method for multiplex protein microarray fabrication, MBP, GST and the anti-RCA_120_ antibody were chosen as target proteins to be modified with a fluorous tag using probe **1** through NCL at the protein C-terminus or probe **2** through boronate formation at the carbohydrate moiety. The presence of F_tag_-protein on the slide was visualized using RCA_120_ followed by the corresponding biotinylated antibodies and streptavidin-Cy3.

**Figure 6:**
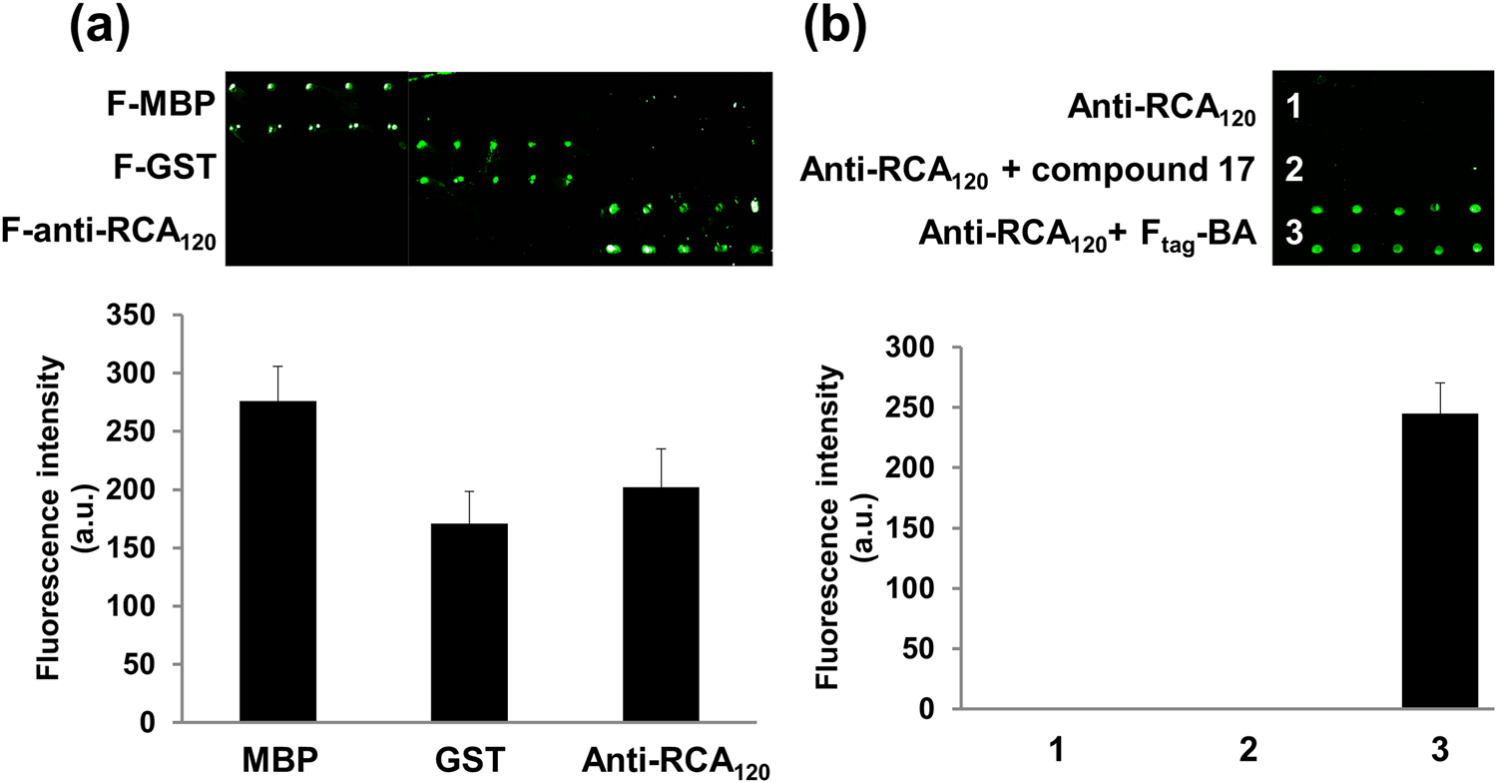
Fluorous protein microarray for the simultaneous detection of multiple proteins. (**a**) MBP, GST, and anti-RCA_120_ were printed on a fluorous slide. (**b**) Confirmation of the specific fluorous-fluorous interactions. Anti-RCA_120_, anti-RCA_120_ pre-incubated with F_tag_-acid (compound 5), and anti-RCA_120_ pre-incubated with F_tag_-BA (compound 2) solutions were spotted on a fluorous slide.

As shown in Fig. 6a, each protein produced its respective fluorescent signals, and no noticeable cross interactions were observed. We noted that there were a few inconsistencies in spot size and shape from batch to batch, probably due to inhomogeneity in the commercially purchased fluorous slides. To further prove that the signal from the anti-RCA_120_ antibody was attributable to site-specific boronate formation through BA rather than non-specific interactions involving the fluorous tag, solutions of anti-RCA_120_, anti-RCA_120_ pre-incubated with F_tag_-acid (compound **5**) and anti-RCA_120_ pre-incubated with F_tag_-BA (compound **2**) and anti-RCA_120_ were spotted on a fluorous slide. The fluorescence image-based binding results (Fig. 6b) show that only the antibody with F_tag_-BA gave a signal, indicating that the F_tag_ was successfully assembled on the antibody by boronate formation.

## Discussion

In summary, we demonstrated the use of a specific non-covalent fluorous-fluorous interaction to fabricate a stable protein microarray. The perfluoro tag can be easily attached to the C-termini of target proteins by NCL and the Fc domains of antibodies by boronate formation. Although the fluorous-fluorous interactions are non-covalent, we demonstrated that they are sufficiently stable to withstand vigorous washing processes and complex biological samples. Importantly, the fluorous surfaces of the slides resist non-specific adsorption, resulting in better detection sensitivity. Previously, the majority of approaches for enhancing the sensitivity of protein microarrays utilize methods that attempt to enhance the assay signal itself. Some examples include photo-initiated polymerization^33^, plasmonic substrates^34^, and silicon nanowires^35^. However in many scenarios, the main bottleneck in assay sensitivity is not due to a lack of signal strength, but becuase of the inability to discern the true signal from the false background due to non-specific binding. As a result, the limit of detection, usually defined as three times the standard deviation of the background signal, is often much higher in such systems when used under complex biological sample conditions. This study sheds light on an alternative approach in enhancing protein microarray sensitivity, in which we do not attempt to enhance the strength of the assay signal itself, but rather try to reduce the non-spcicific background signal. Thus, we believe that this fluorous-fluorous interaction strategy will be a valuable tool for facilitating the development of high-throughput protein microarray screening platforms.

## Methods

### General experimental methods

See the Supporting Information for details of general experimental methods.

### Synthesis of fluorous-tagged probe 1

Compound **10** (100 mg, 0.01 mmol) was dissolved in a mixed solution of TFA/CH_2_Cl_2_/triisopropylsilane (TIPS)/H_2_O (94/2.5/2.5/1 v/v/v/v, 4 mL). The mixture was stirred at room temperature for 30 minutes to deprotect the triphenylmethyl (Trt) and Boc groups. The solution was concentrated under reduced pressure. The resulting residue was washed by hexane (Hex) and EA to give probe **1**, which was used in the next reaction without further purification (97% yield, 70.9 mg). R_*f*_ = 0.4 (1:3 MeOH-DCM) ^1^H NMR (400 MHz, MeOD) δ 4.85-4.78 (m, 1 H), 4.11-4.03 (m, 1 H), 3.63-3.60 (m, 4 H), 3.59-3.53 (m, 4 H), 3.47-3.35 (m, 4 H), 3.30-3.24 (m, 1 H), 3.15 (dd, *J* = 9.6, 14,4 Hz, 1 H), 3.05 (dd, *J* = 6.0, 14,4 Hz, 2 H), 2.61-2.45 (m, 4 H); ^13^C NMR (100 MHz, MeOD) δ 173.0 (x2), 172.1, 168.3, 71.3 (x2), 70.5, 70.3, 56.1, 52.7, 52.5, 40.6, 40.5, 31.1, 27.8 (t, *J* = 22.0 Hz), 27.5, 26.1; ^19^F NMR (376 MHz, CF_3_COOH) δ −81.8 (t, *J* = 11.3 Hz, 3 F), −115.0–−115.3 (m, 2 F), −122.0–−122.2 (m, 2 F), −122.2–−122.5 (m, 4 F), −123.1–−123.4 (m, 2 F), −123.8–−124.1 (m, 2 F), −126.6–−126.9 (m, 2 F); HRMS (ESI) m/z Calcd. for C_23_H_28_F_17_N_4_O_8_S_2_^−^ [M-H]^−^: 875.1077 found 875.1074.

### Synthesis of fluorous-tagged BA probe 2

TFA (1 mL) was added to a solution of compound **12** (430 mg, 0.5 mmol) in DCM (4 mL) in an ice bath. The reaction was stirred at room temperature for 2 h, and then, the solvent was removed under reduced pressure. A solution of the above residue, 3-carboxyphenylboronic acid (166.0 mg, 1.0 mmol), EDC (187.9 mg, 1.0 mmol), HOBt (132.4 mg, 1.0 mmol), and Et_3_N (170.5 μL, 1.2 mmol) in anhydrous DMF (5 mL) was stirred at room temperature for 12 h. The solvent was removed under vacuum. The crude product was purified by silica gel chromatography (30% MeOH in 1:1 EA-Hex) to give probe **2** in 18% yield over two steps (82.9 mg). R_*f*_ = 0.23 (20% MeOH in 1:1 EA-Hex); ^1^H NMR (400 MHz, DMSO-d_6_) δ 9.7 (s, 1 H), 8.61 (d, *J* = 2.4 Hz, 1 H), 8.15 (t, *J* = 5.3 Hz, 1 H), 7.9 (t, *J* = 5.3 Hz, 1 H), 7.28-7.19 (m, 3 H), 6.91 (dt, *J* = 2.2, 6.7 Hz, 1 H), 4.42-4.36 (m, 1 H), 3.5 (s, 4 H), 3.42-3.36 (m, 4 H), 3.24-3.12 (m, 4 H), 2.91-2.80 (m, 2 H), 2.48-2.37 (m, 4 H); ^13^C NMR (100 MHz, DMSO-d_6_) δ 170.7, 169.4, 165.9, 157.3, 135.5, 129.3, 118.2, 117.6, 114.1, 69.5 (x2), 69.0, 68.8, 51.9, 51.3, 38.8, 38.6, 25.9 (t, *J* = 21.9 Hz), 25.7; ^19^F NMR (376 MHz, CF_3_COOH) δ −81.9 (t, *J* = 11.3 Hz, 3 F), −115.0–−115.3 (m, 2 F), −122.0–−122.2 (m, 2 F), −122.2–−122.5 (m, 4 F), −123.1–−123.4 (m, 2 F), −123.8–−124.1 (m, 2 F), −126.6–−126.9 (m, 2 F); HRMS (ESI) m/z Calcd. for C_27_H_29_BF_17_N_3_O_10_S[M-H]^−^: 920.1322 found 920.1323.

### Synthesis of probe 3

Compound **13** (100 mg, 0.1 mmole) was dissolved in a mixed solution containing TFA/CH_2_Cl_2_/TIS/H_2_O (94/2.5/2.5/1 v/v/v/v, 4 mL). The mixture was stirred at room temperature for 30 minutes to deprotect the Trt and Boc groups. The solution was concentrated under reduced pressure. The resulting residue was washed with Hex and EA to give probe **3**, which was used in the next reaction without further purification (95% yield, 56.9 mg). R_*f*_ = 0.5 (2:2:1 EA-MeOH-H_2_O) ^1^H NMR (400 MHz, MeOD) δ 4.35 (dd, *J* = 5.0, 8.6 Hz, 1 H), 4.22 (t, *J* = 5.8 Hz, 1 H), 3.64-3.60 (m, 4 H), 3.58-3.53 (m, 4 H), 3.44-3.36 (m, 4 H), 3.25-3.17 (m, 2 H), 3.10-3.05 (m, 2 H), 2.58-2.48 (m, 4 H), 1.92-1.79 (m, 2 H), 1.79-1.64 (m, 2 H); ^13^C NMR (100 MHz, MeOD) δ 173.7, 173.0, 168.8, 158.7, 71.3, 71.3, 70.5, 70.4, 55.8, 55.0, 42.0, 40.5, 40.4, 30.1, 27.8 (t, *J* = 22.0 Hz), 27.5, 26.5, 26.1; ^19^F NMR (376 MHz, C_6_F_6_) δ −81.9 (t, *J* = 9.4 Hz, 3 F), −115.1–−115.4 (m, 2 F), −122.1–−122.3 (m, 2 F), −122.3–−122.6 (m, 4 F), −123.1–−123.4 (m, 2 F), −123.9–−124.2 (m, 2 F), −126.7–−126.9 (m, 2 F); HRMS (ESI) m/z Calcd. for C_26_H_36_F_17_N_7_O_5_S [M + H]^+^: 882.2305 found 882.2301.

### Synthesis of probe 4

Compound **14** (100 mg, 0.1 mmol) was dissolved in a mixed solution containing TFA/CH_2_Cl_2_/TIS/H_2_O (94/2.5/2.5/1 v/v/v/v, 4 mL). The mixture was stirred at room temperature for 30 minutes to deprotect the Trt and Boc groups. The solution was concentrated under reduced pressure. The resulting residue was washed with Hex to give probe **4**, which was used in the next reaction without further purification (96% yield, 65.7 mg). R_*f*_ = 0.18 (1:9 MeOH-DCM); ^1^H NMR (400 MHz, MeOD) δ 4.01 (t, *J* = 5.8 Hz, 1 H), 3.64-3.61 (m, 4 H), 3.61-3.51 (m, 4 H), 3.48-3.33 (m, 4 H), 3.03 (dd, *J* = 4.8, 14.4 Hz, 1 H), 2.97 (dd, *J* = 6.2, 14.4 Hz, 1 H), 2.61-2.42 (m, 4 H); ^13^C NMR (100 MHz, MeOD) δ 172.9, 168.5, 71.3, 71.2, 70.5, 70.3, 56.1, 40.5, 40.5, 27.8 (t, *J* = 22.0 Hz), 27.5, 26.3; ^19^F NMR (376 MHz, CF_3_COOH) δ −81.9 (t, *J* = 11.3 Hz, 3 F), −115.1–−115.4 (m, 2 F), −122.0–−122.3 (m, 2 F), −122.3–−122.6 (m, 4 F), −123.1–−123.4 (m, 2 F), −123.9–−124.2 (m, 2 F), −126.7–−126.9 (m, 2 F); HRMS (ESI) m/z Calcd. for C_20_H_25_F_17_N_3_O_4_S [M + H]^+^: 726.1294 found 726.1299.

### General method for fluorous-tagged protein spotting

The perfluoro-tagged protein solution was prepared in a printing buffer (20-mM HEPES, 500-mM NaCl and 0.1-mM EDTA with a final glycerol concentration of 10%) and dispensed using a robotic contact arrayer (AD1500 Arrayer, BioDot) fitted with Stealth Pins SMP3 (Arrayit Corp.) onto a fluorous-coated slide. The printing process was performed at 90% relative humidity, and the temperature was maintained below 26 °C. The printed slide was kept in a humidified chamber and maintained at 4 °C for 12 h. Before incubation, the slide was left at room temperature for an additional 2 h to enhance the fluorous-fluorous interactions. The slides were then blocked twice with 1% bovine serum albumin (BSA) solution in phosphate-buffered saline (PBS) for 5 min each and then washed twice with deionized water for 5 min each.

### General method for protein microarray image aquisition and data analysis

The functional protein microarray activities were characterized by using either a biotinylated antigen or a pair of antigens and the corresponding biotinylated secondary antibody and streptavidin-Cy3 in a sandwich detection format (see Supporting Information for details). All images were acquired using a VIDAR Revolution^®^ 4550 scanner with a Cy3 filter. The activity of fluorescent eGFP on the slide was directly measured with a NovaRay Microarray Scanner using a fluorescein isothiocyanate (FITC) filter. The fluorescence intensity of the spots was quantified using the *ArrayVision* software package (version 8.0) with correction for local background. The mean intensity of each spot was taken as a single data point analysis. Spot size is approx. 110 µm in diameter with a spot-to-spot variation (CV) of 10.1%.

## Electronic supplementary material

Supporting information
