# Supplementary material for: Fabrication of a protein microarray by fluorous-fluorous interactions
Source: Sci Rep. 2017 Aug 1;7:7053. doi: 10.1038/s41598-017-07571-4 (PMC5539298; doi:10.1038/s41598-017-07571-4)
Supplement: Supplementary file 1 — Supporting information [file 41598_2017_7571_MOESM1_ESM.pdf]

Supplementary information

Fabrication of a protein microarray by fluorous-fluorous interactions

Ben-Yuan Li, Duane S. Juang, Avijit K. Adak, Kuo-Chu Hwang, and Chun-Cheng Lin*

Department of Chemistry, National Tsing Hua University, Hsinchu, Taiwan

Corresponding author's E-mail: *cclin66@mx.nthu.edu.tw; Tel: +886 3 5753147; Fax: +886 3 5711082*

Contents:

A. Experimental Procedures **S2**

1. General materials and methods, synthesis of fluorous probes **1**, **2**, and compound **5** and characterization details (**Scheme S1**) **S2**
2. Fabrication of fluorous-functionalized superparamagnetic nanoparticles (F_{tag}@MNPs) **S12**
3. Protein overexpression, purification, and modification **(Table S1)** **S13**
4. Immobilization of perfluoro-tag protein on glass slides by fluorous-fluorous interaction **S16**

B. Supplementary Figures **S18**

C. Spectrum **S21**

A. Experimental Procedures

1. General materials and methods.

All buffers and solutions were prepared using deionized Millipore water. All chemicals were purchased from Sigma-aldrich, ACROS or Alfa in the highest purity available unless otherwise mentioned and used without further purification. All solvents were dried and distilled using standard techniques. All reactions were carried out in oven-dried glassware and performed under anhydrous conditions with N_2 gas unless otherwise indicated. The reactions were monitored by analytical thin layer chromatography (TLC) on Merck Silica Gel 60 F_{254}. Detection was accomplished by examination under UV light (254 or 365 nm) and/or by staining with ninhydrin, cerium molybdate or potassium permanganate staining solution.

Silica gel column chromatography was performed using a forced flow of the indicated solvent on silica gel (E. Merck). Fluorous phase chromatography was performed using fluorous solid-phase extraction cartridges containing silica gel bonded with perfluorooctylethylsilyl chains (Fluorous Technologies, Inc.). Size exclusion column chromatography was performed by gravity on polymethacrylic polymer beads (Topopearl HW-40F) with MeOH.

^{1}H and ^{13}C NMR spectra were recorded by Bruker AV-400, AV-600 or Varian MR-400 and were referenced to the solvent used ($CDCl_3$, δ 7.24 and 77 ppm; CD_3OD, δ 3.31 and 49 ppm; DMSO-d_6, δ 2.5 and 39.5 ppm for ^{1}H and ^{13}C, respectively). ^{19}F NMR spectra was recorded in hexafluorobenzene (δ -164.9 ppm) or trifluoroacetic acid δ -76.5 ppm) by Varian MR-400. Multiplicities are reported by using the following abbreviations: s = singlet, d = doublet, t = triplet, q = quartet, m = multiplet, br = broad; *J* = coupling constant values are expressed in Hz. High-resolution mass spectra were recorded under ESI mass spectroscopy conditions.

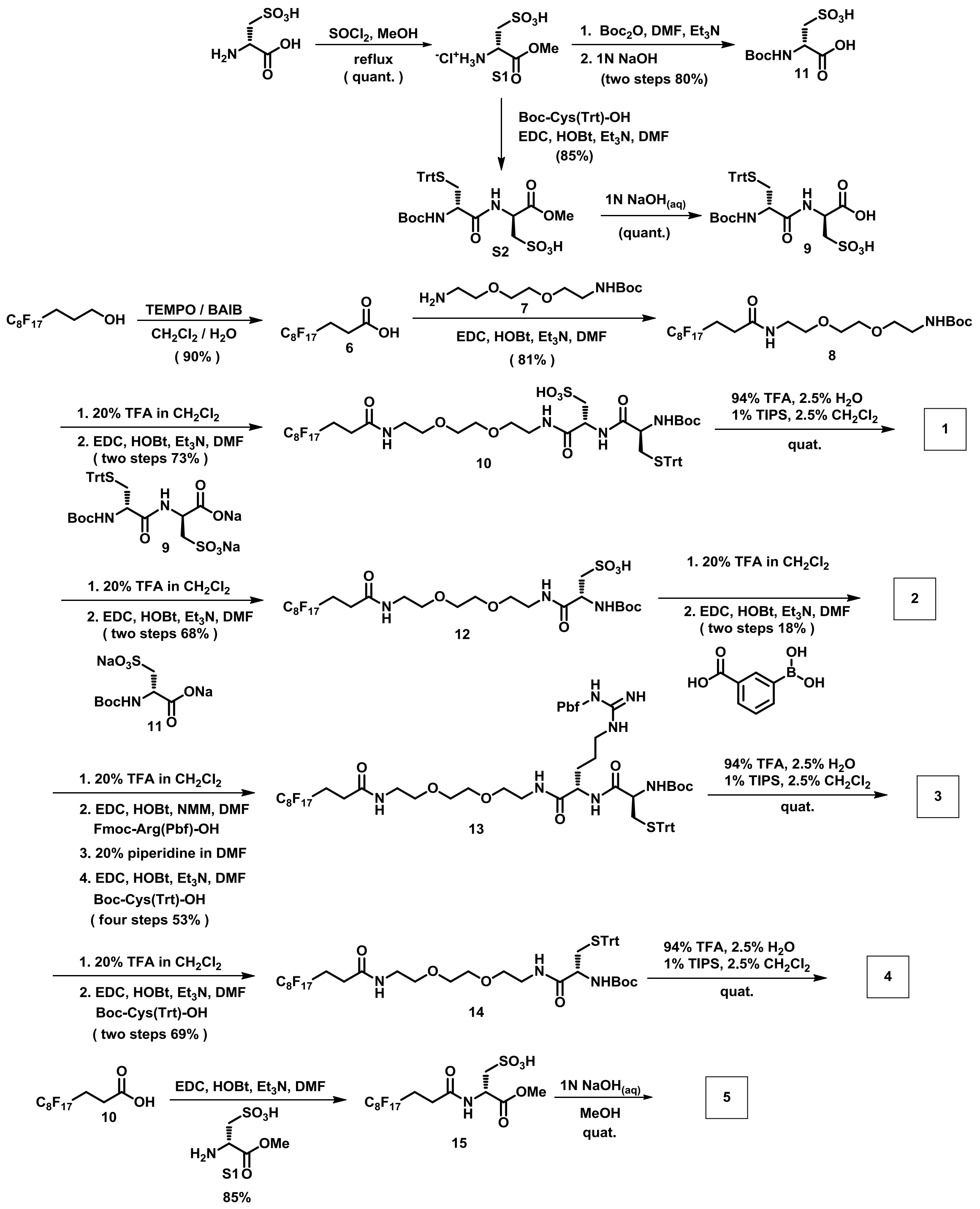

Scheme S1. Syntheses of fluorous probes **1**, **2**, **3**, **4**, and compound **5**.

Compound 5. Compound **15** (660.0 mg, 1.0 mmol) was dissolved in 1-N $NaOH_{(aq)}$ (3 mL, 3 mmol) in an ice bath. The reaction was stirred at room temperature for 3 h. The resulting solution was neutralized with $HCl_{(aq)}$, and the solvent was removed under reduced pressure. The resulting residue was purified by Toyopearl HW-40F size-exclusion chromatography to afford compound **5** as a white solid in 81% yield (520.8 mg). R_f = 0.6 (6:2:1 PrOH-H_2O-AcOH); ^1H NMR (400 MHz, MeOD) δ 4.65 (dd, *J* = 3.0, 9.8 Hz, 1H), 3.39 (dd, *J* = 3.2, 14.4 Hz, 1H), 3.18 (dt, *J* = 10.0, 174.4 Hz, 1H), 2.68 – 2.44 (m, 4H); ^{13}C NMR (100 MHz, MeOD) δ 176.5, 172.3, 53.4, 53.3, 27.9 (t, *J* = 22.0 Hz), 27.7; ^{19}F NMR (376 MHz, CF_3COOH) δ -81.9 (t, *J* = 9.4 Hz, 3F), -115.0 – -115.3 (m, 2F), -122.0 – -122.2 (m, 2F), -122.2 – -122.5 (m, 4F), -123.1 – -123.4 (m, 2F), -123.8 – -124.1 (m, 2F), -126.6 – -126.9 (m, 2F); HRMS (ESI) m/z calculated for $C_{14}H_9F_{17}NO_6S^-$ $[M-H]^-$: 641.9879 found 641.9882.

Compound S1. Cysteic acid monohydrate (2.0 g, 10.7 mmol) was dissolved in anhydrous methanol (50 mL), and the mixture was cooled to 0 °C. Thionyl chloride (2.3 mL, 32.1 mmol) was added dropwise at 0 °C, and the reaction mixture was stirred for 3 h at 50 °C. The reaction solvent and thionyl chloride were removed by evaporation under reduced pressure. The residue was washed with cold acetone to give the desired product **S1** as a white solid in 99% yield (2.3 g), and this product was used in the next step without further purification. R_f =0.75 (6:5:1:1 EA-MeOH-H_2O-AcOH) ^1H NMR (400 MHz, DMSO-d_6) δ 8.26 (s, 3H), 4.22 (dd, *J* = 3.4, 8.2 Hz, 1H), 3.72 (s, 3H), 3.0 (dd, *J* = 3.6, 14.4 Hz, 1H), 2.93 (dd, *J* = 8.2, 14.2 Hz, 1H); ^{13}C NMR (100 MHz, DMSO-d_6) δ 168.4, 53.0, 49.9, 49.1; HRMS (ESI) m/z calculated for $C_4H_9NNaO_5S^+$ $[M+Na]^+$: 206.0099 found 206.0097.

Compound 11. tert-Butyloxycarbonyl $(Boc)_2O$ (3.4 g, 15.6 mmol) at room temperature was added to a solution of compound **S1** (1.9 g, 10.4 mmol) and trimethylamine (3.8 mL, 27.2 mmol) in dimethylformamide (DMF) (50 mL). The reaction mixture was stirred at 50 °C for 4 h. After the starting material was consumed, the solvent was removed under reduced pressure to give a crude product, which was used in the next step without further purification (R_f = 0.25, 1:5 MeOH-dichloromethane [DCM]). The above compound (2.8 g, 10.0 mmol) was dissolved in 1-N NaOH (26.0 mL, 26.0 mmol) in an ice bath. The solution was stirred at 4 °C for 3 h and neutralized by the addition of 1-N aqueous HCl. The solvent was removed under reduced pressure. The resulting residue was purified by P2 size-exclusion chromatography to afford compound **11** as a white solid (2.3 g, 80% yield in two steps). R_f = 0.1 (1:5 MeOH-DCM); ^{1}H NMR (400 MHz, MeOD) δ 4.30 (dd, J = 3.2, 8.8 Hz, 1H), 3.28 (dd, J = 3.6, 14.4 Hz, 1H), 3.13 (dd, J = 14.0, 9.2 Hz, 1H), 1.44 (s, 9H); ^{13}C NMR (100 MHz, MeOD) δ 178.2, 157.7, 80.3, 54.5, 54.0, 28.8 (3C); HRMS (ESI) m/z calculated for $C_8H_{14}NO_7S^-$ $[M-H]^-$: 268.0491 found 268.0487.

Compound S2. A solution of compound **S1** (200 mg, 0.9 mmol), Boc-Cys(Trt)-OH (556.3 mg, 1.2 mmol), 1-ethyl-3-(3-dimethylaminopropyl)carbodiimide (EDC) (287.6 mg, 1.5 mmol), hydroxybenzotriazole (HOBt) (202.7 mg, 1.5 mmol), and Et_3N (348.7 μL, 2.5 mmol) in anhydrous DMF (10.0 mL) was stirred at room temperature for 12 h. The solvent was removed under vacuum. The residue was purified by flash silica gel column chromatography (20% MeOH in 1:1 ethyl acetate-hexane [EA-Hex]) to give the desired product **S2** in 85% yield (489.7 mg). R_f = 0.27 (20% MeOH in 1:1 EA-Hex); ^{1}H NMR (400 MHz, MeOD) δ 7.43-7.35 (m, 6H), 7.31-7.24 (m, 6H), 7.24-7.17 (m, 3H), 4.78 (t, J = 5.6 Hz, 1H), 4.04-3.97 (m, 1H), 3.67 (s, 3H), 3.28-3.24 (m, 2H), 2.62 (dd, J = 5.2, 12.4 Hz,

1H), 2.55 (dd, J = 8.8, 12.4 Hz, 1H), 1.45 (s, 9H); ^{13}C NMR (100 MHz, MeOD) δ 172.8, 171.7, 157.3, 145.9 (x3), 130.7 (x6), 129.0 (x6), 127.9 (x3), 81.0, 67.9, 55.1, 53.1, 52.0, 50.8, 35.3, 28.7 (x3); HRMS (ESI) m/z calculated for $C_{31}H_{35}N_2O_8S_2^-$ [M-H]$^-$: 627.1835 found 627.1830.

Compound 9. Compound **S2** (1.2 g, 1.9 mmol) was dissolved in 1-N $NaOH_{(aq)}$ (5.7 mL, 5.7 mmol) in an ice bath. The mixture was stirred at room temperature for 3 h. The resulting solution was neutralized with $HCl_{(aq)}$, and the solvent was removed under reduced pressure. The resulting residue was purified by reverse-phase silica gel column chromatography to afford compound **S2** as a white solid in 99% yield (1.2 g). R_f = 0.27 (20% MeOH in 1:1 EA-Hex); ^{1}H NMR (400 MHz, MeOD) δ 7.43-7.35 (m, 6H), 7.34-7.24 (m, 6H), 7.24-7.17 (m, 3H), 4.52-4.42 (m, 1H), 4.15-4.06 (m, 1H), 3.31-3.25 (m, 1H), 3.23-3.15 (m, 1H), 2.69 (dd, J = 4.4, 12.4 Hz, 1H), 2.53 (dd, J = 9.4, 12.6 Hz, 1H), 1.46 (s, 9H); ^{13}C NMR (100 MHz, MeOD) δ 176.8, 172.0, 157.7, 146.1 (x3), 130.7 (x6), 128.9 (x6), 127.8 (x3), 80.9, 67.8, 55.2, 53.5 (x2), 35.6, 28.8 (x3); HRMS (ESI) m/z calculated for $C_{30}H_{33}N_2O_8S_2^-$ [M-H]$^-$: 613.1678 found 613.1669.

Compound 6. A solution of 3-(perfluorooctyl)propan-1-ol (2.5 g, 5.2 mmol, Fluorous Technologies, Inc.), (2,2,6,6-tetramethylpiperidin-1-yl)oxyl (TEMPO) (265.6 mg, 1.7 mmol), and (diacetoxyliodo)benzene (4.2 g, 13.0 mmol) in a solution of DCM (20 mL) and H_2O (10 mL) was vigorously stirred at room temperature for 5 h. The resulting product was suspended in FC-72 and washed three times with H_2O and cold DCM to obtain the desired product **6** (2.2 g, 90% yield). R_f = 0.59 (10% MeOH in 1:1 EA-Hex); ^{1}H NMR (400 MHz, DMSO-d$_6$) δ 12.7 (m, 1H), 2.47-2.26 (m, 4H); ^{13}C NMR (100 MHz, DMSO-d$_6$) δ

172.0, 25.6 (t, J = 21.5 Hz), 24.6; ^{19}F NMR (376 MHz, C_6F_6) δ -84.4 (t, J = 9.4 Hz, 3F), -116.9 – -117.1 (m, 2F), -124.3 – -124.6 (m, 2F), -124.6 – -124.9 (m, 4F), -125.6 – -125.8 (m, 2F), -125.9 – -126.1 (m, 2F), -129.2 – -129.4 (m, 2F); HRMS (ESI) m/z calculated for $C_{11}H_3F_{17}O_2$ $[M-H]^-$: 490.9940 found 490.9917.

Compound 8. 2,2'-(Ethylenedioxy)bis(ethylamine) (2.7 g, 18.2 mmol) in DCM (18 mL) was treated with Boc_2O (654.8 mg, 3.0 mmol) in DCM (18 mL) at 0 °C for 1 h and then stirred at room temperature for 12 h. The organic phase was washed with water until all of the unreacted starting material was extracted. The organic layer was dried over anhydrous $MgSO_4$ and then concentrated to dryness to obtain N-Boc-2,2'-(ethylenedioxy)bis(ethylamine) (**7**) quantitative yield. A solution of compound **6**, amine **7** (1.2 g, 2.4 mmol), EDC (701.3 mg, 3.7 mmol), HOBt (494.3 mg, 3.7 mmol), and Et_3N (680.4 μL, 4.9 mmol) in anhydrous DMF (24.0 mL) was stirred for 12 h at room temperature. The solvent was removed under vacuum. The crude product was purified by solid-phase extraction using a fluorous solid-phase extraction cartridge. Non-fluorous compounds were eluted with 80% MeOH/water, and the desired product was eluted with 100% MeOH. The solvent was removed under reduced pressure, and product **8** was obtained as a white solid in 81% yield via two steps (1.4 g). R_f = 0.43 (10% MeOH in 1:1 EA-Hex); ^{1}H NMR (400 MHz, MeOD) δ 3.65-3.58 (m, 4H), 3.56 (t, J = 5.4 Hz, 2H), 3.52 (t, J = 5.6 Hz, 2H), 3.39 (t, J = 5.4 Hz, 2H), 3.23 (t, J = 5.6 Hz, 2H), 2.58-2.42 (m, 4H), 1.43 (s, 9H). ^{13}C NMR (100 MHz, MeOD) δ 172.8, 158.4, 80.0, 71.3 (x2), 71.1, 70.6, 41.2, 40.5, 28.8 (x3), 27.8 (t, J = 21.5 Hz), 27.5; ^{19}F NMR (376 MHz, C_6F_6) δ -82.1 (t, J = 9.4 Hz, 3F), -115.2 – -115.5 (m, 2F), -122.1 – -122.4 (m, 2F), -122.4 – -122.7 (m, 4F), -123.2 – -123.6 (m, 2F), -124.0 – -124.3 (m, 2F), -126.8 – -127.1 (m, 2F); HRMS (ESI) m/z

calculated for $C_{22}H_{27}F_{17}N_2NaO_5^+$ [M+Na]$^+$: 745.1546 found 745.1537.

Compound 10. Trifluoroacetic acid (TFA) (0.8 mL) was added to a solution of compound **8** (300.0 mg, 0.4 mmol) in DCM (3.2 mL) in an ice bath. The reaction was stirred at room temperature for 2 h, and then, the solvent was removed under reduced pressure. A solution of the resulting residue, compound **9** (368.4 mg, 0.6 mmol), EDC (119.5 mg, 0.6 mmol), HOBt (84.2 mg, 0.6 mmol), and Et_3N (144.9 μL, 1.0 mmol) in anhydrous DMF (4 mL) was stirred at room temperature for 12 h. The solvent was removed under vacuum. The crude product was purified by solid-phase extraction using a fluorous solid-phase extraction cartridge. Non-fluorous compounds were eluted with 60% MeOH/water, and the desired product was eluted with 100% MeOH. The solvent was removed under reduced pressure to yield compound **10** as a white solid in 73% yield via two steps (355.7 mg). R_f = 0.3 (20% MeOH in 1:1 EA-Hex); ^{1}H NMR (400 MHz, MeOD) δ 7.42-7.36 (m, 6H), 7.32-7.25 (m, 6H), 7.25-7.18 (m,3H), 4.71-4.64 (m, 1H), 3.93-3.84 (m, 1H), 3.59-3.56 (m, 4H), 3.56-3.48 (m, 4H), 3.42-3.32 (m, 4H), 3.29-3.17 (m, 4H), 2.68-2.44 (m, 6H), 1.45 (s, 9H); ^{13}C NMR (100 MHz, MeOD) δ 173.0 (x2), 172.4, 157.8, 145.9 (x3), 130.7 (x6), 129.1 (x6), 128.0 (x3), 81.3, 71.4, 71.3, 70.6, 70.3, 68.1, 55.7, 52.4, 52.0, 40.6, 40.6, 34.8, 28.8 (x3), 27.7 (t, *J* = 22.0 Hz), 27.5; ^{19}F NMR (376 MHz, C_6F_6) δ -82.0 (t, *J* = 9.4 Hz, 3F), -115.1 – -115.4 (m, 2F), -122.1 – -122.3 (m, 2F), -122.3 – -122.6 (m, 4F), -123.1 – -123.4 (m, 2F), -123.9 – -124.2 (m, 2F), -126.7 – -126.9 (m, 2F); HRMS (ESI) m/z calculated for $C_{47}H_{50}F_{17}N_4O_{10}S_2^-$ [M-H]$^-$: 1217.2697 found 1217.2699.

Compound 12. TFA (0.8 mL) was added to a solution of compound **8** (300 mg, 0.4 mmol) in DCM (3.2 mL) in an ice bath. The mixture was stirred at room temperature for 2 h, and

then, the solvent was removed under reduced pressure. A solution of the above compound, compound **7** (169.8 mg, 0.6 mmol), EDC (119.5 mg, 0.6 mmol), HOBt (84.2 mg, 0.6 mmol), and Et_3N (144.9 μL, 1.0 mmol) in anhydrous DMF (4 mL) was stirred at room temperature for 12 h. The solvent was removed under vacuum. The crude residue was purified by flash silica gel column chromatography (30% MeOH in 1:1 EA-Hex) to give the desired product **12** in 68% yield (237.8 mg). R_f = 0.41 (30% MeOH in 1:1 EA-Hex); ^{1}H NMR (400 MHz, MeOD) δ 4.45-4.36 (m, 1H), 3.62 (s, 4H), 3.58-3.54 (m, 4H), 3.45-3.36 (m, 4H), 3.23-3.13 (m, 2H), 2.62-2.45 (m, 4H), 1.44 (s, 9H); ^{13}C NMR (100 MHz, MeOD) δ 173.6, 172.9, 157.5, 80.9, 71.32 (x2), 70.6, 70.5, 53.7, 52.8, 40.5, 40.5, 28.7 (x3), 27.8 (t, *J* = 22.2 Hz), 27.5; ^{19}F NMR (376 MHz, C_6F_6) δ -81.8 (t, *J* = 11.3 Hz, 3F), -115.0 – -115.3 (m, 2F), -122.0 – -122.2 (m, 2F), -122.2 – -122.5 (m, 4F), -123.1 – -123.4 (m, 2F), -123.8 – -124.1 (m, 2F), -126.6 – -126.9 (m, 2F); HRMS (ESI) m/z calculated for $C_{25}H_{31}F_{17}N_3O_9S^-$ [M-H]$^-$: 872.1510 found 872.1511.

Compound 13. TFA (0.8 mL) was added to a solution of compound **8** (300.0 mg, 0.4 mmol) in DCM (3.2 mL) in an ice bath. The mixture was stirred at room temperature for 2 h, and then, the solvent was removed under reduced pressure. A solution of the above crude compound, Fmoc-Arg(Pbf)-OH (389.3 mg, 0.6 mmol), EDC (119.5 mg, 0.6 mmol), HOBt (84.2 mg, 0.6 mmol), and N-methylmorpholine (NMM) (144.9 μL, 1.0 mmol) in anhydrous DMF (4 mL) was stirred at room temperature for 12 h. The solvent was removed under vacuum. The crude product was purified by solid-phase extraction using a fluorous solid-phase extraction cartridge. Non-fluorous compounds were eluted with 80% MeOH/water, and the desired product was eluted with 100% MeOH. The solvent was removed under reduced pressure to afford the desired compound as a white solid (374.0 mg); R_f = 0.47

(10% MeOH in 1:1 EA-Hex). Piperidine (0.6 mL) was added to a solution of the above compound (374.0 mg, 0.3 mmol) in DMF (2.4 mL). The mixture was stirred at room temperature for 2 h, and then, the solvent was removed under reduced pressure. A solution of the above compound, Boc-Cys(Trt)-OH (231.2 mg, 0.5 mmol), EDC (95.9 mg, 0.5 mmol), HOBt (67.6 mg, 0.5 mmol), and Et_3N (111.6 μL, 0.8 mmol) in anhydrous DMF (3 mL) was stirred for 12 h at room temperature. The solvent was removed under vacuum. The crude product was purified by solid-phase extraction using a fluorous solid-phase extraction cartridge. Non-fluorous compounds were eluted with 80% MeOH/water, and the desired product was eluted with 100% MeOH. The solvent was removed under reduced pressure to give compound **13** as a white solid in 53% yield via four steps (318.7 mg). R_f = 0.5 (10% MeOH in 1:1 EA-Hex); ^{1}H NMR (400 MHz, MeOD) δ 7.41-7.34 (m, 6H), 7.32-7,26 (m, 6H), 7.24-7.18 (m, 3H), 4.38-4.30 (m, 1H), 3.88 (t, *J* = 6.4 Hz, 1H), 3.60-3.53 (m, 4H), 3.51 (t, *J* = 5.6 Hz, 2H), 3.47 (t, *J* = 5.6 Hz, 2H), 3.36 (t, *J* = 5.4 Hz, 2H), 3.24-3.05 (m, 2H), 2.97 (s, 2H), 2.56 (s, 3H), 2.55-2.43 (m, 9H), 2.06 (s, 3H), 1.87-1.73 (m, 1H), 1.67-1.59 (m, 1H), 1.59-1.50 (m, 2H), 1.44 (s, 9H), 1.41 (s, 6H); ^{13}C NMR (100 MHz, MeOD) δ 173.5, 173.4, 172.8, 159.9, 158,1, 157.4, 145.9 (x3), 139.4, 134.4, 133.5, 130.7 (x6), 129.1 (x6), 128.0 (x3), 126.0, 118.4, 87.6, 80.9, 71.3 (x2), 70.5, 70.3, 68.0, 55.2, 54.0, 44.0, 41.2, 40.5 ,40.3, 34.9, 30.4, 28.7 (x5), 27.7 (t, *J* = 21.5 Hz), 27.5, 26.5, 19.6, 18.4, 12.5; ^{19}F NMR (376 MHz, C_6F_6) δ -81.9 (t, *J* = 9.4 Hz, 3F), -115.1 – -115.4 (m, 2F), -122.1 – -122.3 (m, 2F), -122.3 – -122.6 (m, 4F), -123.1 – -123.4 (m, 2F), -123.9 – -124.2 (m, 2F), -126.7 – -126.9 (m, 2F); HRMS (ESI) m/z calculated for $C_{63}H_{75}F_{17}N_7O_{10}S_2^+$ $[M+H]^+$: 1476.4745 found 1476.4741.

Compound 14. TFA (0.8 mL) was added to a solution of compound **8** (300.0 mg, 0.4 mmol) in DCM (3.2 mL) in an ice bath. The reaction was stirred at room temperature for 2 h, and then, the solvent was removed under reduced pressure. A solution of the above crude compound, Boc-Cys(Trt)-OH (288.9 mg, 0.6 mmol), EDC (119.5 mg, 0.6 mmol), HOBt (84.2 mg, 0.6 mmol), and Et_3N (144.9 μL, 1.0 mmol) in anhydrous DMF (4 mL) was stirred at room temperature for 12 h. The solvent was removed under vacuum. The crude product was purified by solid-phase extraction using a fluorous solid-phase extraction cartridge. Non-fluorous compounds were eluted with 80% MeOH/water, and the desired product was eluted with 100% MeOH. The solvent was removed under reduced pressure to afford compound **14** as a white solid in 69% yield via two steps (305.9 mg). R_f = 0.48 (10% MeOH in 1:1 EA-Hex); ^{1}H NMR (400 MHz, MeOD) δ 7.41-7.36 (m, 6H), 7.30–7.24 (m, 6H), 7.23–7.17 (m, 3H), 3.97 (t, *J* = 6.4 Hz, 1H), 3.58–3.54 (m, 4H), 3.53–3.47 (m, 4H), 3.42–3.32 (m, 4H), 2.59-2.42 (m, 6H), 1.42 (s, 9H); ^{13}C NMR (100 MHz, MeOD) δ 173.0, 172.7, 157.2, 146.0 (x3), 130.7 (x6), 129.0 (x6), 127.9 (x3), 80.8, 71.3 (x2), 70.5, 70.5, 67.9, 55.1, 40.5, 40.4, 35.5, 28.7 (x3), 27.7 (t, *J* = 22.0 Hz), 27.4; ^{19}F NMR (376 MHz, C_6F_6) δ -82.1 (t, *J* = 9.4 Hz, 3F), -115.2 – -115.5 (m, 2F), -122.2 – -122.5 (m, 2F), -122.5 – -122.8 (m, 4F), -123.3 – -123.6 (m, 2F), -124.0 – -124.3 (m, 2F), -126.9 – -127.1 (m, 2F); HRMS (ESI) m/z calculated for $C_{44}H_{46}F_{17}N_3NaO_6S^+$ $[M+Na]^+$:1090.2734 found 1090.2734.

Compound 15. A solution of compound **6** (493.0 mg, 1.0 mmol), compound **S1** (240.0 mg, 1.2 mmol), EDC (287.6 mg, 1.5 mmol), HOBt (202.7 mg, 1.5 mmol), and Et_3N (515.1 μL, 3.7 mmol) in anhydrous DMF (10.0 mL) was stirred at room temperature for 12 h. The solvent was removed under vaccum. The crude product was purified by flash silica gel

column chromatography (20% MeOH in 1:1 DCM-Acetone) to give desired product **15** in 85% yield (558.2 mg). R_f = 0.25 (5% MeOH in 1:1 DCM-Acetone); ^{1}H NMR (400 MHz, MeOD) δ 4.93 (dd, *J* = 4.2, 7.8 Hz, 1H), 3.75 (s, 3H), 3.35 (dd, *J* = 4.4, 14.4 Hz, 1H), 3.25 (dd, *J* = 8.0, 14.4 Hz, 1H), 2.84 – 2.26 (m, 4H); ^{13}C NMR (100 MHz, MeOD) δ 172.8, 172.3, 53.2, 52.3, 51.1, 27.6 (t, *J* = 21.9 Hz), 27.5; ^{19}F NMR (376 MHz, CF_3COOH) δ -82.0 (t, *J* = 11.3 Hz, 3F), -115.0 – -115.3 (m, 2F), -122.0 – -122.2 (m, 2F), -122.2 – -122.5 (m, 4F), -123.1 – -123.4 (m, 2F), -123.8 – -124.1 (m, 2F), -126.6 – -126.9 (m, 2F); HRMS (ESI) m/z calculated for $C_{15}H_{11}F_{17}NO_6S^-$ $[M-H]^-$: 656.0036 found 656.0037.

2. Fabrication of fluorous-functionalized superparamagnetic nanoparticles (F_{tag}@MNPs)

Preparation of superparamagnetic nanoparticles (Fe_3O_4)

Superparamagnetic nanoparticles (Fe_3O_4) were prepared by the aqueous co-precipitation of $FeCl_2$ and $FeCl_3$ with a molar ratio of Fe(II)/Fe(III) = 0.5 under basic conditions (pH=11-12). In a round-bottom flask, $FeCl_3$ (5.2 g) and $FeCl_2$ (2.0 g) were dissolved in 25 mL of deoxygenated water (obtained by bubbling deionized water with a resistivity of 17.8 MΩ with nitrogen gas for at least 30 min) in the presence of HCl (12.1 N, 0.85 mL) with stirring. The resulting solution was added dropwise to a vigorously stirred NaOH (250 mL, 1.5 M) solution. A black precipitate formed almost immediately and was separated by centrifugation (4000 rpm) for 5 min. The Fe_3O_4 nanoparticles were washed by dispersing the precipitate in deoxygenated water, and the water layer was decanted after centrifugation (4000 rpm). After repeating the centrifugation-redispersion cycle three times, the precipitate was suspended in 300 mL of 0.01-N HCl with stirring to neutralize the nanoparticles' anionic charges. The cationic colloidal nanoparticles were once again

precipitated by centrifugation (4000 rpm), washed twice with deoxygenated water, and suspended in deionized water to yield superparamagnetic Fe_3O_4 nanoparticles.

Preparation of the water-compatible fluorous-functionalized MNPs (F_{tag}@MNPs)

A suspension of the above Fe_3O_4 nanoparticles (10 mL, 56 mg/mL) was dispersed in 1-propanol (100 mL), and the resulting nanoparticle solution was sonicated for 30 min to dissociate any potential aggregates. Then, 25% NH_4OH (7.62 mL) and TEOS (1.87 mL) were added to the mixture. The resulting solution was stirred at 60 °C for 2 h. Then, a mixture of mPEG and F_{tag}-$(OEt)_3$ at a volume ratio of 5:1 (1.87 mL:0.38 mL) was added, and the resulting solution was vigorously stirred at 60 °C for 12 h. After washing with 1-propanol (5 mL x 3) and ddH_2O (5 mL x 3), the F_{tag}@MNPs were lyophilized and stored at 4 °C until further use.

3. Protein overexpression, purification and modification

Protein overexpression and purification

Escherichia coli BL21 cells containing the construction vector were grown in Luria broth supplemented with ampicillin (100 μg/mL) at 37 °C until the optical density at 600 nm (OD_{600}) value reached 0.6-0.8. Gene expression was induced with isopropyl β-D-1-thiogalactopyranoside (IPTG) (0.5 mM), and the cells were grown at 16 °C for 16 h. The cells were harvested by centrifugation (5000 rpm) at 4 °C for 15 min and then lysed by ultrasonication in column buffer (20-mM Tris, 500-mM NaCl, 0.1-mM ethylenediaminetetraacetic acid [EDTA] and 0.1% Triton X-100, pH 8.0). The cell lysates were centrifuged (20000 x *g*) at 4 °C for 30 min to remove the cell debris and then poured into a chitin bead column. After incubating the chitin affinity beads with the cell lysate at

4 °C for 30 min, the resin was washed with column buffer (20-mM Tris, 500-mM NaCl, and 0.1-mM EDTA, pH 8.0).

Preparation of the *C*-MESNa target protein was conducted by on-column cleavage in the presence of MESNa (300 mM) in 6 mL of column buffer (20-mM Tris, 500-mM NaCl, and 0.1-mM EDTA, pH 8.0) at 4 °C for 16 h. The protein was eluted with elution buffer (20-mM Tris, 500-mM NaCl, and 0.1-mM EDTA, pH 8.0), and the fractions were analyzed by sodium dodecyl sulfate (SDS)-PAGE. The protein solution was concentrated and stored at -20 °C.

For native protein preparation, the chitin affinity beads were incubated with dithiothreitol (DTT) (80 mM) in 6 mL of column buffer (20-mM Tris, 500-mM NaCl, and 0.1-mM EDTA, pH 8.0) at 4 °C for 16 h. The fractions were pooled and dialyzed against column buffer at 4 °C to remove excess DTT.

Protein modification through NCL or boronate diester formation

For NCL protein modification, compound **1** (1 mM), tris(2-carboxyethyl)phosphine (TCEP) (2 mM), and MESNa (300 mM) were added to a 200 μL solution of Tris-HCl buffer (20-mM Tris, 500-mM NaCl, and 0.1-mM EDTA, pH 8.0) containing *C*-MESNa protein (10 μM) and incubated at 4 °C for 19 h. Then, the protein was purified using a size-exclusion column (PD MidiTrap G-25, GE) to remove the excess fluorous probe. The protein solution was concentrated (Amicon Ultra-0.45 mL, Millipore) and then stored at -20 °C.

For protein modification by boronate diester formation, a mixture of monoclonal rabbit anti-ricin alpha chain (anti-RAC) antibody (EY Lab) (5 μg/mL, final concentration) and F_{tag}-BA (**2**) (1 mM, final concentration) in 50 μL of binding buffer (20-mM Tris, 500-mM

NaCl, and 0.1-mM EDTA, pH 8.0) was incubated at 4 °C for 16 h. After incubation, the boronated antibody was purified from the fluorous small-molecular probes using a PD MidiTrap G-25 size-exclusion column. The protein solution was concentrated (Amicon Ultra-0.45 mL, Millipore) and then stored at -20 °C until use.

Analysis of the protein by 10% native-PAGE

The protein solution and 2X loading dye were mixed at a 1:1 ratio and then heated to 100 °C for 10 min. The proteins were subsequently separated by 10% native-PAGE at 110 V for 2 h. The recipes for the native-PAGE and running buffer are presented in Table S1.

Protein analysis by SDS-PAGE

The protein solution and 2X loading dye were mixed at a 1:1 ratio and then heated to 100 °C for 10 min. The proteins were subsequently separated by standard 10% SDS-PAGE at 110 V for 2 h.

Table S1. The recipe for native-PAGE and running buffer.

	10% native-PAGE	
	Stacking gel	Separating gel
H2O	4.1 mL	3.45 mL
30% acrylamide solution (29:1)	3.3 mL	0.83 mL
1.5 M Tris (pH 8.8)	2.5 mL	—
1.0 M Tris (pH 6.8)	—	0.63 mL
10% APS	0.1 mL	0.05 mL
TEMED	0.004 mL	0.005 mL

Purification of the perfluoro-tagged proteins with F_{tag}@MNPs

The F_{tag}@MNPs (10 mg) were first dispersed in 0.05% phosphate-buffered saline with Tween 20 (PBST) buffer (5 mL), washed with HEPES buffer (5 mL x3), and then suspended in HEPES buffer (0.5 mL). A protein mixture of MBP and F_{tag}-MBP (total 100 μg) was added to the F_{tag}@MNPs and allowed to react at 25 °C for 5 min to allow the MNPs to capture the F_{tag} proteins. After capture, the F_{tag}@MNPs were washed three times with 1 mL of HEPES buffer to remove the untagged MBP. Compound **5** (1 mM in HEPES buffer) was then added to the F_{tag}@MNP solution and allowed to react at 25 °C for 5 min. The desired F_{tag}-MBPs were obtained by first trapping the F_{tag}@MNPs at the bottom of the tube using a strong magnet and then retrieving the supernatant solution. The F_{tag}-MBPs were further separated from the fluorous small-molecule probe **5** using a PD MidiTrap G-25 size-exclusion column.

4. Immobilization of the perfluoro-tagged protein on a glass slide by fluorous-fluorous interaction

To analyze the immobilized MBP, the slide was incubated with biotinylated anti-MBP antibody (1 ng/μL, Vector Lab) for 3 h at room temperature. After decanting the solution, the slide was washed with 1% BSA in PBS (5 min) and deionized water (5 min). Following staining with streptavidin-Cy3 (10 ng/μL, Sigma-Aldrich) at 4 °C for 30 min, the slide was washed with 1% BSA in PBS (5 min). After a final wash with deionized water (5 min), the fluorescence signal was measured with a VIDAR Revolution® 4550 scanner using a Cy3 filter.

To detect the GST activity, the slide was incubated with biotinylated anti-GST (10 ng/μL, Novus Biological) at room temperature for 3 h. After being washed as described

above, the immobilized GST proteins were then stained with streptavidin-Cy3 (10 ng/μL, Sigma) at 4 °C for 30 min. After washing, the fluorescence signal was detected as described above.

To detect the activities of the immobilized anti-RAC Ab microarrays, the slide was first incubated with RCA_{120} (1 μg/μL, Sigma-Aldrich) at room temperature for 2 h. After being washed as described above, the slide was incubated with biotinylated anti-RCA_{120} antibody (10 ng/μL, Novus Biological) at room temperature for 3 h. After staining with streptavidin-Cy3 (10 ng/μL, Sigma) at 4 °C for 30 min, the fluorescence signal was measured as described above.

Protein concentration required for protein microarray fabrication

Different concentrations of fluorous-MBPs (0.05-0.4 μg/μL) in printing buffer were spotted on a fluorous-coated glass slide. The slide was then incubated with biotinylated anti-MBP antibody (1 ng/μL, Vector Lab) at room temperature for 3 h, stained with streptavidin-Cy3 (10 ng/μL, Sigma) at 4 °C for 30 min, and subjected to fluorescence detection.

B. Supplementary Figures

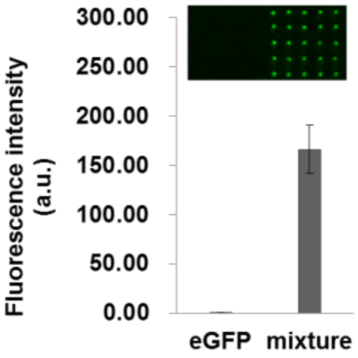

Figure S1. Fluorescence images of native eGFP and a mixture of eGFP and perfluorotagged-eGFP spotted on a microarray slide.

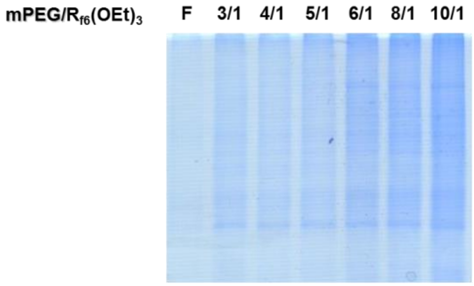

Figure S2. F_{tag}@MNPs non-specific adsorption assay by 10% SDS-PAGE.

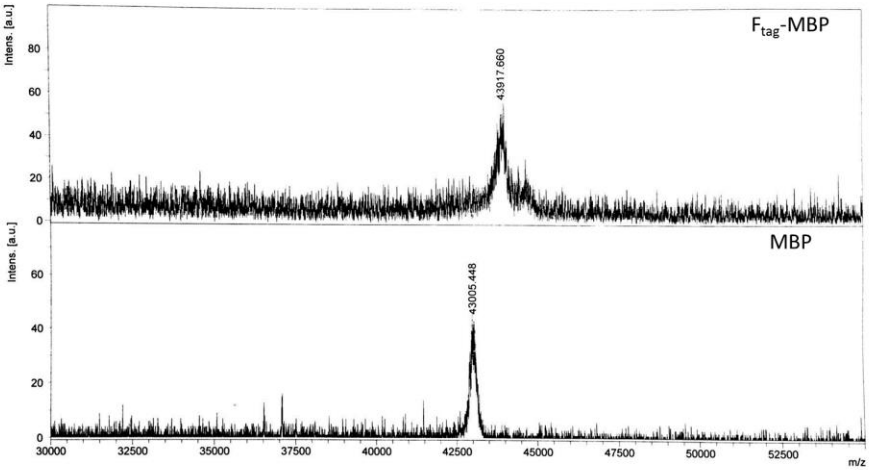

Figure S3. Mass of F_{tag}-MBP_F observed by MALDI-TOF MS analysis

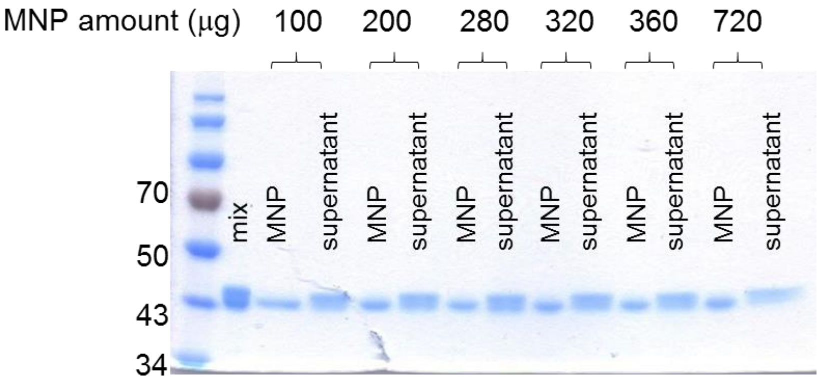

Figure S4. F_{tag}-MBP was enriched by various amounts of F_{tag}@MNPs (left to right in μg; 100, 200, 280, 320, 360, and 720) from a mixture of MBP (100 μg) and F_{tag}-MBP (5 μg). The results were analyzed by 10% native PAGE.

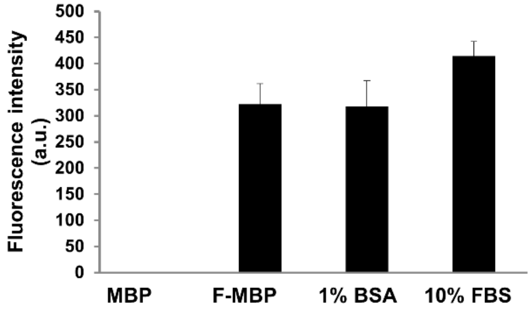

Figure S5. Stability of perfluorotagged-MBP on fluorous slide. We observed no significant change in fluorescent signal after incubation with 1% BSA or 10% FBS for 1h, suggesting that the interaction is indeed stable enough even under complex conditions like serum samples.

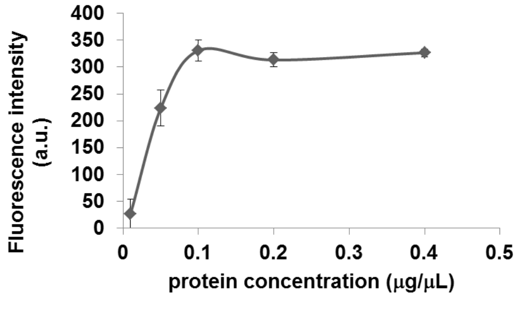

Figure S6. Immobilization and detection of purified perfluorotagged-MBP on a fluorous slide. Various concentrations of perfluorotagged-MBP (left to right in μg/μL; 0.4, 0.2, 0.1, 0.05, and 0.01) were tested.

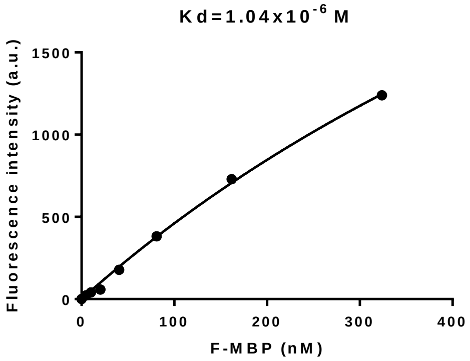

Figure S7. A series of 8 different concentrations of F_{tag}-MBP were spotted on the fluorous-microarray slide to obtain the estimated binding affinity Kd = 1.04 x 10^{-6} M.

C. Spectrum

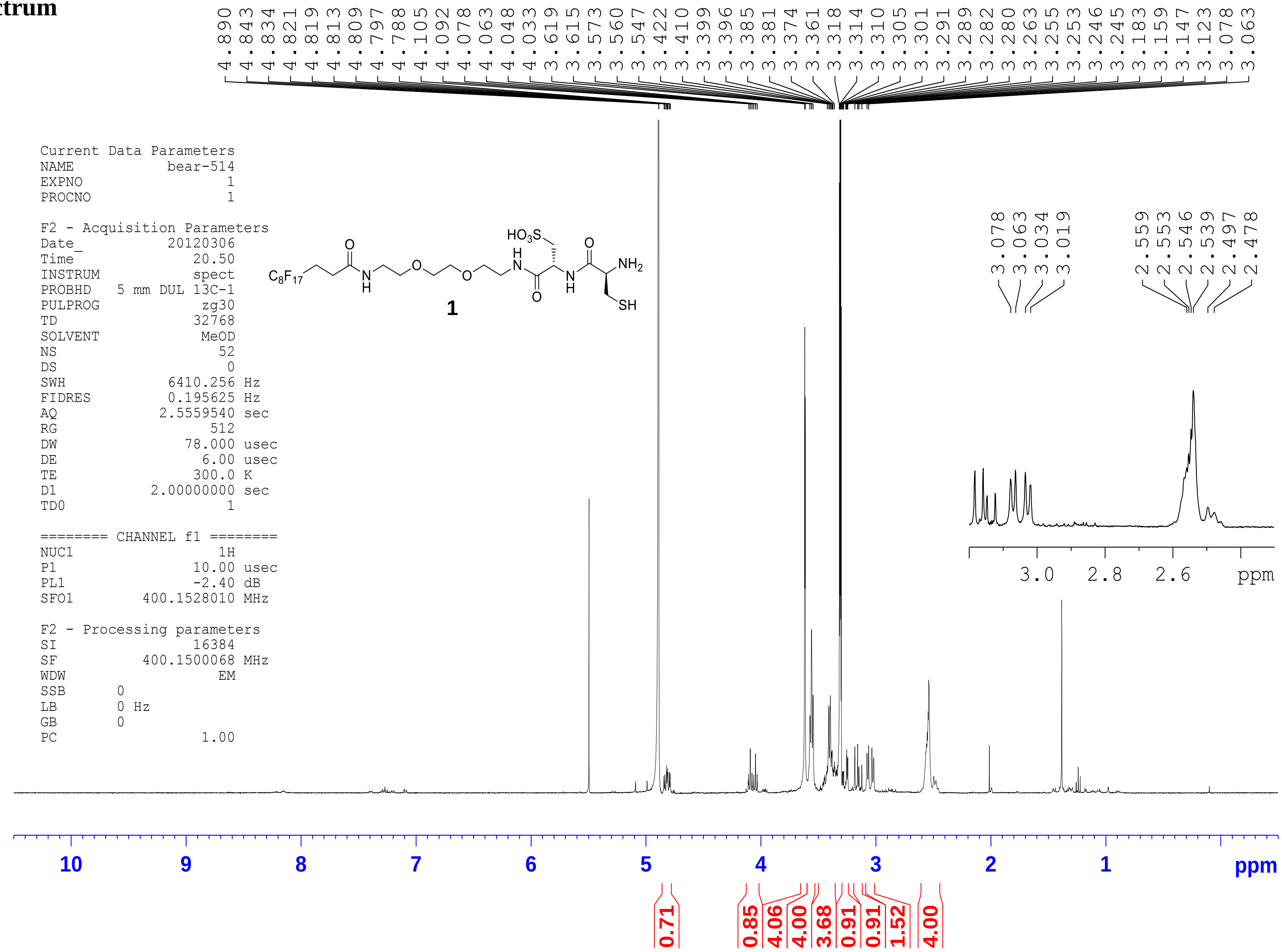
4.890 4.843 4.834 4.821 4.819 4.813 4.809 4.797 4.788 4.105 4.092 4.078 4.063 4.048 4.033 3.619 3.615 3.573 3.560 3.547 3.422 3.410 3.399 3.396 3.385 3.381 3.374 3.361 3.318 3.314 3.310 3.305 3.301 3.291 3.289 3.282 3.280 3.263 3.255 3.253 3.246 3.245 3.183 3.159 3.147 3.123 3.078 3.063
Current Data Parameters
NAME bear-514
EXPNO 1
PROCNO 1
F2 - Acquisition Parameters
Date_ 20120306
Time 20.50
INSTRUM spect
PROBHD 5 mm DUL 13C-1
PULPROG zg30
TD 32768
SOLVENT MeOD
NS 52
DS 0
SWH 6410.256 Hz
FIDRES 0.195625 Hz
AQ 2.5559540 sec
RG 512
DW 78.000 usec
DE 6.00 usec
TE 300.0 K
D1 2.00000000 sec
TD0 1
======== CHANNEL f1 ========
NUC1 1H
P1 10.00 usec
PL1 -2.40 dB
SFO1 400.1528010 MHz
F2 - Processing parameters
SI 16384
SF 400.1500068 MHz
WDW EM
SSB 0
LB 0 Hz
GB 0
PC 1.00
C_8F_{17}
HO_3S
NH_2
SH
1
3.078 3.063 3.034 3.019
2.559 2.553 2.546 2.539 2.497 2.478
3.0 2.8 2.6 ppm
10 9 8 7 6 5 4 3 2 1 ppm
0.71 0.85 4.06 4.00 3.68 0.91 0.91 1.52 4.00

Current Data Parameters
NAME 20160609-Bear-Rf8-PEG-Cysteic-cys-13C
EXPNO 2
PROCNO 1

F2 - Processing parameters
SI 65536
SF 100.5217096 MHz
WDW EM
SSB 0
LB 0.30 Hz
GB 0
PC 1.00

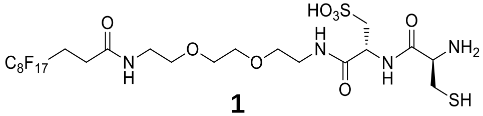

1

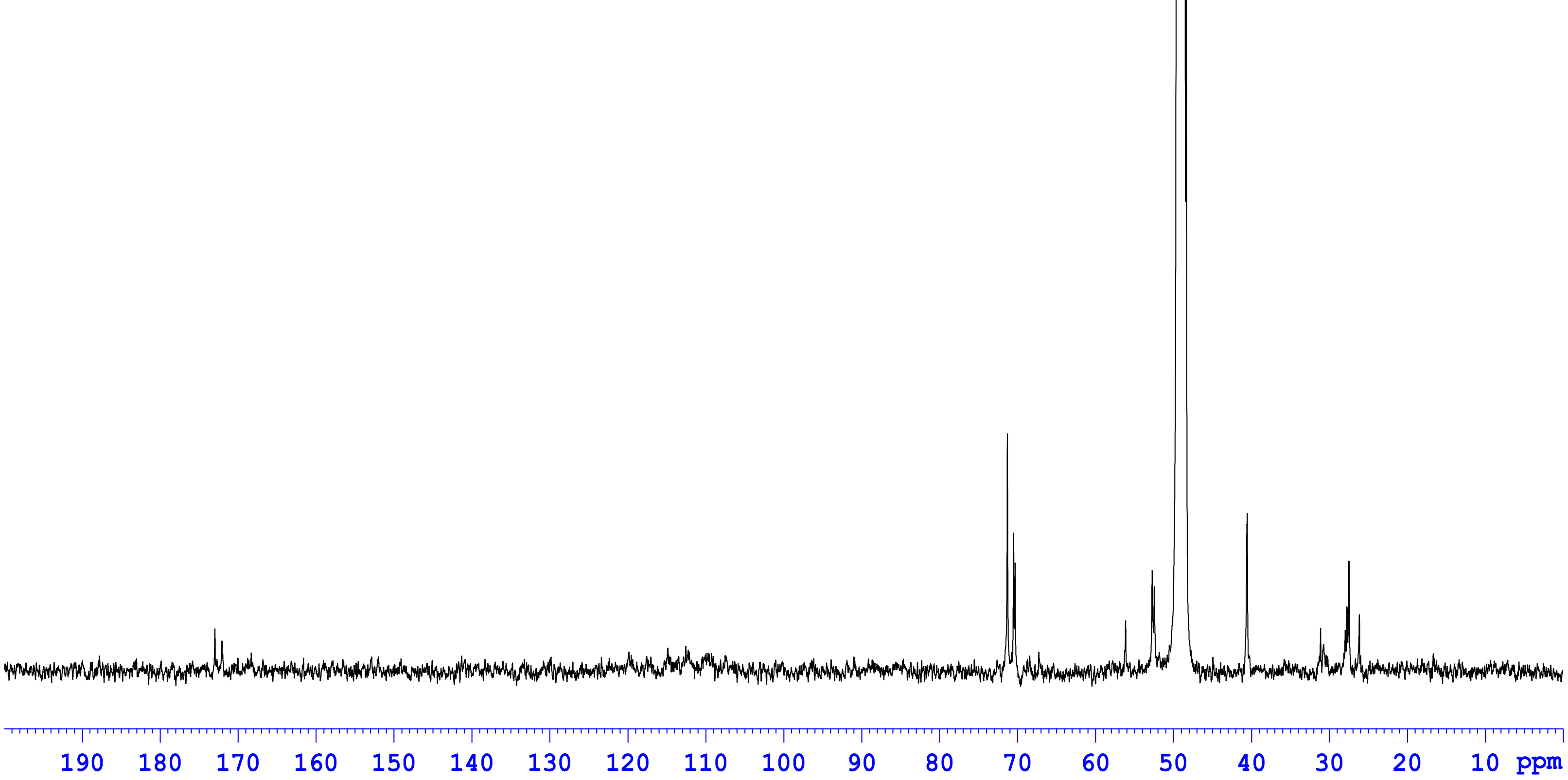

```
Current Data Parameters
NAME     20160609-Bear-Rf8-PEG-Cysteic-cys-19F
EXPNO                   2
PROCNO                  1

F2 - Processing parameters
SI               131072
SF          376.1621068 MHz
WDW                  EM
SSB      0
LB                 0.30 Hz
GB       0
PC                 1.00
```

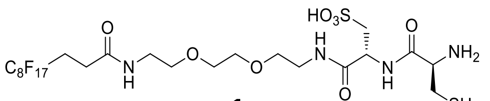

1

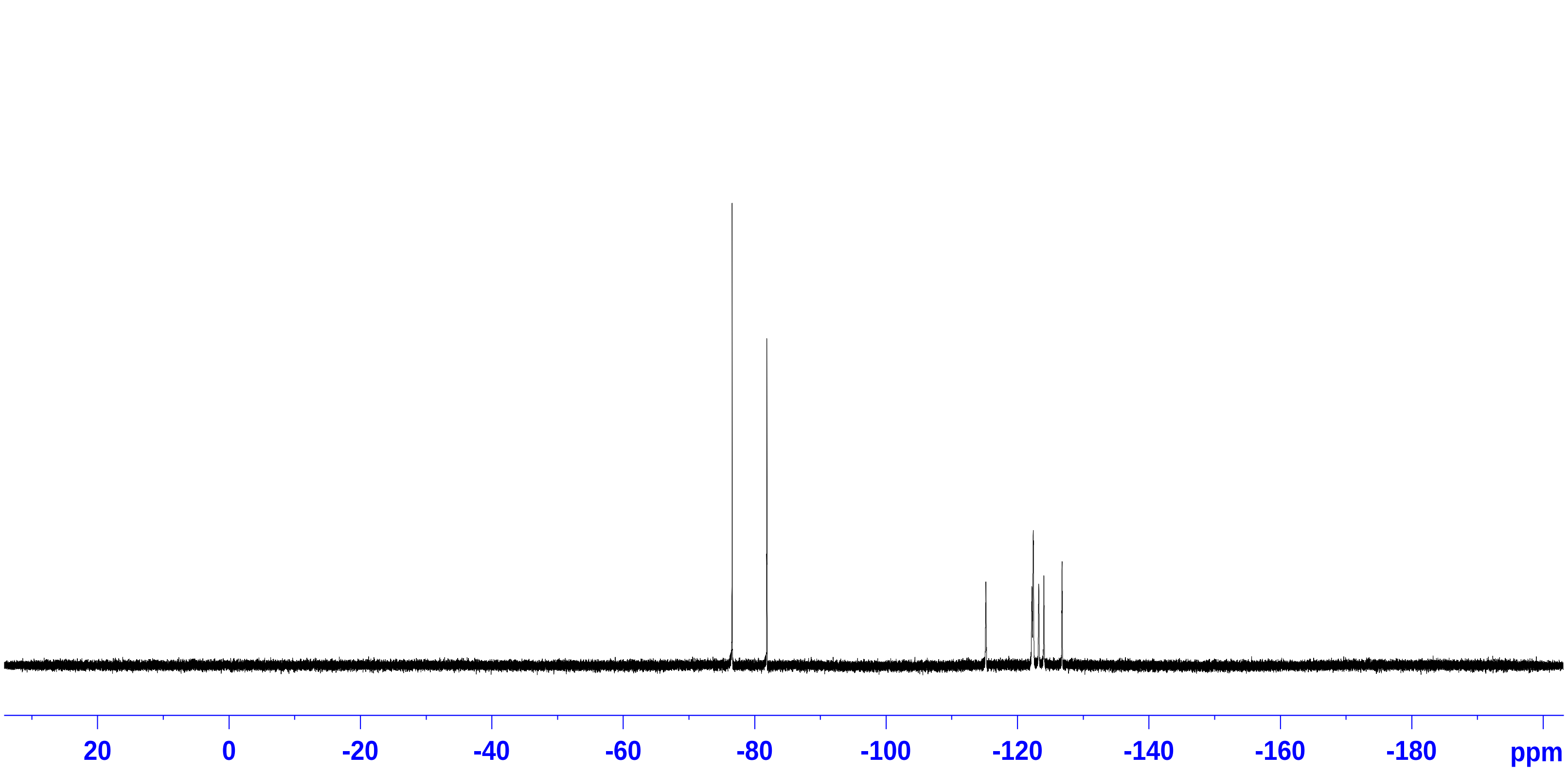

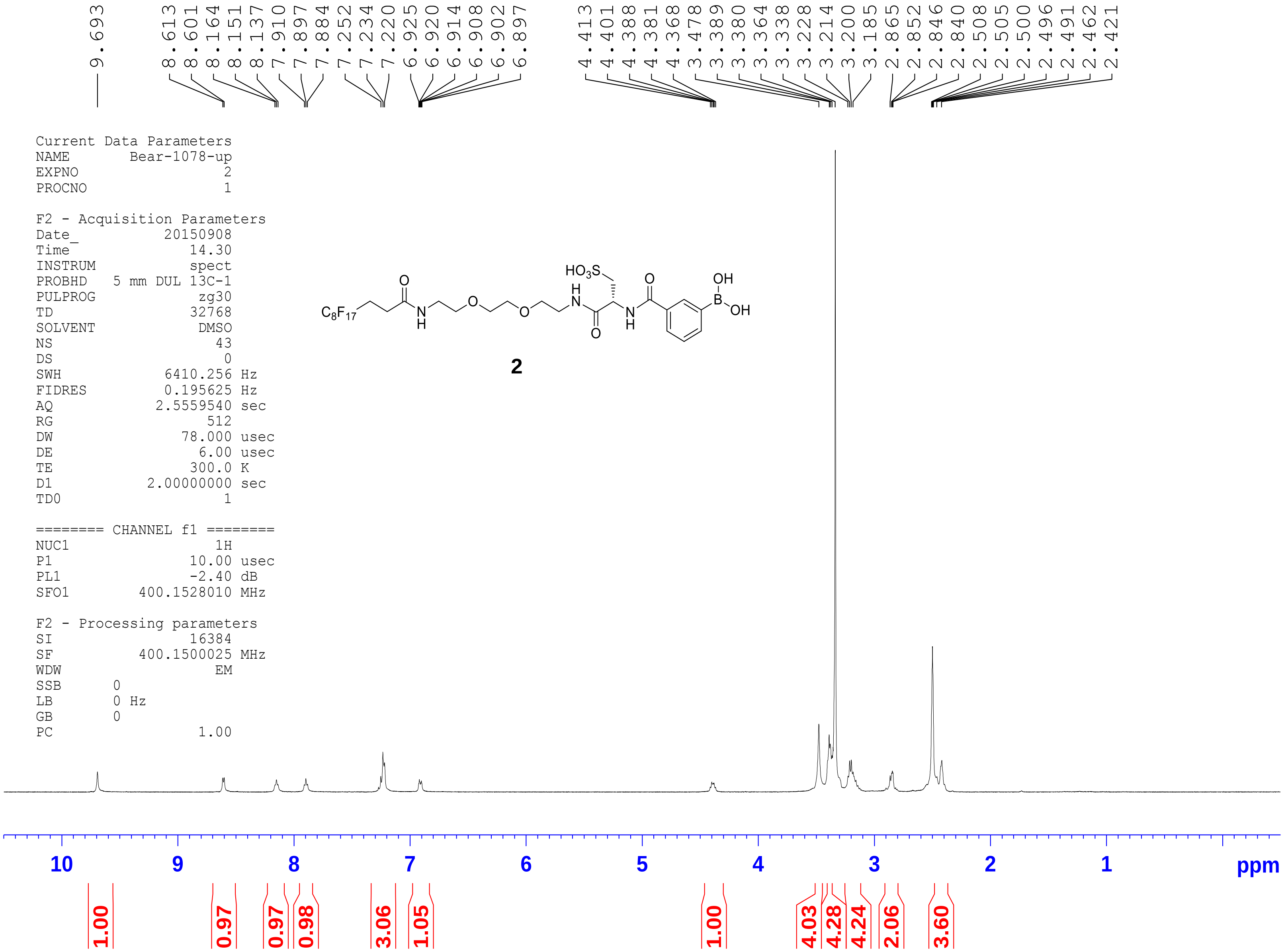

Current Data Parameters
NAME Bear-1078-up
EXPNO 2
PROCNO 1

F2 - Acquisition Parameters
Date_ 20150908
Time 14.30
INSTRUM spect
PROBHD 5 mm DUL 13C-1
PULPROG zg30
TD 32768
SOLVENT DMSO
NS 43
DS 0
SWH 6410.256 Hz
FIDRES 0.195625 Hz
AQ 2.5559540 sec
RG 512
DW 78.000 usec
DE 6.00 usec
TE 300.0 K
D1 2.00000000 sec
TD0 1

======== CHANNEL f1 ========
NUC1 1H
P1 10.00 usec
PL1 -2.40 dB
SFO1 400.1528010 MHz

F2 - Processing parameters
SI 16384
SF 400.1500025 MHz
WDW EM
SSB 0
LB 0 Hz
GB 0
PC 1.00

2

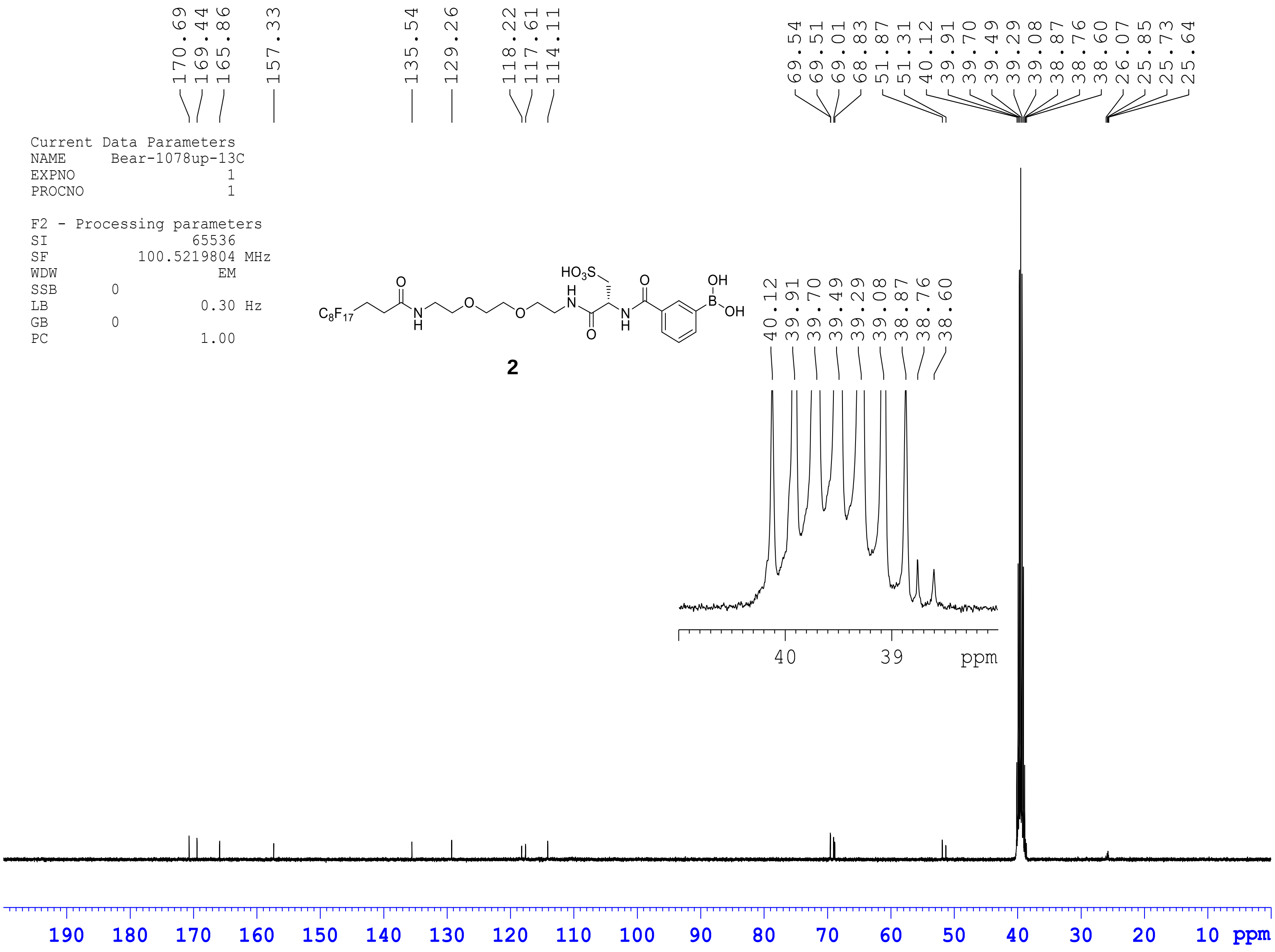
170.69
169.44
165.86
157.33
135.54
129.26
118.22
117.61
114.11
69.54
69.51
69.01
68.83
51.87
51.31
40.12
39.91
39.70
39.49
39.29
39.08
38.87
38.76
38.60
26.07
25.85
25.73
25.64
Current Data Parameters
NAME Bear-1078up-13C
EXPNO 1
PROCNO 1
F2 - Processing parameters
SI 65536
SF 100.5219804 MHz
WDW EM
SSB 0
LB 0.30 Hz
GB 0
PC 1.00
2
40 39 ppm
190 180 170 160 150 140 130 120 110 100 90 80 70 60 50 40 30 20 10 ppm

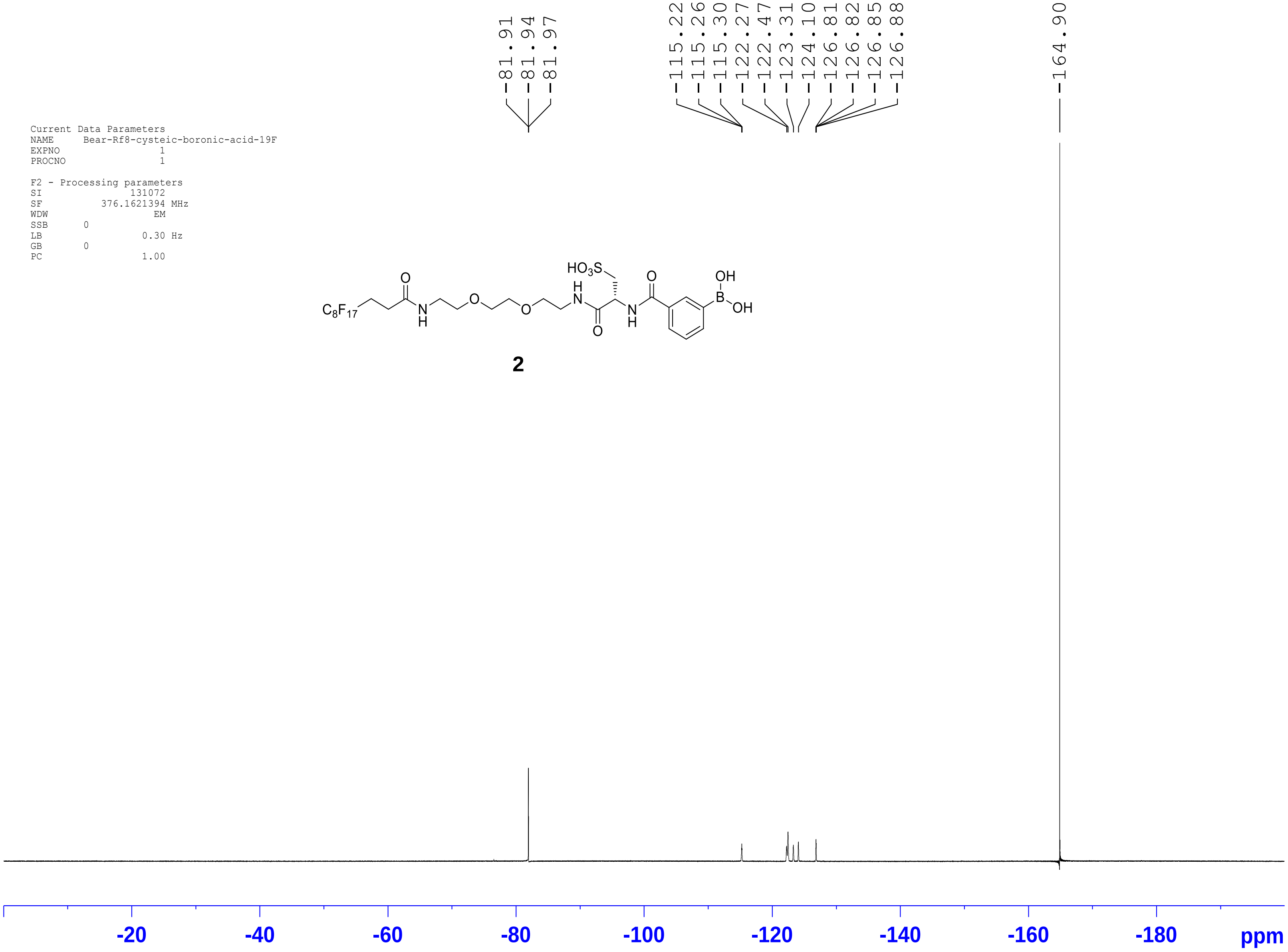
Current Data Parameters
NAME Bear-Rf8-cysteic-boronic-acid-19F
EXPNO 1
PROCNO 1
F2 - Processing parameters
SI 131072
SF 376.1621394 MHz
WDW EM
SSB 0
LB 0.30 Hz
GB 0
PC 1.00
-81.91
-81.94
-81.97
-115.22
-115.26
-115.30
-122.27
-122.47
-123.31
-124.10
-126.81
-126.82
-126.85
-126.88
-164.90
C_8F_{17}
O
H
N
O
O
H
N
HO_3S
O
N
H
O
OH
B
OH
2
-20
-40
-60
-80
-100
-120
-140
-160
-180
ppm

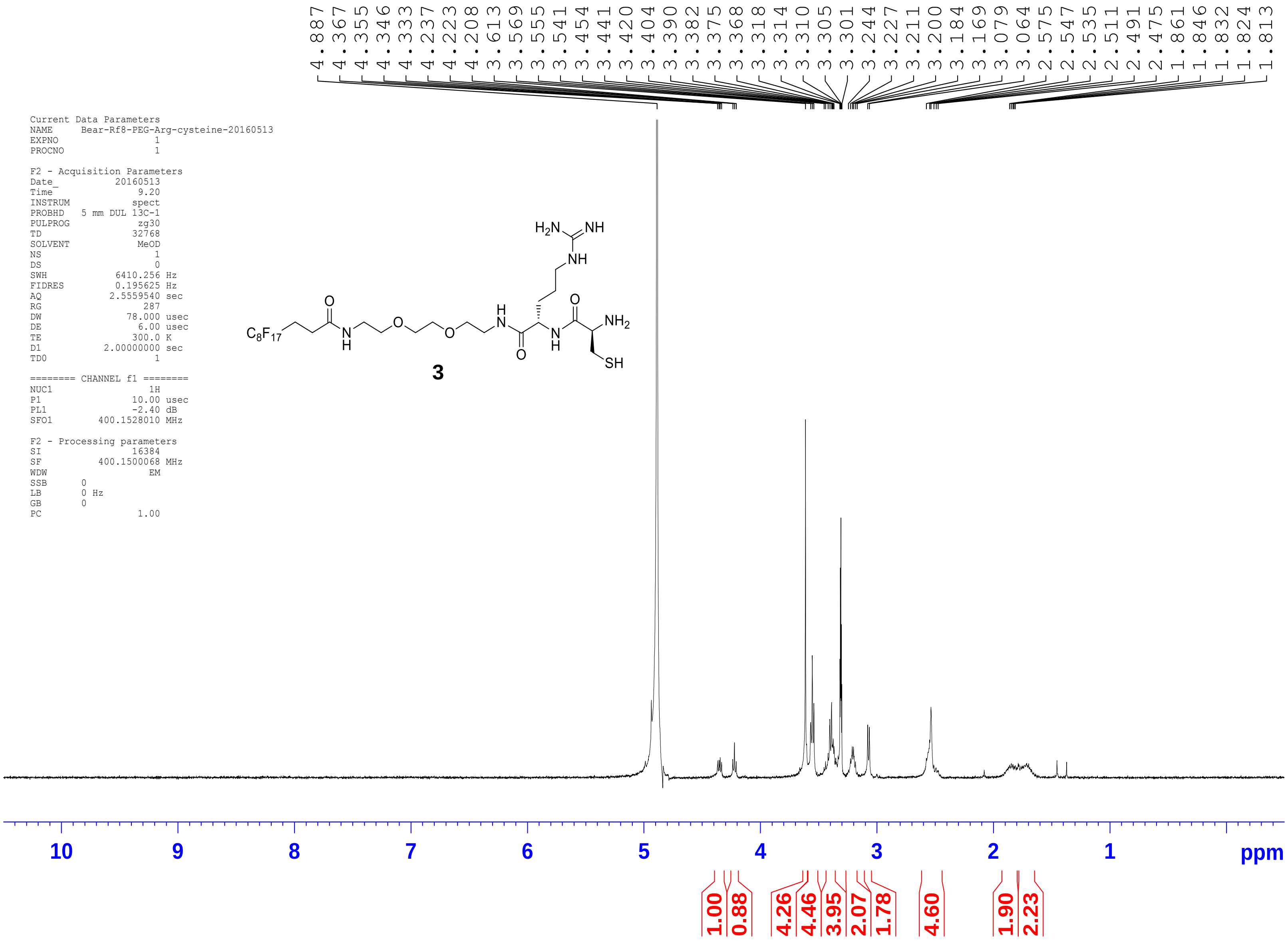
4.887 4.367 4.355 4.346 4.333 4.237 4.223 4.208 3.613 3.569 3.555 3.541 3.454 3.441 3.420 3.404 3.390 3.382 3.375 3.368 3.318 3.314 3.310 3.305 3.301 3.244 3.227 3.211 3.200 3.184 3.169 3.079 3.064 2.575 2.547 2.535 2.511 2.491 2.475 1.861 1.846 1.832 1.824 1.813
Current Data Parameters
NAME Bear-Rf8-PEG-Arg-cysteine-20160513
EXPNO 1
PROCNO 1
F2 - Acquisition Parameters
Date_ 20160513
Time 9.20
INSTRUM spect
PROBHD 5 mm DUL 13C-1
PULPROG zg30
TD 32768
SOLVENT MeOD
NS 1
DS 0
SWH 6410.256 Hz
FIDRES 0.195625 Hz
AQ 2.5559540 sec
RG 287
DW 78.000 usec
DE 6.00 usec
TE 300.0 K
D1 2.00000000 sec
TD0 1
======== CHANNEL f1 ========
NUC1 1H
P1 10.00 usec
PL1 -2.40 dB
SFO1 400.1528010 MHz
F2 - Processing parameters
SI 16384
SF 400.1500068 MHz
WDW EM
SSB 0
LB 0 Hz
GB 0
PC 1.00
H2N NH NH O O H N NH2 C8F17 N H O O N O N H SH
3
10 9 8 7 6 5 4 3 2 1 ppm
1.00 0.88 4.26 4.46 3.95 2.07 1.78 4.60 1.90 2.23

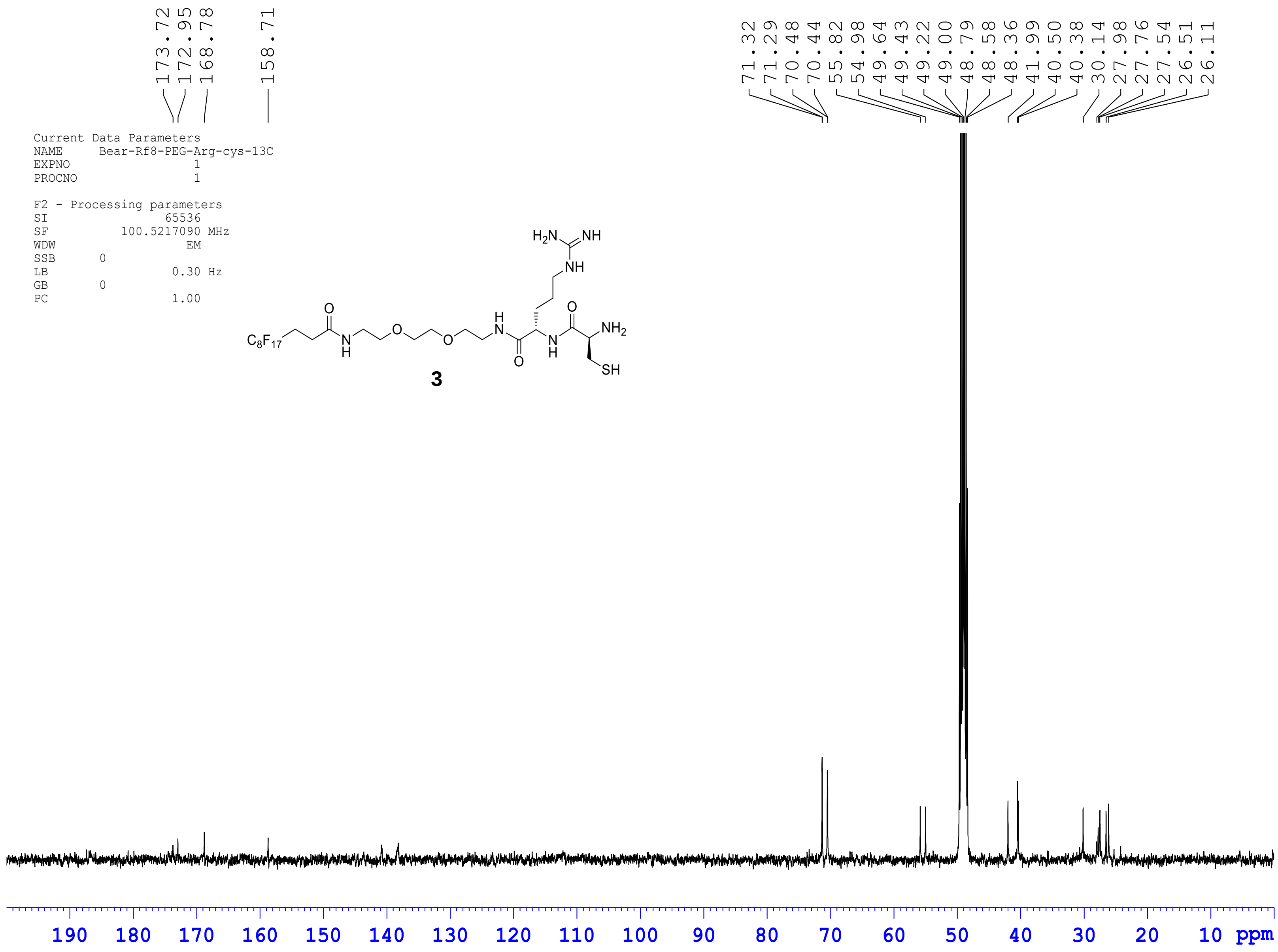
173.72
172.95
168.78
158.71
71.32
71.29
70.48
70.44
55.82
54.98
49.64
49.43
49.22
49.00
48.79
48.58
48.36
41.99
40.50
40.38
30.14
27.98
27.76
27.54
26.51
26.11
Current Data Parameters
NAME Bear-Rf8-PEG-Arg-cys-13C
EXPNO 1
PROCNO 1
F2 - Processing parameters
SI 65536
SF 100.5217090 MHz
WDW EM
SSB 0
LB 0.30 Hz
GB 0
PC 1.00
H2N
NH
NH
O
C8F17
N
H
O
O
H
N
O
N
H
O
NH2
SH
3
190 180 170 160 150 140 130 120 110 100 90 80 70 60 50 40 30 20 10 ppm

```
Current Data Parameters
NAME     Bear-Rf8-PEG-Arg-Cys-19F
EXPNO                  1
PROCNO                 1

F2 - Processing parameters
SI              131072
SF          376.1621319 MHz
WDW                  EM
SSB      0
LB                0.30 Hz
GB       0
PC                1.00
```

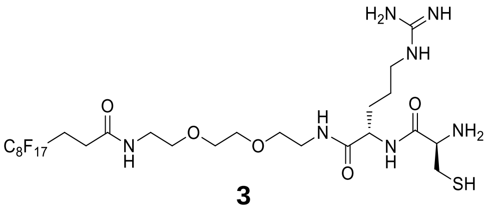

3

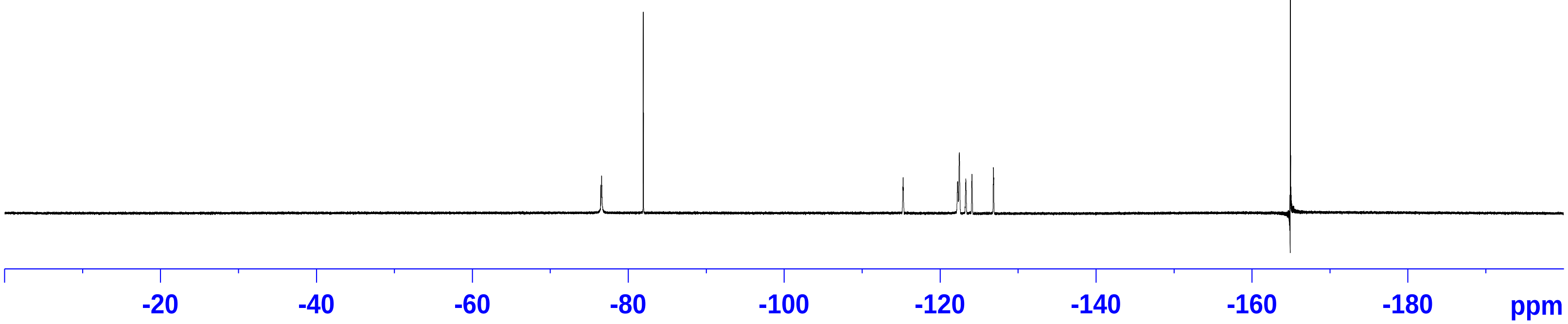

```
Current Data Parameters
NAME     Bear-Rf8-PEG-Cys-20160513
EXPNO                   1
PROCNO                  1

F2 - Acquisition Parameters
Date_          20160513
Time              13.04
INSTRUM           spect
PROBHD   5 mm DUL 13C-1
PULPROG            zg30
TD                32768
SOLVENT            MeOD
NS                   19
DS                    0
SWH            6410.256 Hz
FIDRES         0.195625 Hz
AQ            2.5559540 sec
RG                  203
DW               78.000 usec
DE                 6.00 usec
TE                300.0 K
D1           2.00000000 sec
TD0                   1

======== CHANNEL f1 ========
NUC1                 1H
P1                10.00 usec
PL1               -2.40 dB
SFO1        400.1528010 MHz

F2 - Processing parameters
SI                16384
SF          400.1500068 MHz
WDW                  EM
SSB      0
LB       0 Hz
GB       0
PC                 1.00
```

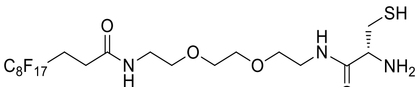

4

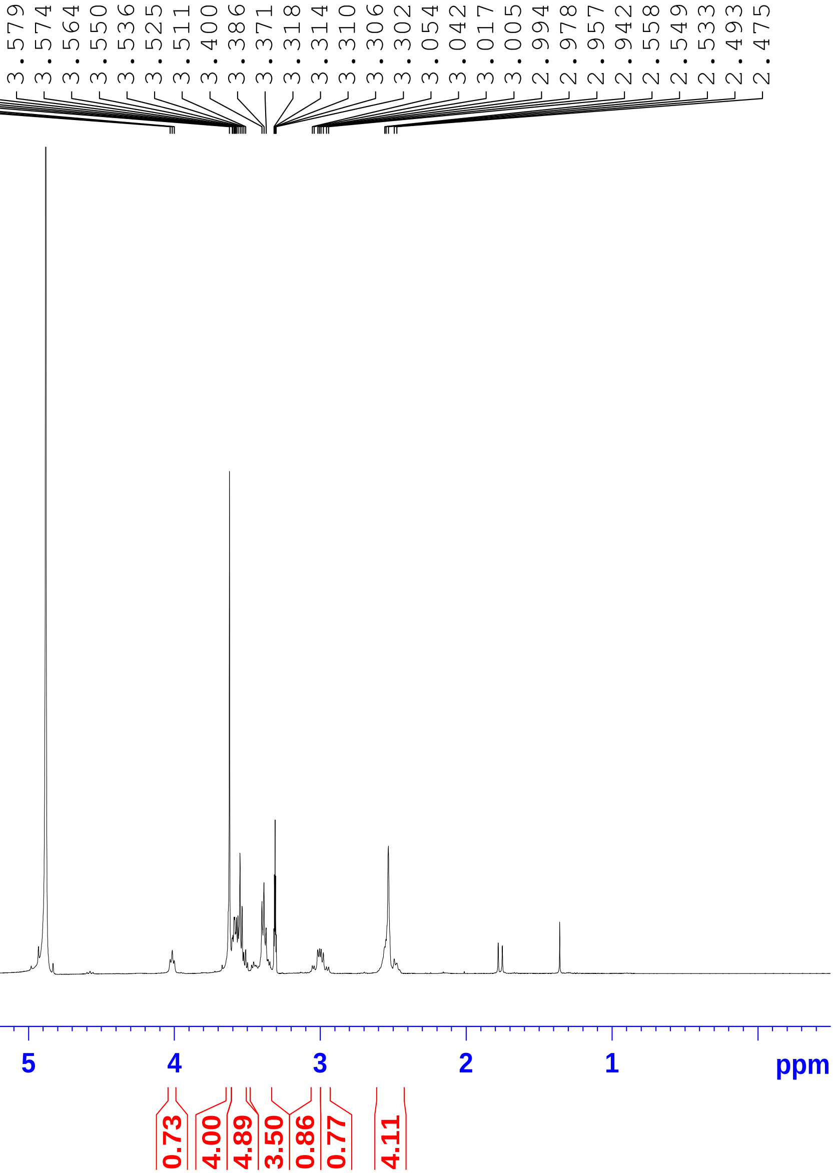

```
Current Data Parameters
NAME     Bear-Rf8-PEG-cysteine-20160513
EXPNO                  2
PROCNO                 1

F2 - Acquisition Parameters
Date_          20160513
Time               9.09
INSTRUM           spect
PROBHD   5 mm DUL 13C-1
PULPROG          zgpg30
TD                65536
SOLVENT            MeOD
NS                  100
DS                    0
SWH          22727.273 Hz
FIDRES        0.346791 Hz
AQ           1.4418420 sec
RG                   57
DW              22.000 usec
DE                6.00 usec
TE               300.0 K
D1          2.00000000 sec
d11         0.03000000 sec
DELTA       1.89999998 sec
TD0                   1

======== CHANNEL f1 ========
NUC1                13C
P1                 9.70 usec
PL1               -0.50 dB
SFO1        100.6288660 MHz

======== CHANNEL f2 ========
CPDPRG2         waltz16
NUC2                 1H
PCPD2             90.00 usec
PL2               -2.40 dB
PL12              15.10 dB
PL13              18.10 dB
SFO2        400.1516010 MHz

F2 - Processing parameters
SI                32768
SF          100.6176563 MHz
WDW                  EM
SSB      0
LB                 3.00 Hz
GB       0
PC                 1.00
```

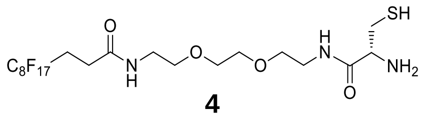

4

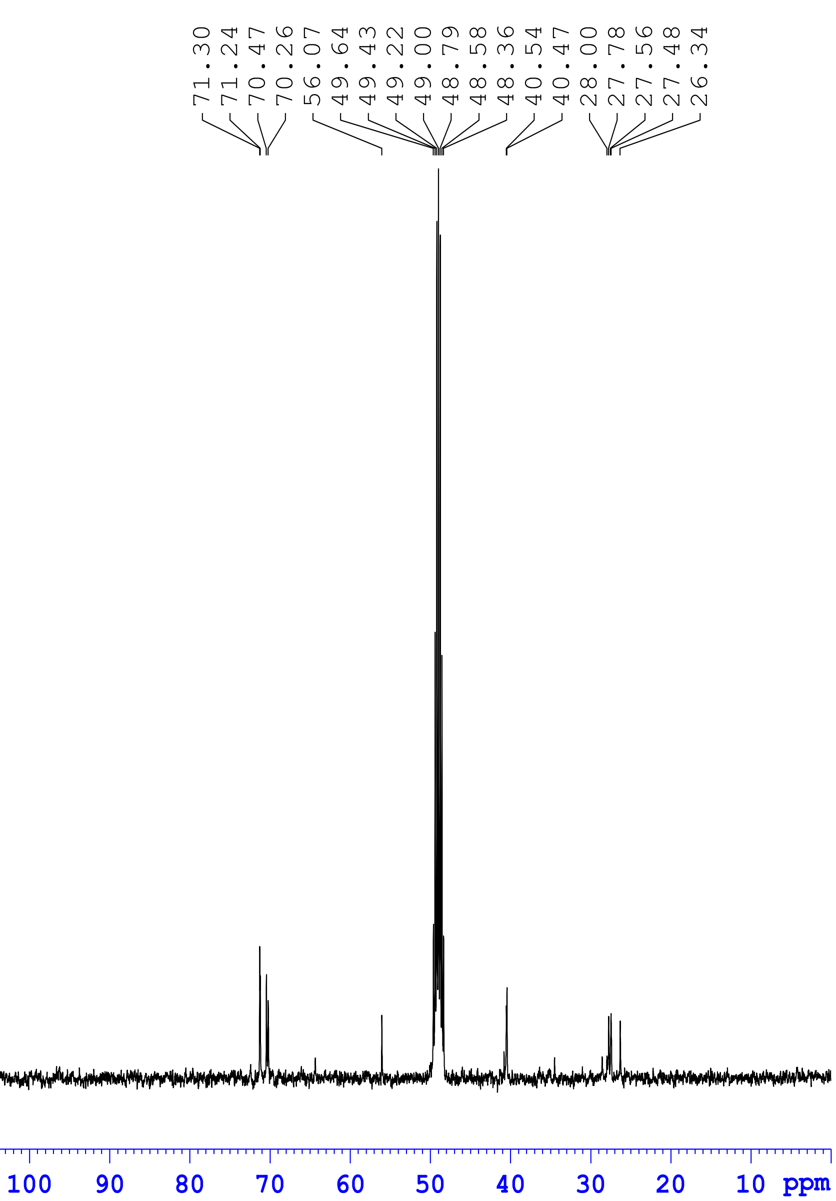

```
Current Data Parameters
NAME     20160609-Bear-Rf8-PEG-Cys-19F
EXPNO                   1
PROCNO                  1

F2 - Processing parameters
SI                 131072
SF          376.1621209 MHz
WDW                    EM
SSB      0
LB                   0.30 Hz
GB       0
PC                   1.00
```

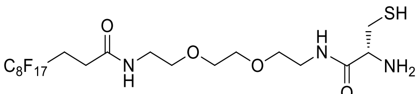

4

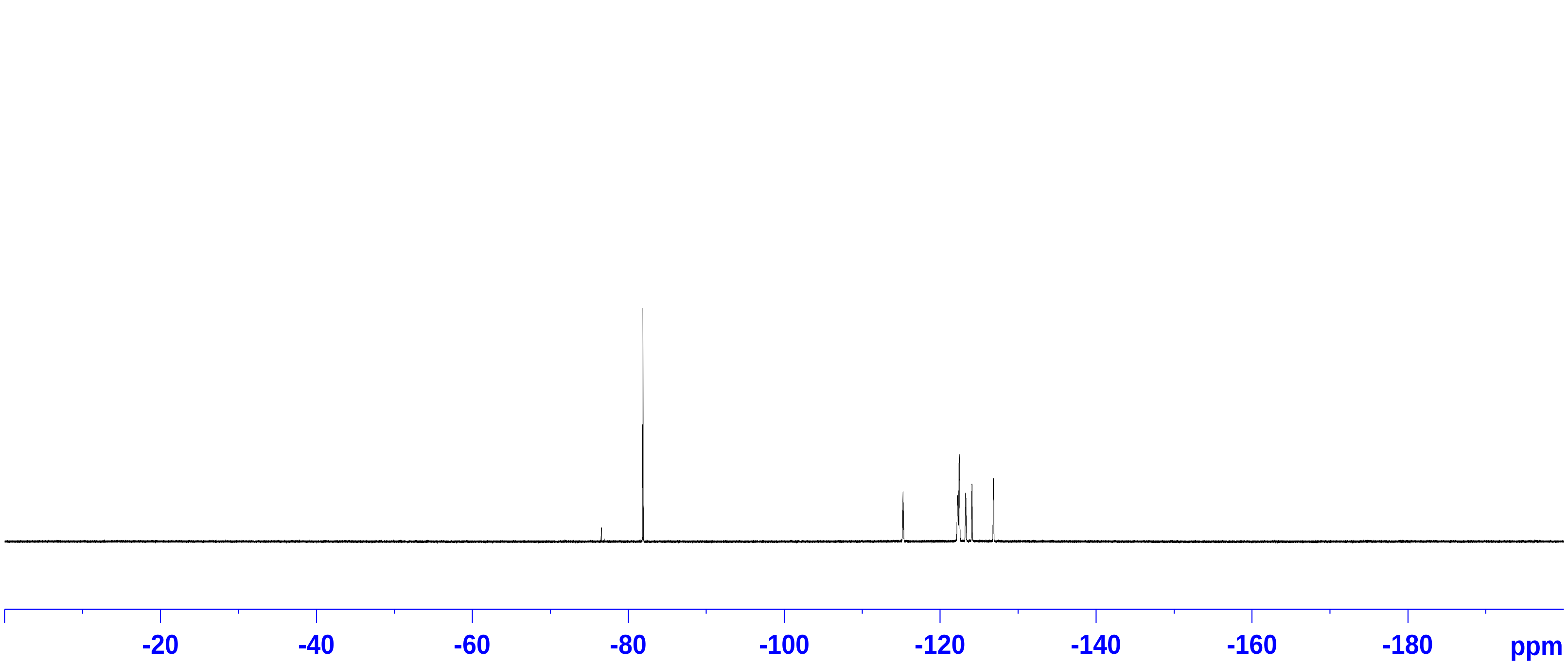

```
Current Data Parameters
NAME     Bear-Rf8-cysteic acid-20160513
EXPNO                  3
PROCNO                 1

F2 - Acquisition Parameters
Date_          20160513
Time              17.05
INSTRUM           spect
PROBHD   5 mm DUL 13C-1
PULPROG            zg30
TD                32768
SOLVENT            MeOD
NS                   11
DS                    0
SWH            6410.256 Hz
FIDRES         0.195625 Hz
AQ            2.5559540 sec
RG                  362
DW               78.000 usec
DE                 6.00 usec
TE                300.0 K
D1           2.00000000 sec
TD0                   1

======== CHANNEL f1 ========
NUC1                 1H
P1                10.00 usec
PL1               -2.40 dB
SFO1        400.1528010 MHz

F2 - Processing parameters
SI                16384
SF          400.1500068 MHz
WDW                  EM
SSB      0
LB       0 Hz
GB       0
PC                 1.00
```

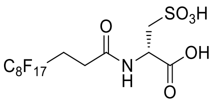

5

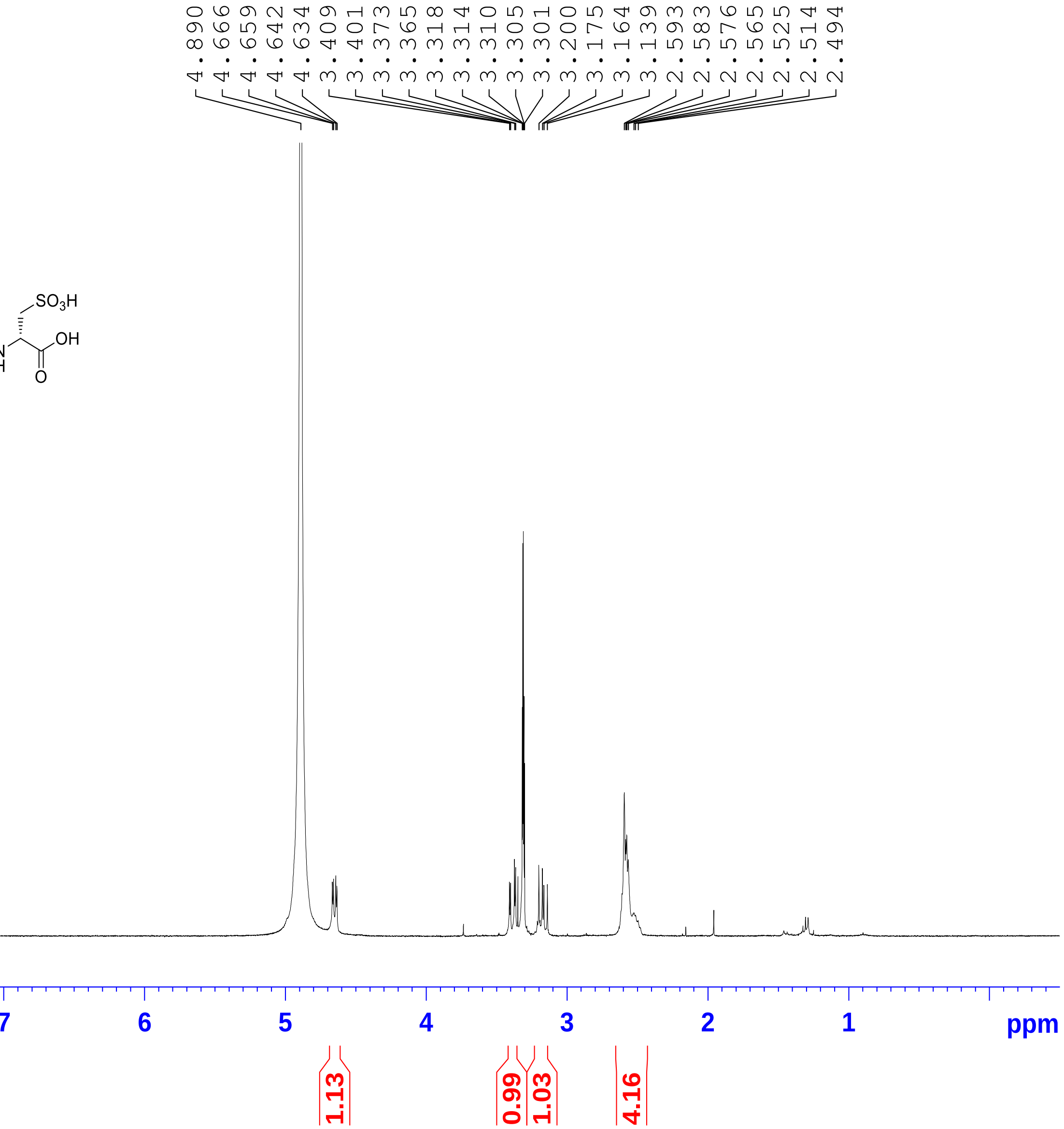

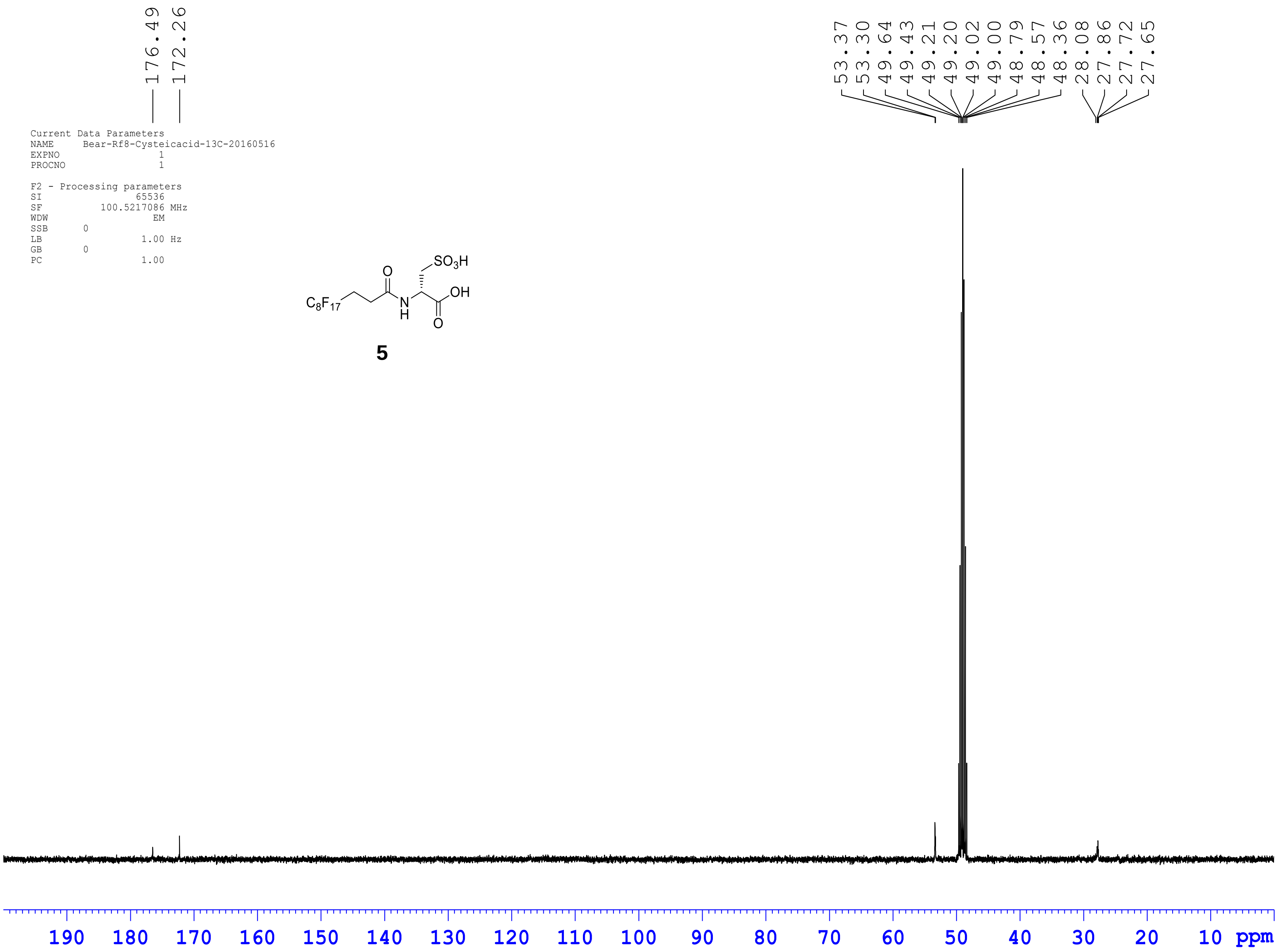
176.49
172.26
53.37
53.30
49.64
49.43
49.21
49.20
49.02
49.00
48.79
48.57
48.36
28.08
27.86
27.72
27.65
Current Data Parameters
NAME Bear-Rf8-Cysteicacid-13C-20160516
EXPNO 1
PROCNO 1
F2 - Processing parameters
SI 65536
SF 100.5217086 MHz
WDW EM
SSB 0
LB 1.00 Hz
GB 0
PC 1.00
SO_3H
O
OH
C_8F_{17}
N
H
O
5
190 180 170 160 150 140 130 120 110 100 90 80 70 60 50 40 30 20 10 ppm

```
Current Data Parameters
NAME     Rf8-cysteic-acid-19F-20160727
EXPNO                     1
PROCNO                    1

F2 - Processing parameters
SI                  131072
SF            376.1621295 MHz
WDW                     EM
SSB      0
LB                    0.30 Hz
GB       0
PC                    1.00
```

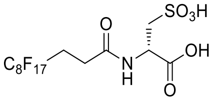

5

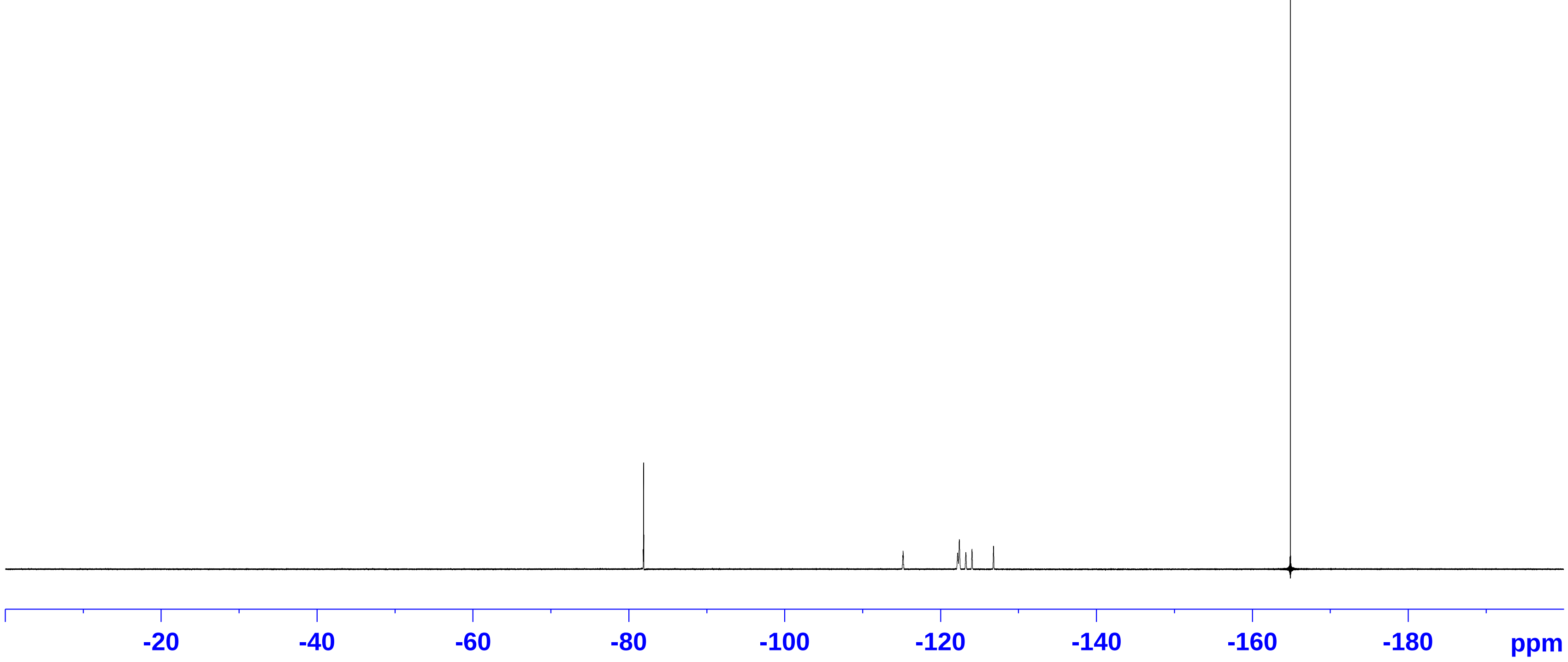

```
Current Data Parameters
NAME     Bear-cystenic acid methylester
EXPNO                     5
PROCNO                    1

F2 - Acquisition Parameters
Date_          20160308
Time               1.15
INSTRUM           spect
PROBHD   5 mm DUL 13C-1
PULPROG            zg30
TD                32768
SOLVENT            DMSO
NS                    7
DS                    0
SWH            6410.256 Hz
FIDRES         0.195625 Hz
AQ            2.5559540 sec
RG                  181
DW               78.000 usec
DE                 6.00 usec
TE                300.0 K
D1           2.00000000 sec
TD0                   1

======== CHANNEL f1 ========
NUC1                 1H
P1                10.00 usec
PL1               -2.40 dB
SFO1        400.1528010 MHz

F2 - Processing parameters
SI                16384
SF          400.1500027 MHz
WDW                  EM
SSB      0
LB       0 Hz
GB       0
PC                 1.00
```

S1

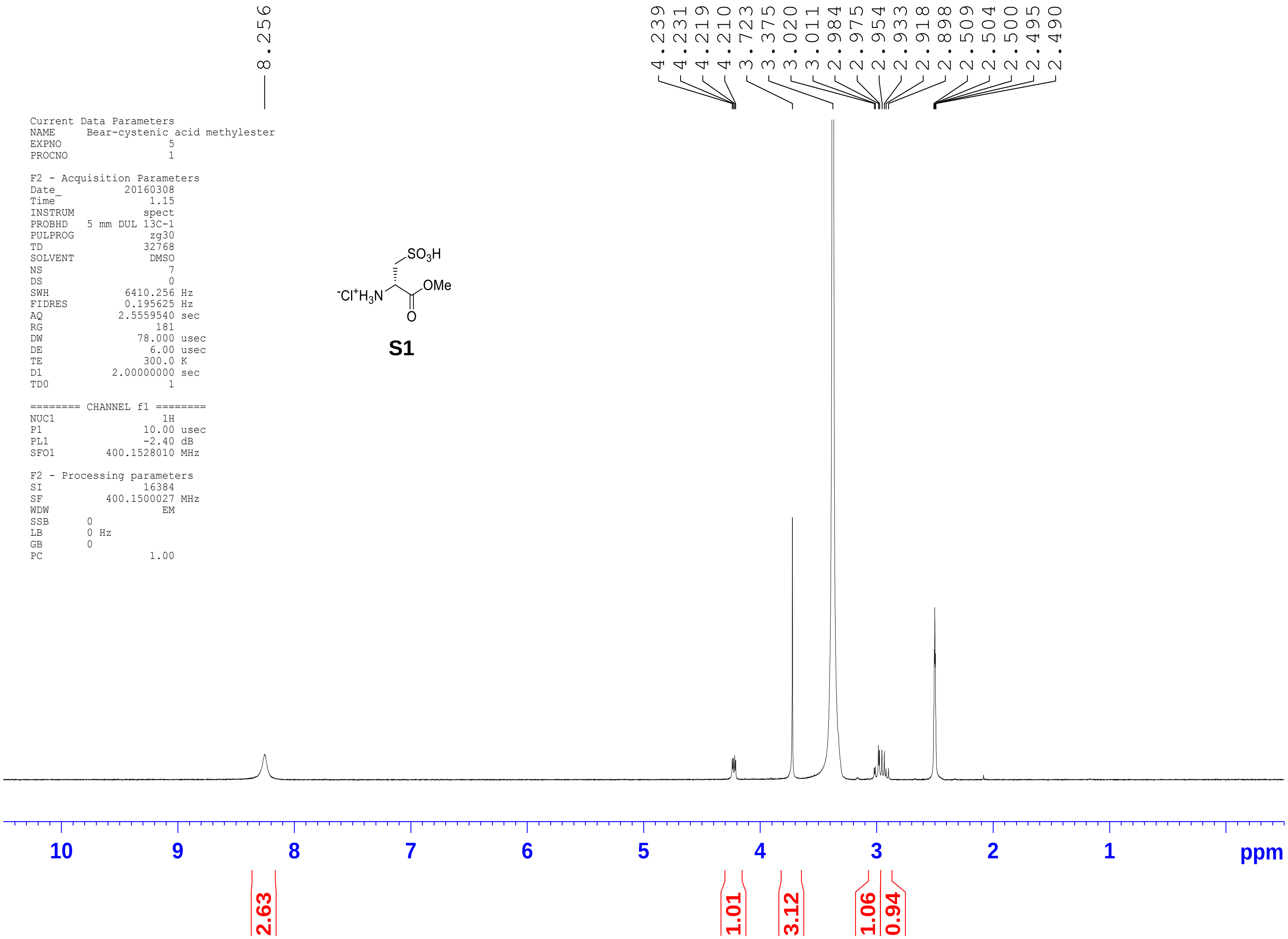

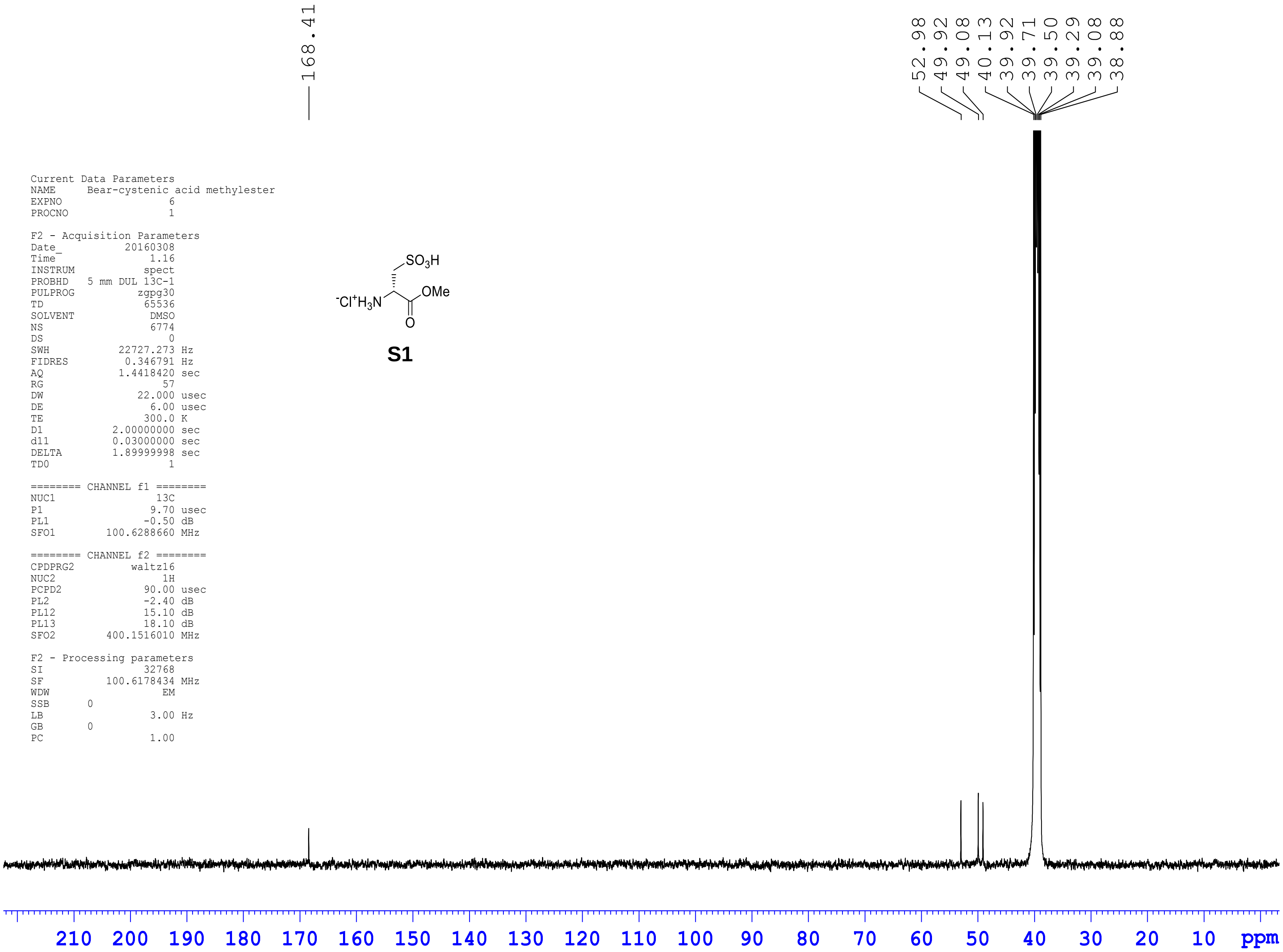
168.41
52.98
49.92
49.08
40.13
39.92
39.71
39.50
39.29
39.08
38.88
Current Data Parameters
NAME Bear-cystenic acid methylester
EXPNO 6
PROCNO 1
F2 - Acquisition Parameters
Date_ 20160308
Time 1.16
INSTRUM spect
PROBHD 5 mm DUL 13C-1
PULPROG zgpg30
TD 65536
SOLVENT DMSO
NS 6774
DS 0
SWH 22727.273 Hz
FIDRES 0.346791 Hz
AQ 1.4418420 sec
RG 57
DW 22.000 usec
DE 6.00 usec
TE 300.0 K
D1 2.00000000 sec
d11 0.03000000 sec
DELTA 1.89999998 sec
TD0 1
======== CHANNEL f1 ========
NUC1 13C
P1 9.70 usec
PL1 -0.50 dB
SFO1 100.6288660 MHz
======== CHANNEL f2 ========
CPDPRG2 waltz16
NUC2 1H
PCPD2 90.00 usec
PL2 -2.40 dB
PL12 15.10 dB
PL13 18.10 dB
SFO2 400.1516010 MHz
F2 - Processing parameters
SI 32768
SF 100.6178434 MHz
WDW EM
SSB 0
LB 3.00 Hz
GB 0
PC 1.00
SO3H
OMe
-Cl+H3N
O
S1
210 200 190 180 170 160 150 140 130 120 110 100 90 80 70 60 50 40 30 20 10 ppm

Current Data Parameters
NAME Bear-Boc-Cysteic acid -20160516
EXPNO 1
PROCNO 1

F2 - Acquisition Parameters
Date_ 20160516
Time 14.08
INSTRUM spect
PROBHD 5 mm DUL 13C-1
PULPROG zg30
TD 32768
SOLVENT MeOD
NS 13
DS 0
SWH 6410.256 Hz
FIDRES 0.195625 Hz
AQ 2.5559540 sec
RG 456
DW 78.000 usec
DE 6.00 usec
TE 300.0 K
D1 2.00000000 sec
TD0 1

======== CHANNEL f1 ========
NUC1 1H
P1 10.00 usec
PL1 -2.40 dB
SFO1 400.1528010 MHz

F2 - Processing parameters
SI 16384
SF 400.1500068 MHz
WDW EM
SSB 0
LB 0 Hz
GB 0
PC 1.00

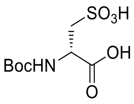

11

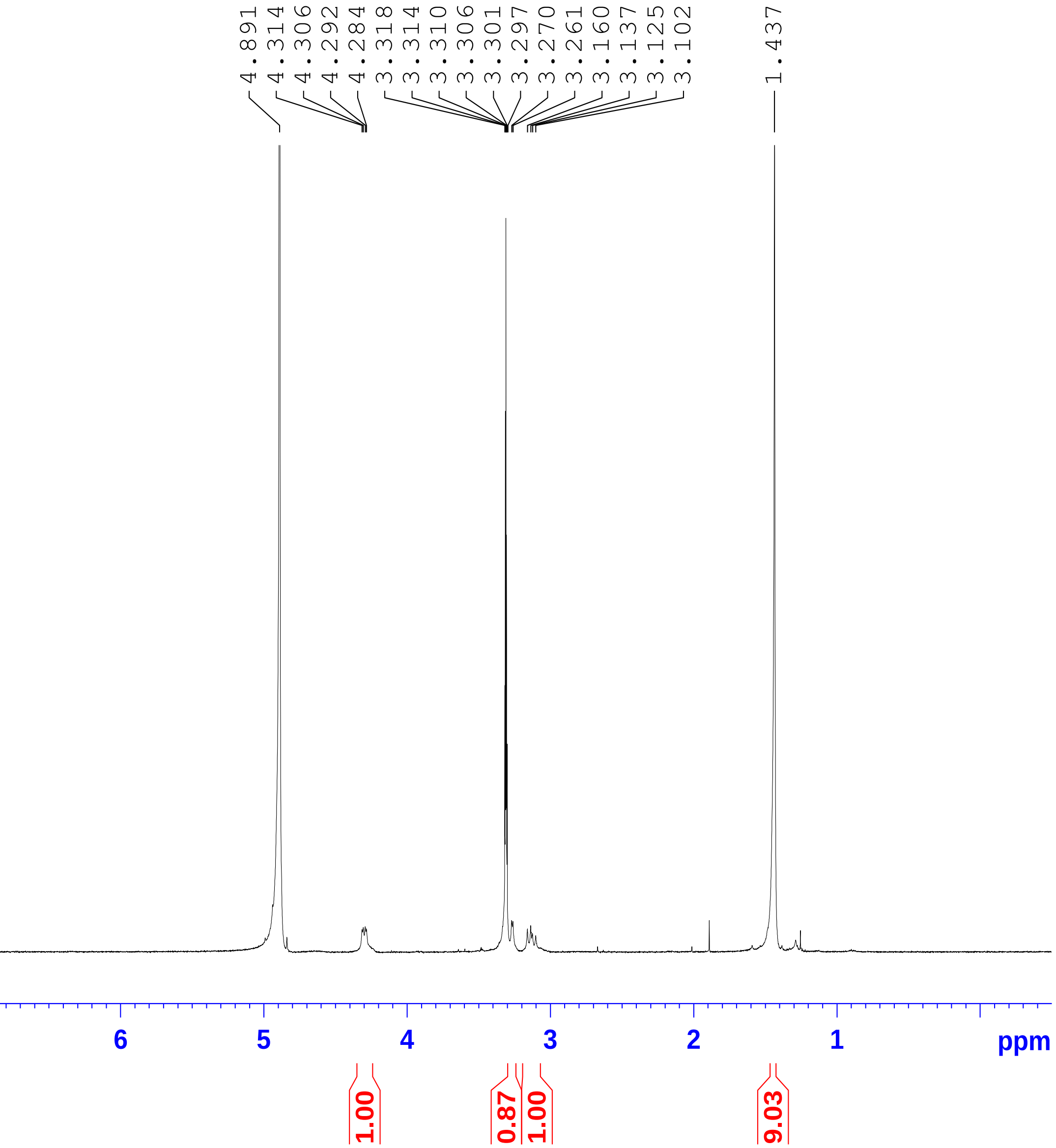

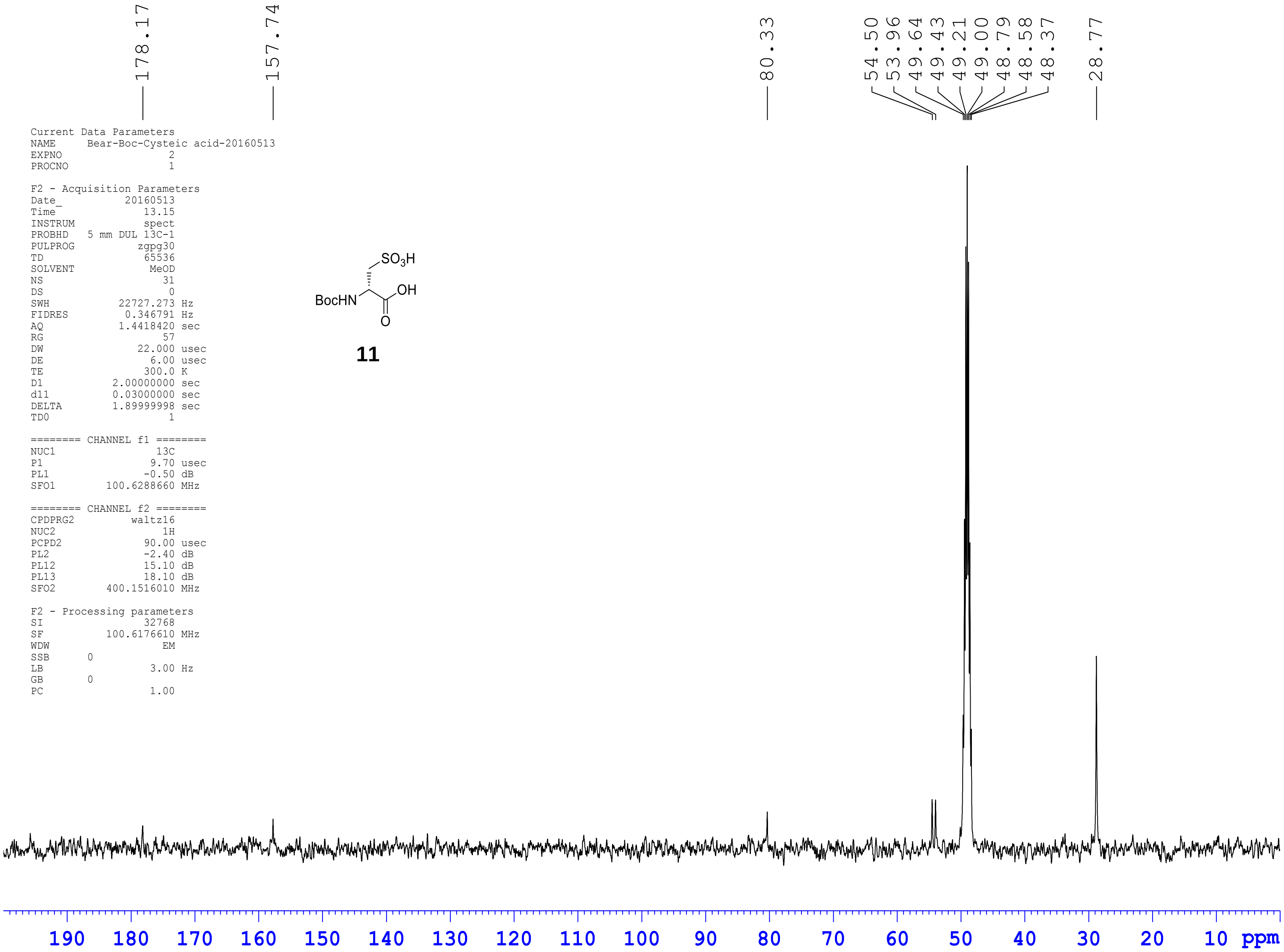

11

```
Current Data Parameters
NAME     Bear-Boc-Cysteic acid-20160513
EXPNO                 2
PROCNO                1

F2 - Acquisition Parameters
Date_          20160513
Time              13.15
INSTRUM           spect
PROBHD   5 mm DUL 13C-1
PULPROG          zgpg30
TD                65536
SOLVENT            MeOD
NS                   31
DS                    0
SWH           22727.273 Hz
FIDRES         0.346791 Hz
AQ            1.4418420 sec
RG                   57
DW               22.000 usec
DE                 6.00 usec
TE                300.0 K
D1           2.00000000 sec
d11          0.03000000 sec
DELTA        1.89999998 sec
TD0                   1

======== CHANNEL f1 ========
NUC1                13C
P1                 9.70 usec
PL1               -0.50 dB
SFO1        100.6288660 MHz

======== CHANNEL f2 ========
CPDPRG2         waltz16
NUC2                 1H
PCPD2             90.00 usec
PL2               -2.40 dB
PL12              15.10 dB
PL13              18.10 dB
SFO2        400.1516010 MHz

F2 - Processing parameters
SI                32768
SF          100.6176610 MHz
WDW                  EM
SSB      0
LB                 3.00 Hz
GB       0
PC                 1.00
```

```
Current Data Parameters
NAME            AA-26-2-1H
EXPNO                    1
PROCNO                   1

F2 - Acquisition Parameters
Date_             20111211
Time                 10.52
INSTRUM              spect
PROBHD   5 mm DUL 13C-1
PULPROG               zg30
TD                   32768
SOLVENT               MeOD
NS                      13
DS                       0
SWH               6410.256 Hz
FIDRES            0.195625 Hz
AQ               2.5559540 sec
RG                      64
DW                  78.000 usec
DE                    6.00 usec
TE                   300.0 K
D1              2.00000000 sec
TD0                      1

======== CHANNEL f1 ========
NUC1                    1H
P1                   10.00 usec
PL1                  -2.40 dB
SFO1           400.1528010 MHz

F2 - Processing parameters
SI                   16384
SF             400.1500070 MHz
WDW                     EM
SSB      0
LB       0 Hz
GB       0
PC                    1.00
```

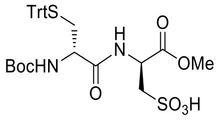

S2

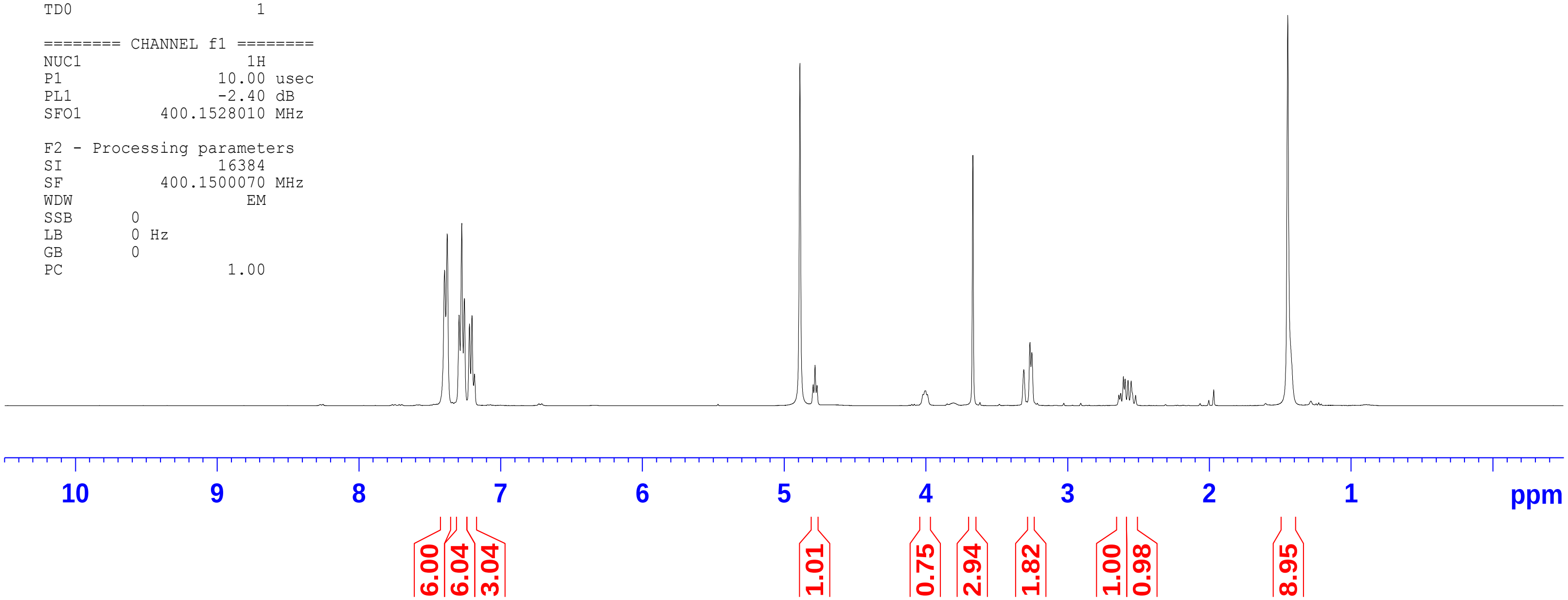

```
Current Data Parameters
NAME         AA-26-2-13C
EXPNO                  1
PROCNO                 1

F2 - Acquisition Parameters
Date_           20111211
Time               10.58
INSTRUM            spect
PROBHD   5 mm DUL 13C-1
PULPROG          zgpg30
TD                65536
SOLVENT             MeOD
NS                   338
DS                     0
SWH            22727.273 Hz
FIDRES          0.346791 Hz
AQ             1.4418420 sec
RG                   228
DW                22.000 usec
DE                  6.00 usec
TE                 300.0 K
D1            2.00000000 sec
d11           0.03000000 sec
DELTA         1.89999998 sec
TD0                    1

======== CHANNEL f1 ========
NUC1                 13C
P1                  9.70 usec
PL1                -0.50 dB
SFO1         100.6288660 MHz

======== CHANNEL f2 ========
CPDPRG2          waltz16
NUC2                  1H
PCPD2              90.00 usec
PL2                -2.40 dB
PL12               15.10 dB
PL13               18.10 dB
SFO2         400.1516010 MHz

F2 - Processing parameters
SI                 32768
SF           100.6176662 MHz
WDW                   EM
SSB                    0
LB                  3.00 Hz
GB                     0
PC                  1.00
```

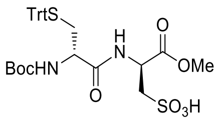

S2

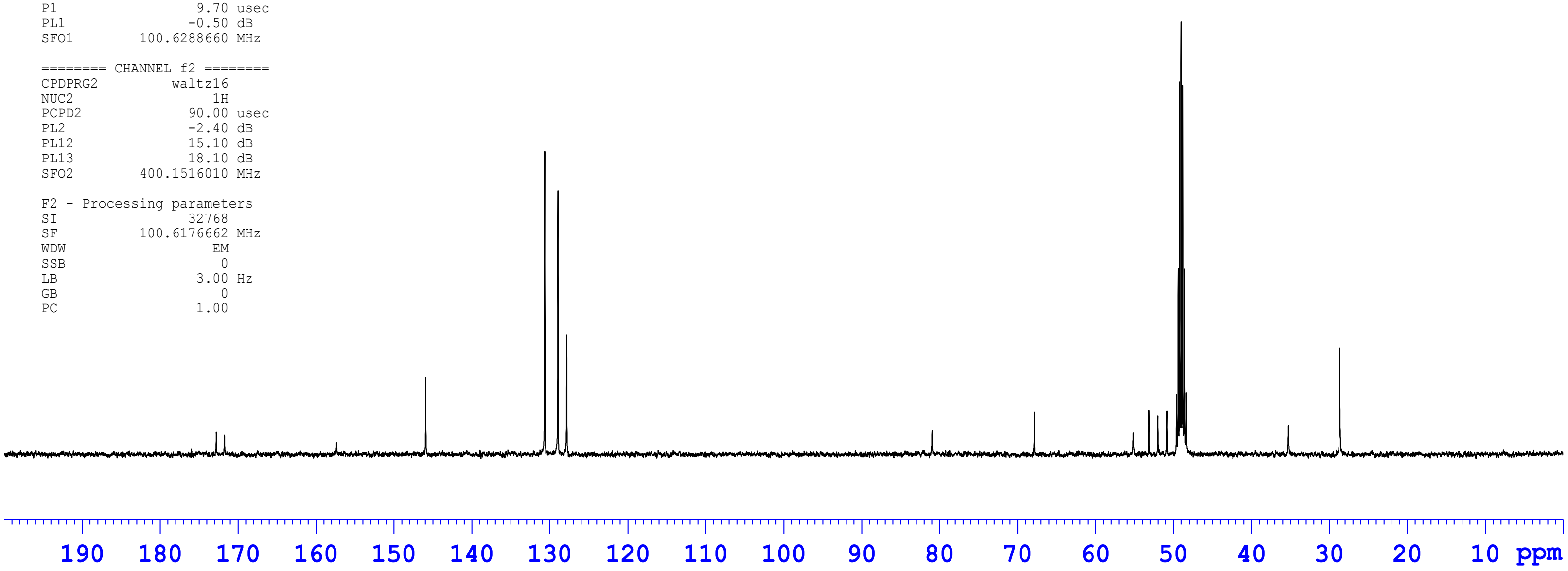

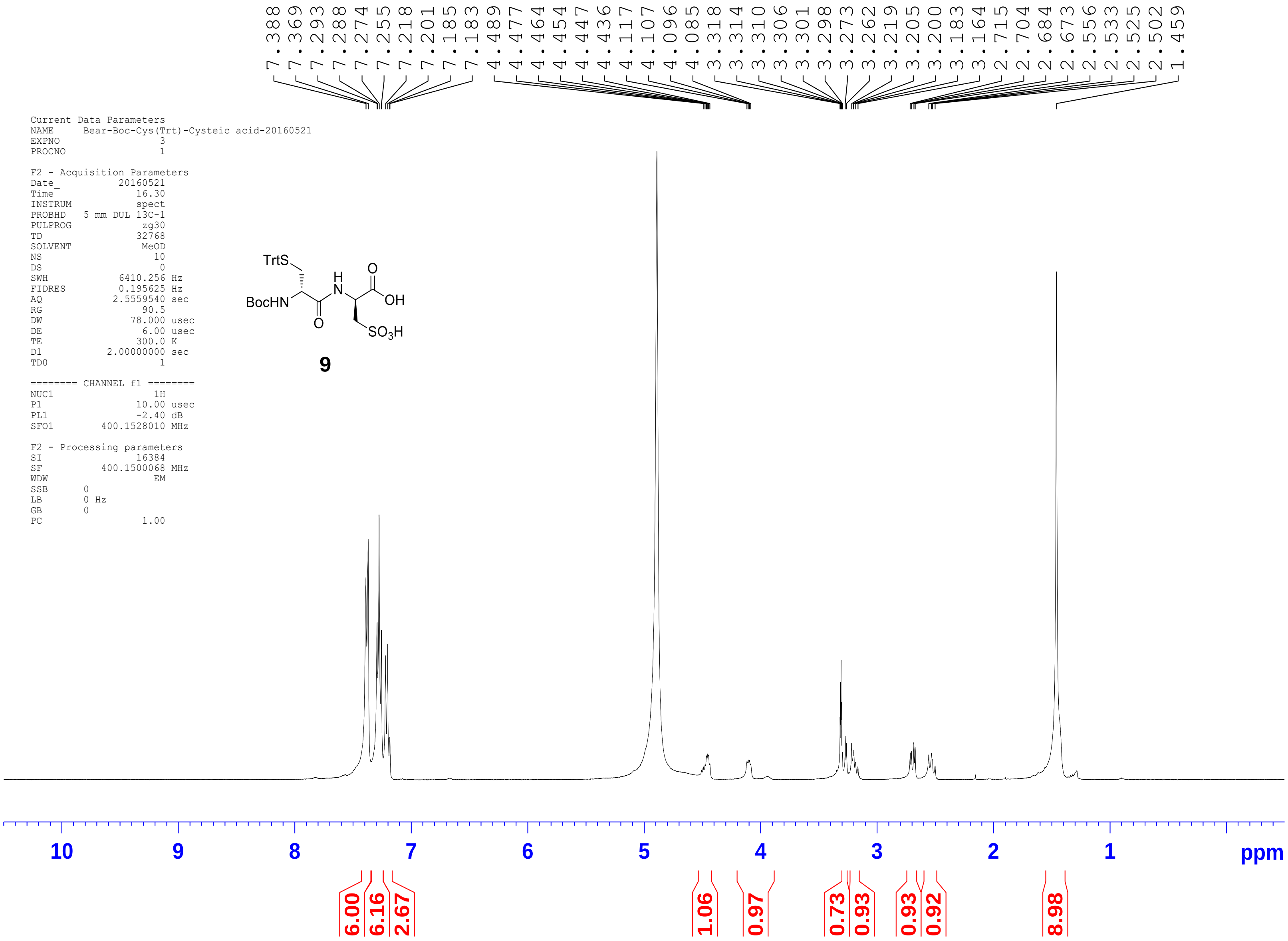

7.388
7.369
7.293
7.288
7.274
7.255
7.218
7.201
7.185
7.183
4.489
4.477
4.464
4.454
4.447
4.436
4.117
4.107
4.096
4.085
3.318
3.314
3.310
3.306
3.301
3.298
3.273
3.262
3.219
3.205
3.200
3.183
3.164
2.715
2.704
2.684
2.673
2.556
2.533
2.525
2.502
1.459
Current Data Parameters
NAME Bear-Boc-Cys(Trt)-Cysteic acid-20160521
EXPNO 3
PROCNO 1
F2 - Acquisition Parameters
Date_ 20160521
Time 16.30
INSTRUM spect
PROBHD 5 mm DUL 13C-1
PULPROG zg30
TD 32768
SOLVENT MeOD
NS 10
DS 0
SWH 6410.256 Hz
FIDRES 0.195625 Hz
AQ 2.5559540 sec
RG 90.5
DW 78.000 usec
DE 6.00 usec
TE 300.0 K
D1 2.00000000 sec
TD0 1
======== CHANNEL f1 ========
NUC1 1H
P1 10.00 usec
PL1 -2.40 dB
SFO1 400.1528010 MHz
F2 - Processing parameters
SI 16384
SF 400.1500068 MHz
WDW EM
SSB 0
LB 0 Hz
GB 0
PC 1.00
TrtS
BocHN
H
N
O
OH
O
SO3H
9
10
9
8
7
6
5
4
3
2
1
ppm
6.00
6.16
2.67
1.06
0.97
0.73
0.93
0.93
0.92
8.98

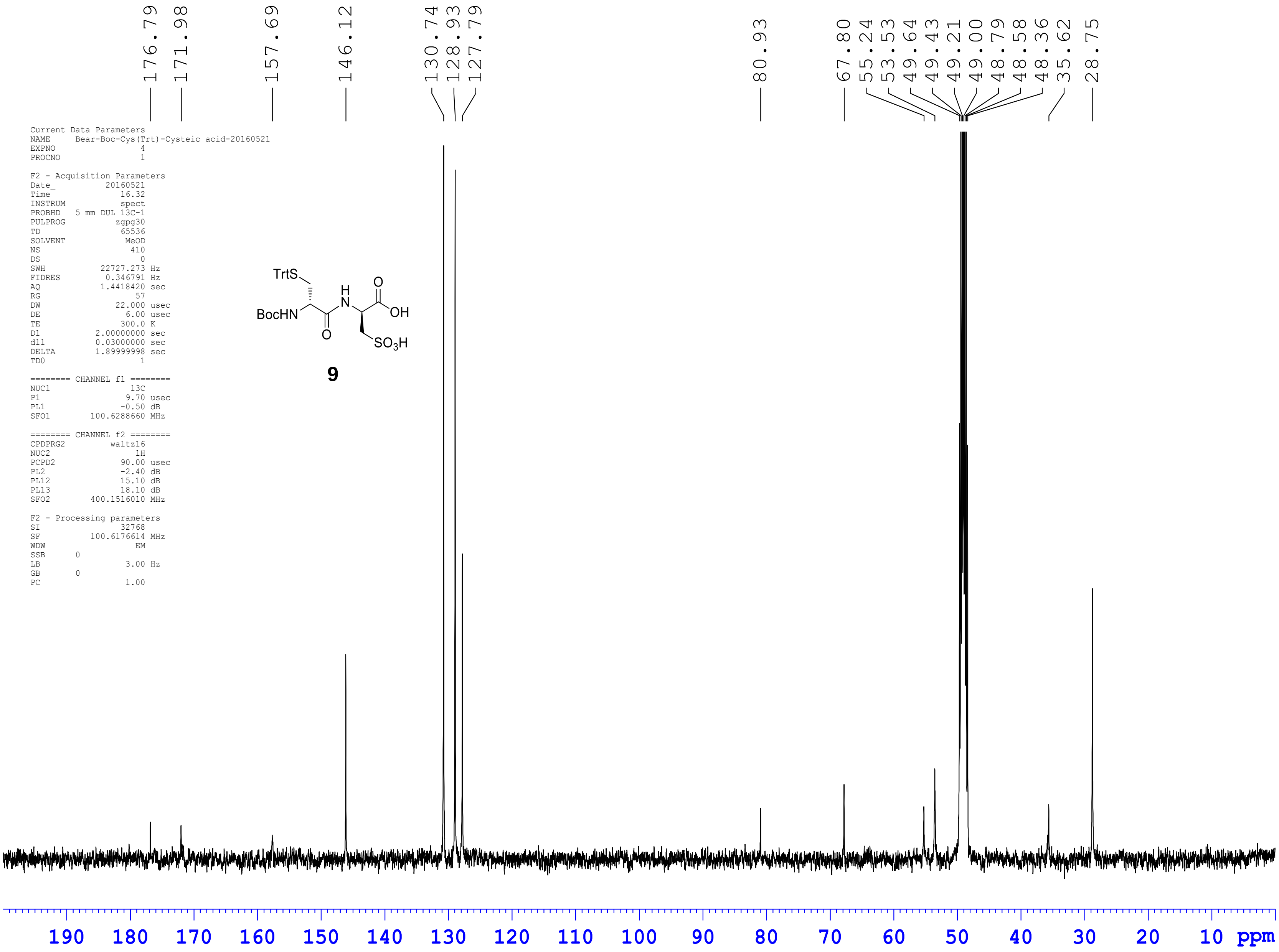
176.79
171.98
157.69
146.12
130.74
128.93
127.79
80.93
67.80
55.24
53.53
49.64
49.43
49.21
49.00
48.79
48.58
48.36
35.62
28.75
Current Data Parameters
NAME Bear-Boc-Cys(Trt)-Cysteic acid-20160521
EXPNO 4
PROCNO 1
F2 - Acquisition Parameters
Date_ 20160521
Time 16.32
INSTRUM spect
PROBHD 5 mm DUL 13C-1
PULPROG zgpg30
TD 65536
SOLVENT MeOD
NS 410
DS 0
SWH 22727.273 Hz
FIDRES 0.346791 Hz
AQ 1.4418420 sec
RG 57
DW 22.000 usec
DE 6.00 usec
TE 300.0 K
D1 2.00000000 sec
d11 0.03000000 sec
DELTA 1.89999998 sec
TD0 1
======== CHANNEL f1 ========
NUC1 13C
P1 9.70 usec
PL1 -0.50 dB
SFO1 100.6288660 MHz
======== CHANNEL f2 ========
CPDPRG2 waltz16
NUC2 1H
PCPD2 90.00 usec
PL2 -2.40 dB
PL12 15.10 dB
PL13 18.10 dB
SFO2 400.1516010 MHz
F2 - Processing parameters
SI 32768
SF 100.6176614 MHz
WDW EM
SSB 0
LB 3.00 Hz
GB 0
PC 1.00
TrtS
BocHN
H
N
O
OH
O
SO3H
9
190 180 170 160 150 140 130 120 110 100 90 80 70 60 50 40 30 20 10 ppm

Current Data Parameters
NAME Rf8-COOH
EXPNO 1
PROCNO 1

F2 - Acquisition Parameters
Date_ 20160309
Time 17.52
INSTRUM spect
PROBHD 5 mm DUL 13C-1
PULPROG zg30
TD 32768
SOLVENT DMSO
NS 10
DS 0
SWH 6410.256 Hz
FIDRES 0.195625 Hz
AQ 2.5559540 sec
RG 228
DW 78.000 usec
DE 6.00 usec
TE 300.0 K
D1 2.00000000 sec
TD0 1

======== CHANNEL f1 ========
NUC1 1H
P1 10.00 usec
PL1 -2.40 dB
SFO1 400.1528010 MHz

F2 - Processing parameters
SI 16384
SF 400.1500027 MHz
WDW EM
SSB 0
LB 0 Hz
GB 0
PC 1.00

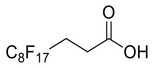

6

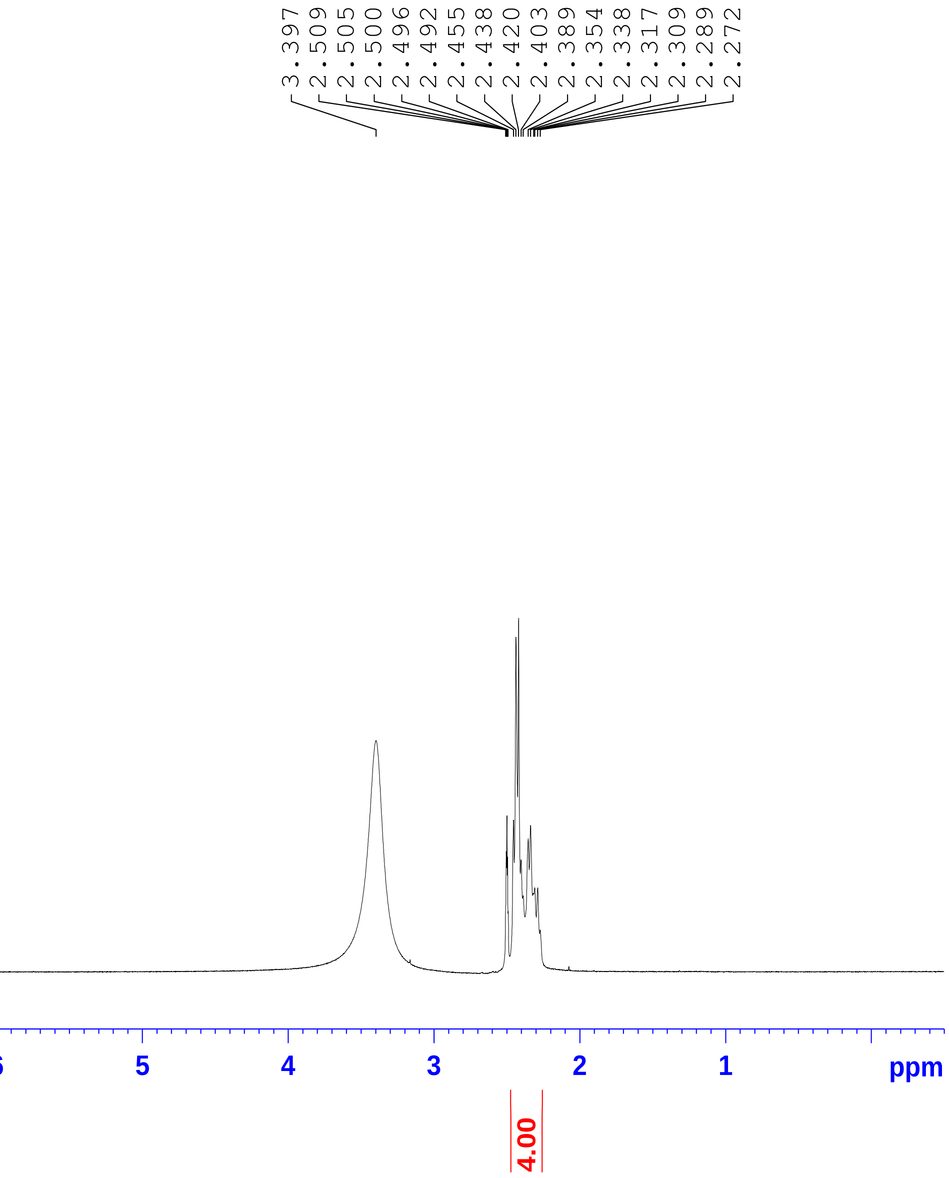

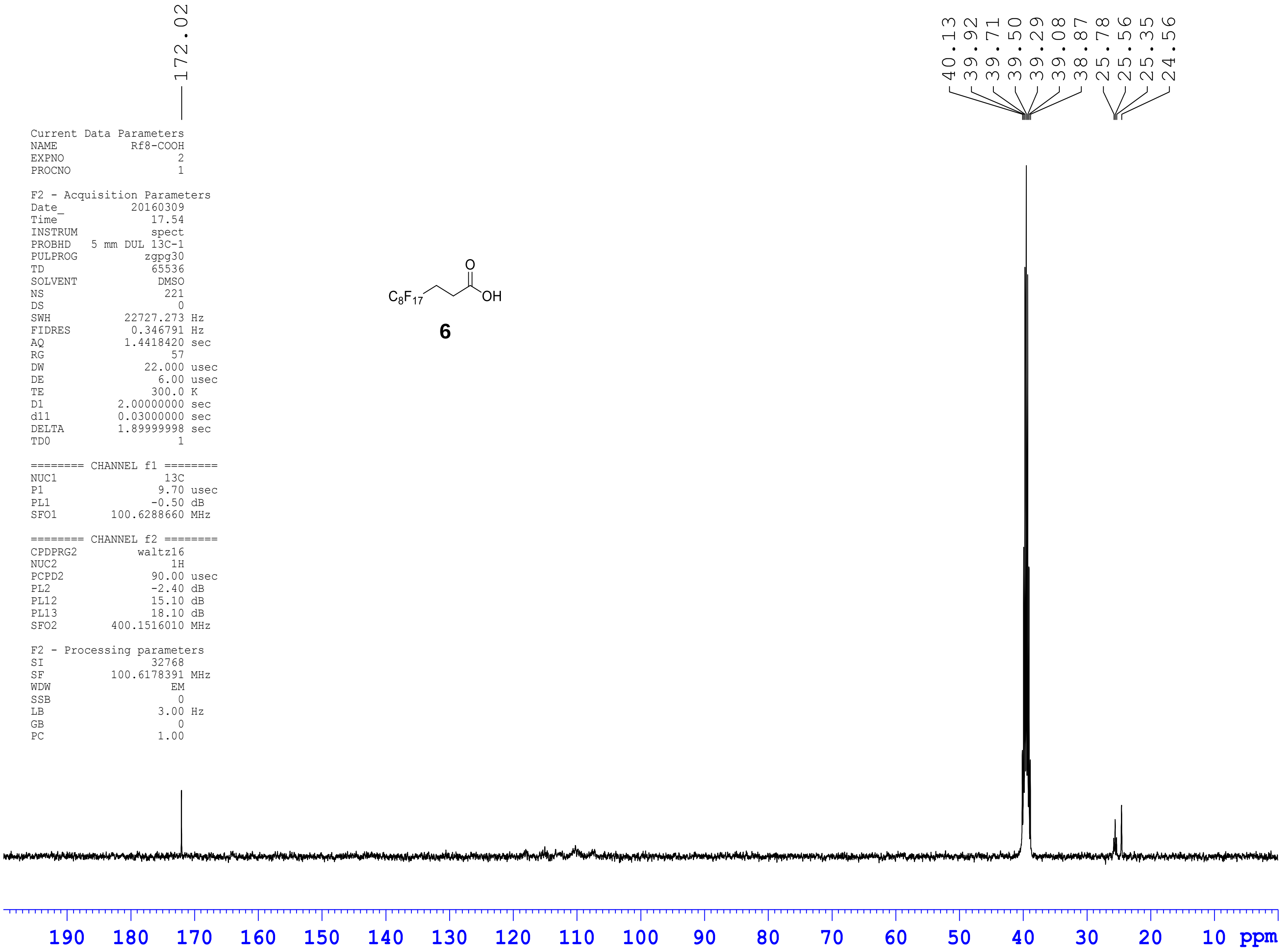

Current Data Parameters
NAME Rf8-COOH
EXPNO 2
PROCNO 1

F2 - Acquisition Parameters
Date_ 20160309
Time 17.54
INSTRUM spect
PROBHD 5 mm DUL 13C-1
PULPROG zgpg30
TD 65536
SOLVENT DMSO
NS 221
DS 0
SWH 22727.273 Hz
FIDRES 0.346791 Hz
AQ 1.4418420 sec
RG 57
DW 22.000 usec
DE 6.00 usec
TE 300.0 K
D1 2.00000000 sec
d11 0.03000000 sec
DELTA 1.89999998 sec
TD0 1

======== CHANNEL f1 ========
NUC1 13C
P1 9.70 usec
PL1 -0.50 dB
SFO1 100.6288660 MHz

======== CHANNEL f2 ========
CPDPRG2 waltz16
NUC2 1H
PCPD2 90.00 usec
PL2 -2.40 dB
PL12 15.10 dB
PL13 18.10 dB
SFO2 400.1516010 MHz

F2 - Processing parameters
SI 32768
SF 100.6178391 MHz
WDW EM
SSB 0
LB 3.00 Hz
GB 0
PC 1.00

6

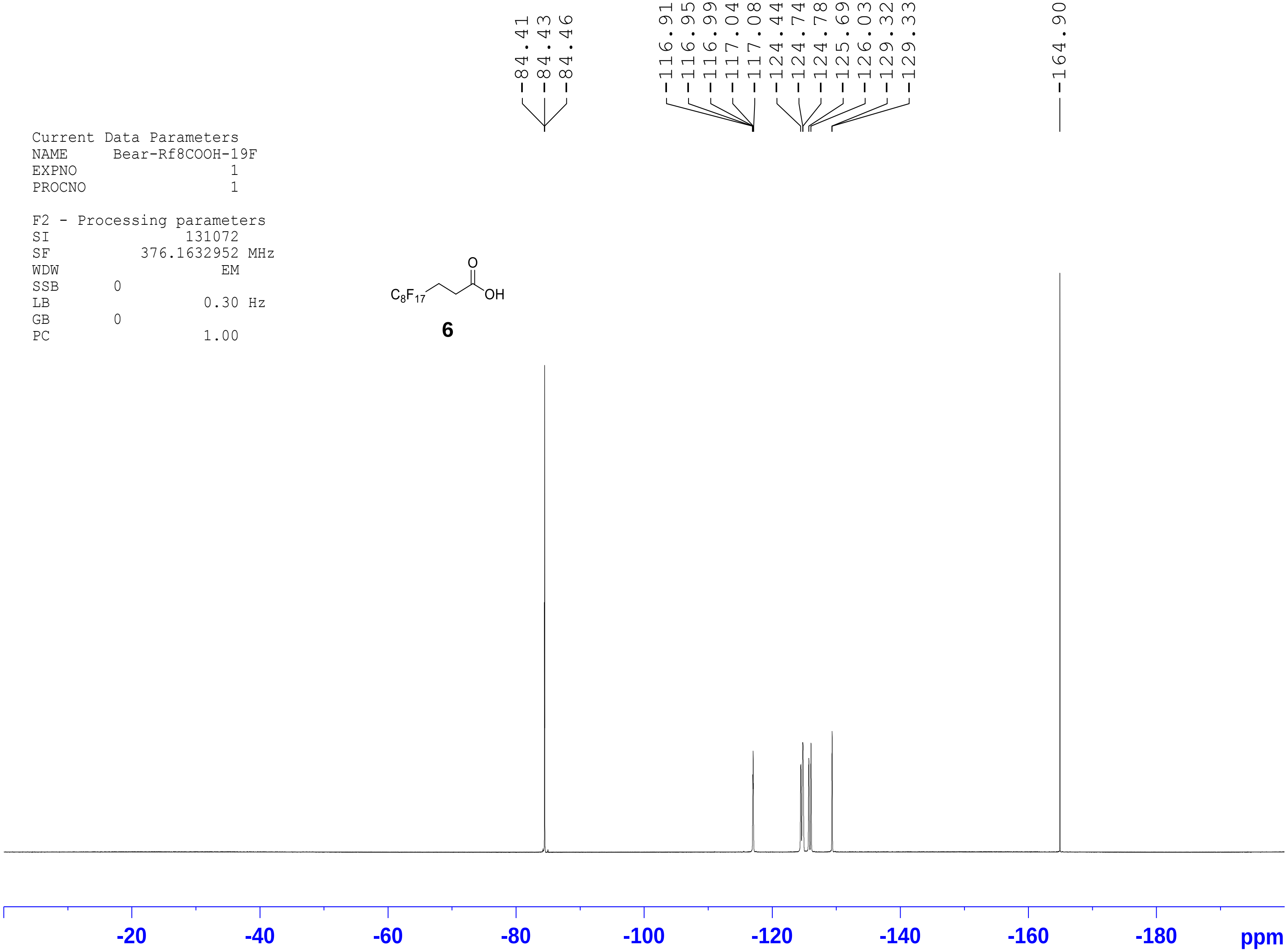
-84.41
-84.43
-84.46
-116.91
-116.95
-116.99
-117.04
-117.08
-124.44
-124.74
-124.78
-125.69
-126.03
-129.32
-129.33
-164.90
Current Data Parameters
NAME Bear-Rf8COOH-19F
EXPNO 1
PROCNO 1
F2 - Processing parameters
SI 131072
SF 376.1632952 MHz
WDW EM
SSB 0
LB 0.30 Hz
GB 0
PC 1.00
-20
-40
-60
-80
-100
-120
-140
-160
-180
ppm
O
C_8F_{17}
OH

6

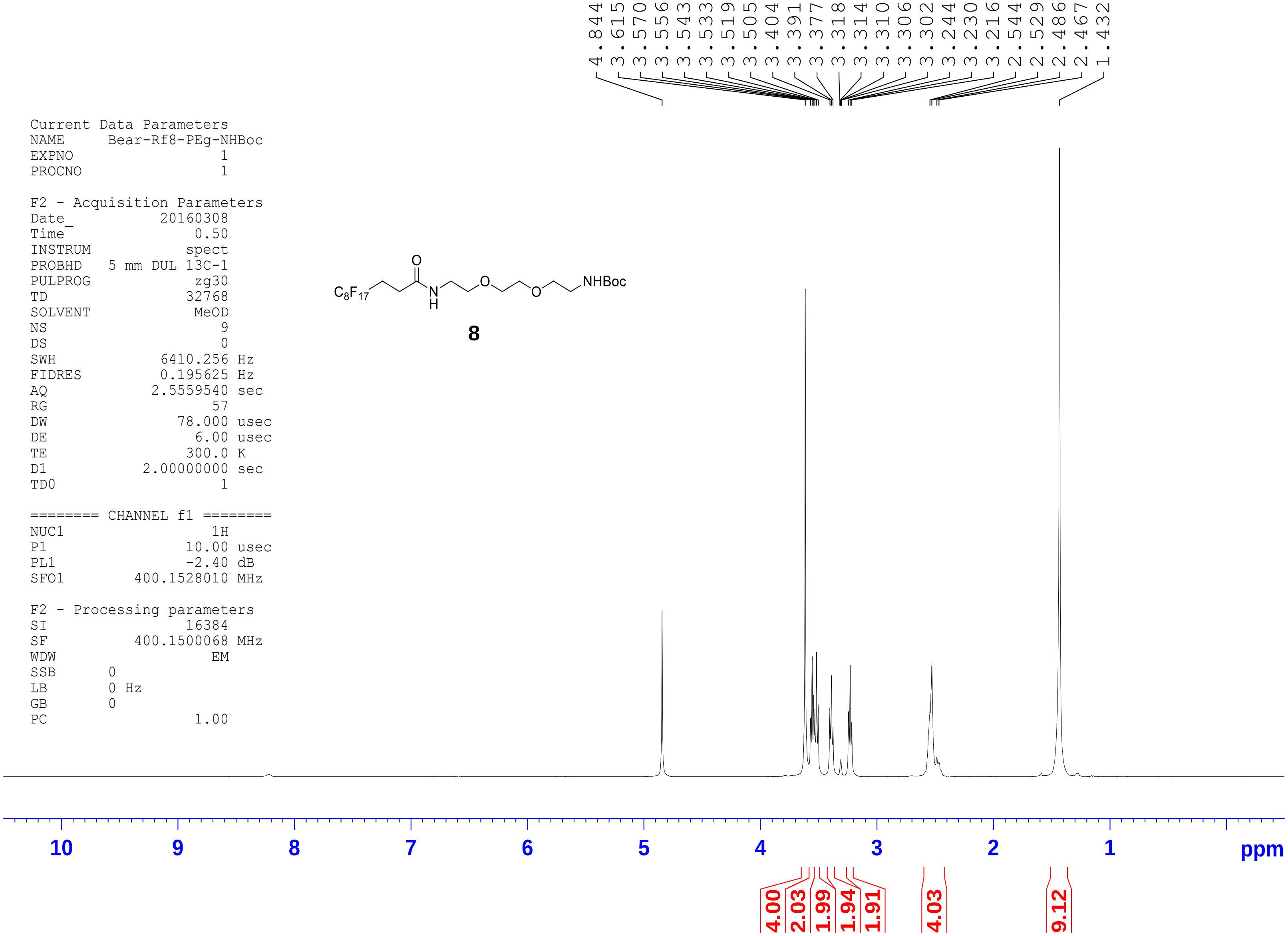

Current Data Parameters
NAME Bear-Rf8-PEg-NHBoc
EXPNO 1
PROCNO 1
F2 - Acquisition Parameters
Date_ 20160308
Time 0.50
INSTRUM spect
PROBHD 5 mm DUL 13C-1
PULPROG zg30
TD 32768
SOLVENT MeOD
NS 9
DS 0
SWH 6410.256 Hz
FIDRES 0.195625 Hz
AQ 2.5559540 sec
RG 57
DW 78.000 usec
DE 6.00 usec
TE 300.0 K
D1 2.00000000 sec
TD0 1
======== CHANNEL f1 ========
NUC1 1H
P1 10.00 usec
PL1 -2.40 dB
SFO1 400.1528010 MHz
F2 - Processing parameters
SI 16384
SF 400.1500068 MHz
WDW EM
SSB 0
LB 0 Hz
GB 0
PC 1.00
4.844
3.615
3.570
3.556
3.543
3.533
3.519
3.505
3.404
3.391
3.377
3.318
3.314
3.310
3.306
3.302
3.244
3.230
3.216
2.544
2.529
2.486
2.467
1.432
10
9
8
7
6
5
4
3
2
1
ppm
4.00
2.03
1.99
1.94
1.91
4.03
9.12
O
C_8F_{17}
N
H
O
O
NHBoc

8

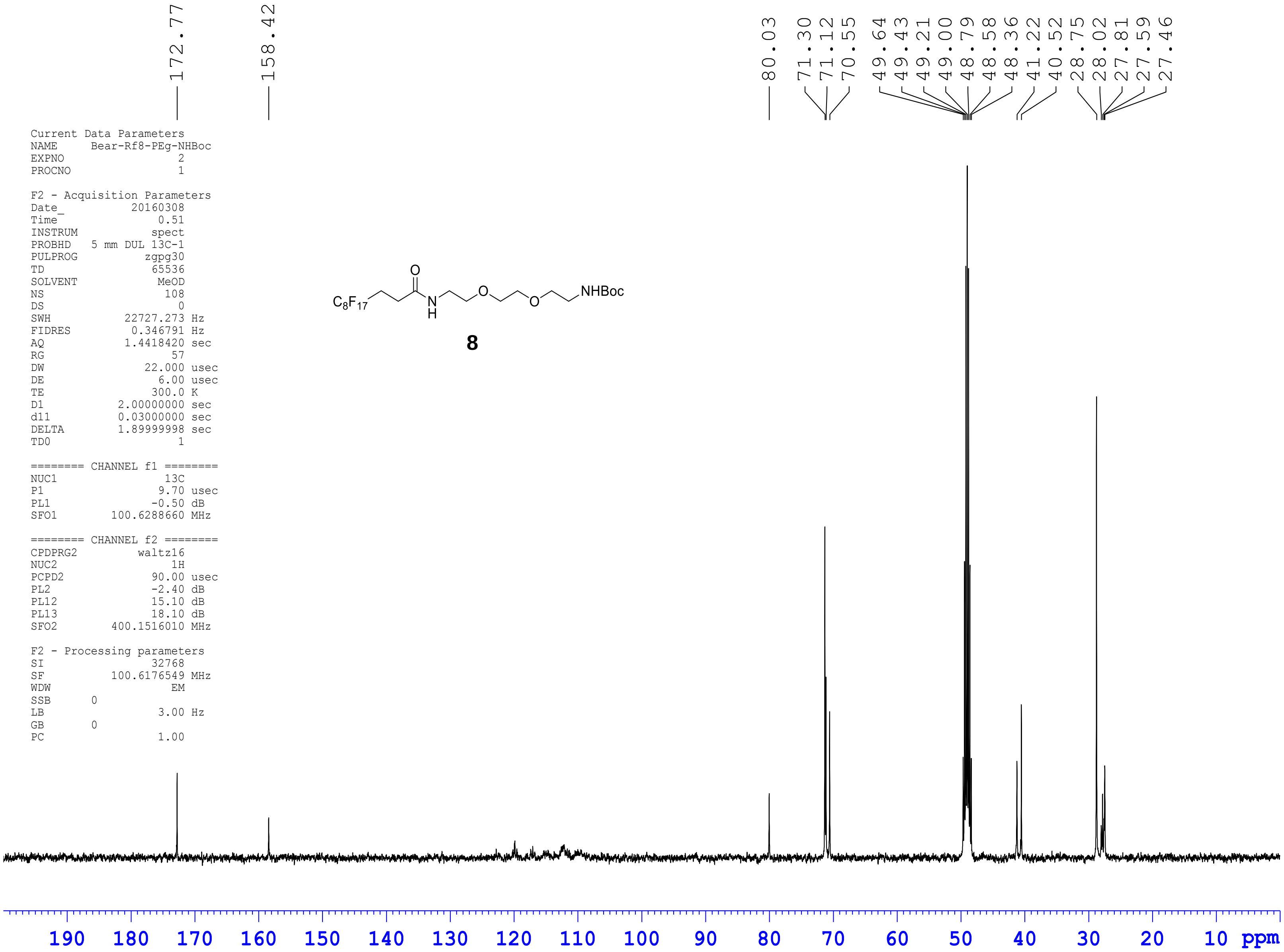
172.77
158.42
80.03
71.30
71.12
70.55
49.64
49.43
49.21
49.00
48.79
48.58
48.36
41.22
40.52
28.75
28.02
27.81
27.59
27.46
Current Data Parameters
NAME Bear-Rf8-PEg-NHBoc
EXPNO 2
PROCNO 1
F2 - Acquisition Parameters
Date_ 20160308
Time 0.51
INSTRUM spect
PROBHD 5 mm DUL 13C-1
PULPROG zgpg30
TD 65536
SOLVENT MeOD
NS 108
DS 0
SWH 22727.273 Hz
FIDRES 0.346791 Hz
AQ 1.4418420 sec
RG 57
DW 22.000 usec
DE 6.00 usec
TE 300.0 K
D1 2.00000000 sec
d11 0.03000000 sec
DELTA 1.89999998 sec
TD0 1
======== CHANNEL f1 ========
NUC1 13C
P1 9.70 usec
PL1 -0.50 dB
SFO1 100.6288660 MHz
======== CHANNEL f2 ========
CPDPRG2 waltz16
NUC2 1H
PCPD2 90.00 usec
PL2 -2.40 dB
PL12 15.10 dB
PL13 18.10 dB
SFO2 400.1516010 MHz
F2 - Processing parameters
SI 32768
SF 100.6176549 MHz
WDW EM
SSB 0
LB 3.00 Hz
GB 0
PC 1.00
C8F17
O
N
H
O
O
NHBoc
8
190 180 170 160 150 140 130 120 110 100 90 80 70 60 50 40 30 20 10 ppm

```
Current Data Parameters
NAME      Bear-Rf8-PEG-NHBoc-19F
EXPNO                    1
PROCNO                   1

F2 - Processing parameters
SI                 131072
SF           376.1621406 MHz
WDW                     EM
SSB       0
LB                  0.30 Hz
GB        0
PC                  1.00
```

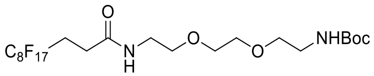

8

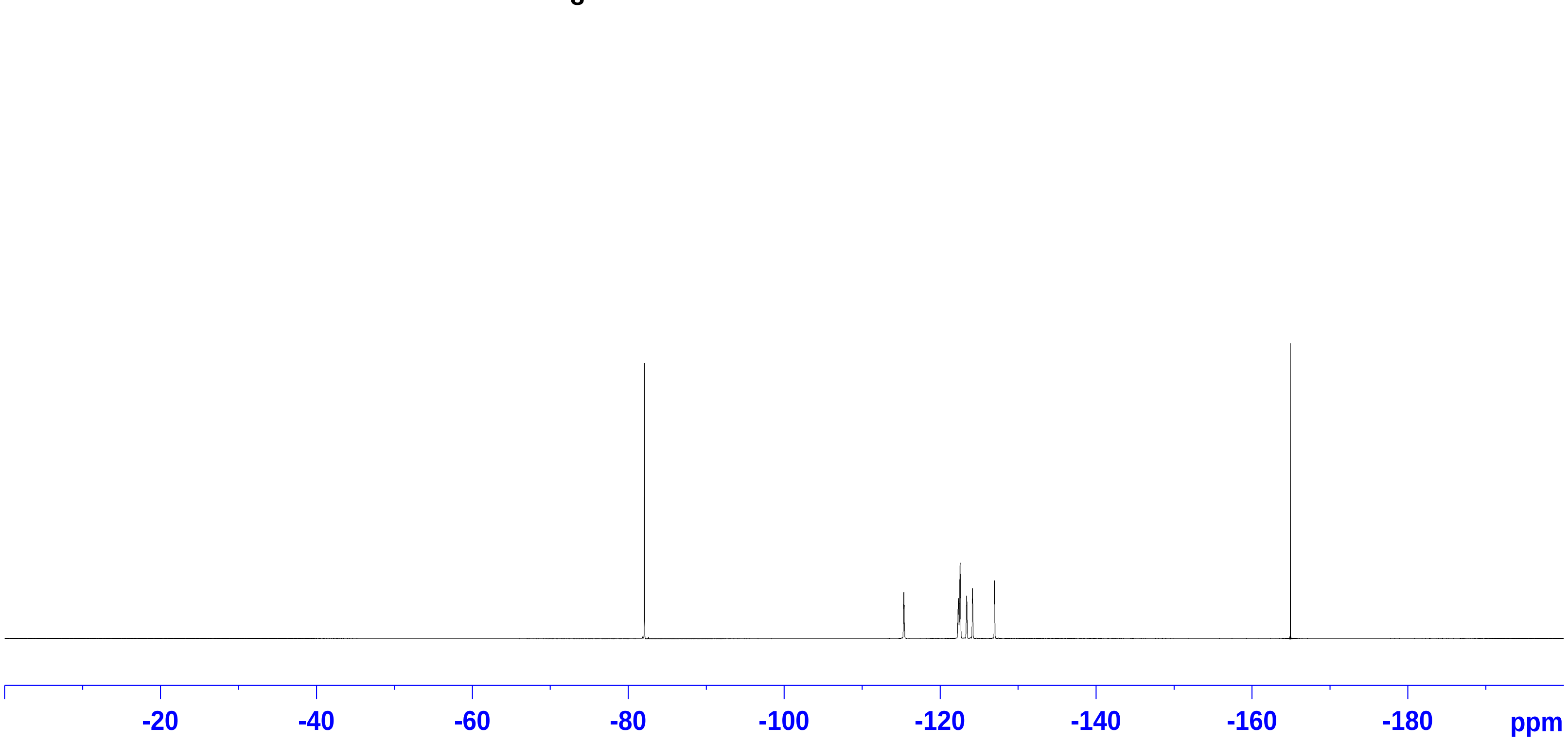

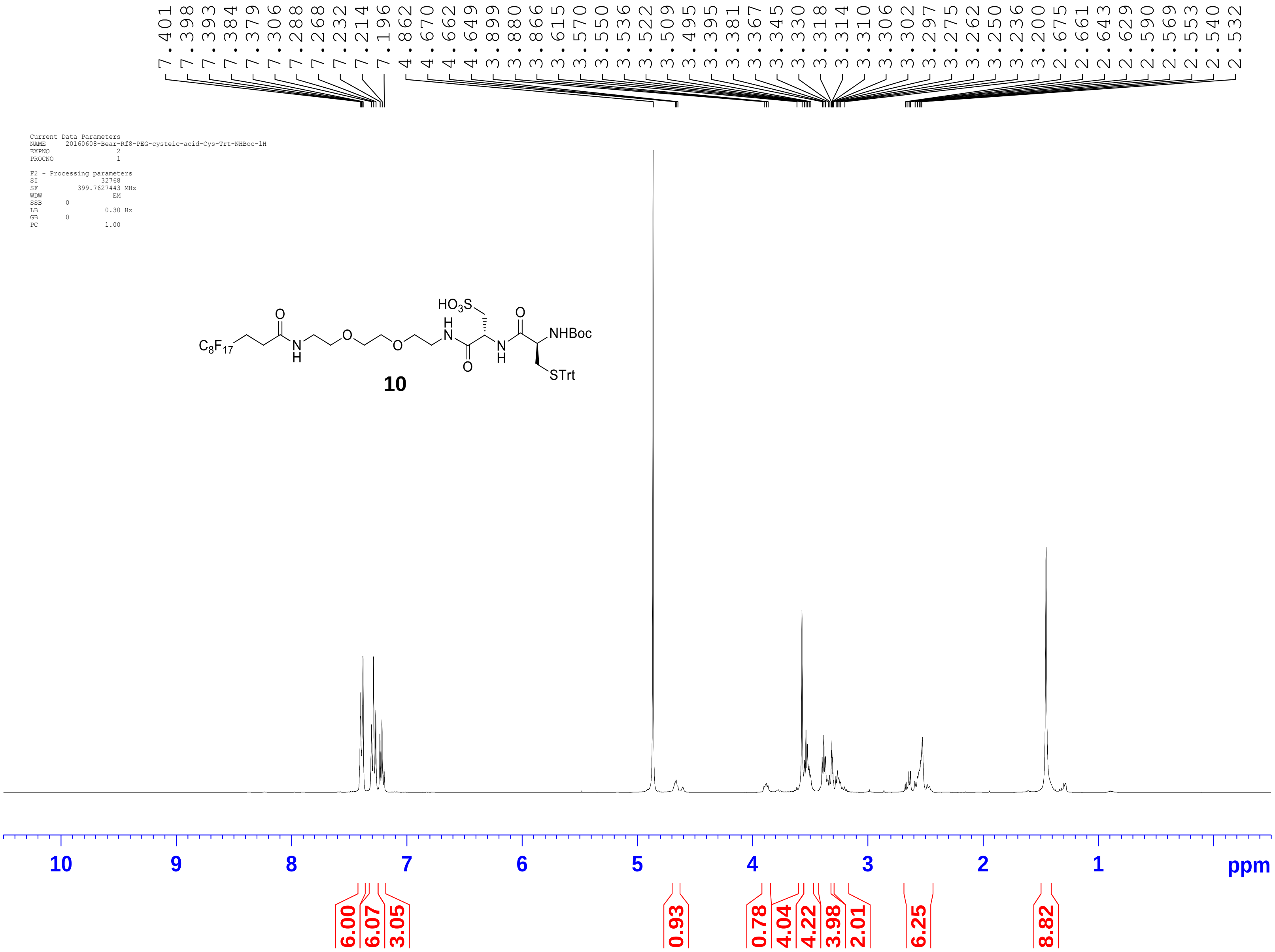
7.401 7.398 7.393 7.384 7.379 7.306 7.288 7.268 7.232 7.214 7.196 4.862 4.670 4.662 4.649 3.899 3.880 3.866 3.615 3.570 3.550 3.536 3.522 3.509 3.495 3.395 3.381 3.367 3.345 3.330 3.318 3.314 3.310 3.306 3.302 3.297 3.275 3.262 3.250 3.236 3.200 2.675 2.661 2.643 2.629 2.590 2.569 2.553 2.540 2.532
Current Data Parameters
NAME 20160608-Bear-Rf8-PEG-cysteic-acid-Cys-Trt-NHBoc-1H
EXPNO 2
PROCNO 1
F2 - Processing parameters
SI 32768
SF 399.7627443 MHz
WDW EM
SSB 0
LB 0.30 Hz
GB 0
PC 1.00
C_8F_{17}
O
N H
HO$_3$S
NHBoc
STrt
10
10 9 8 7 6 5 4 3 2 1 ppm
6.00 6.07 3.05 0.93 0.78 4.04 4.22 3.98 2.01 6.25 8.82

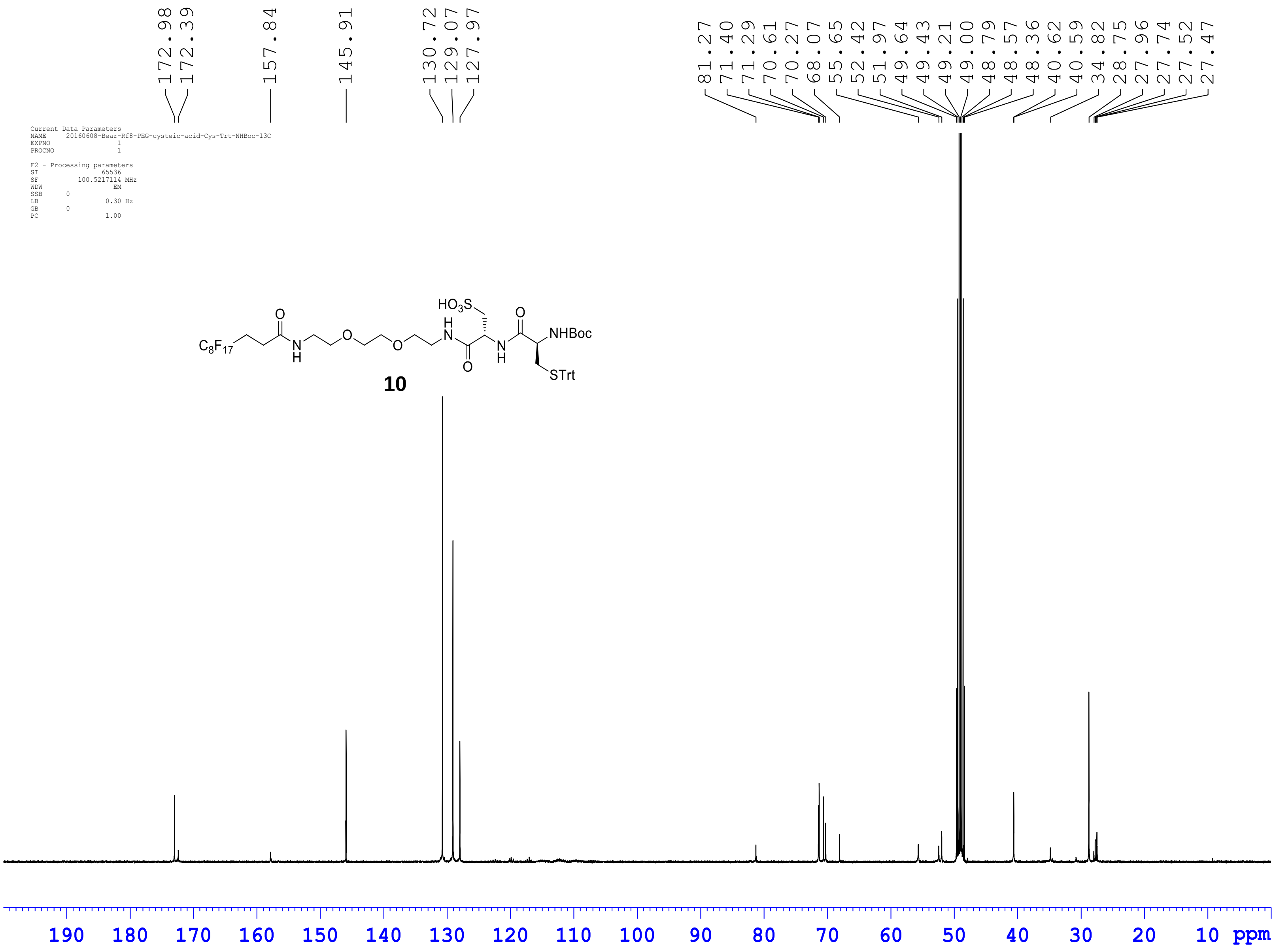

172.98
172.39
157.84
145.91
130.72
129.07
127.97
81.27
71.40
71.29
70.61
70.27
68.07
55.65
52.42
51.97
49.64
49.43
49.21
49.00
48.79
48.57
48.36
40.62
40.59
34.82
28.75
27.96
27.74
27.52
27.47
Current Data Parameters
NAME 20160608-Bear-Rf8-PEG-cysteic-acid-Cys-Trt-NHBoc-13C
EXPNO 1
PROCNO 1
F2 - Processing parameters
SI 65536
SF 100.5217114 MHz
WDW EM
SSB 0
LB 0.30 Hz
GB 0
PC 1.00
HO3S
O
NHBoc
C8F17
N
H
O
O
STrt
10
190 180 170 160 150 140 130 120 110 100 90 80 70 60 50 40 30 20 10 ppm

Current Data Parameters
NAME Bear-Rf8-PEG-cystenic-CysTrtNHBoc-19F
EXPNO 3
PROCNO 1

F2 - Processing parameters
SI 131072
SF 376.1621658 MHz
WDW EM
SSB 0
LB 0.30 Hz
GB 0
PC 1.00

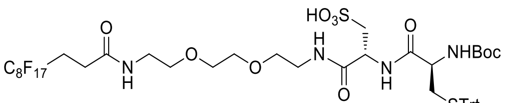

10

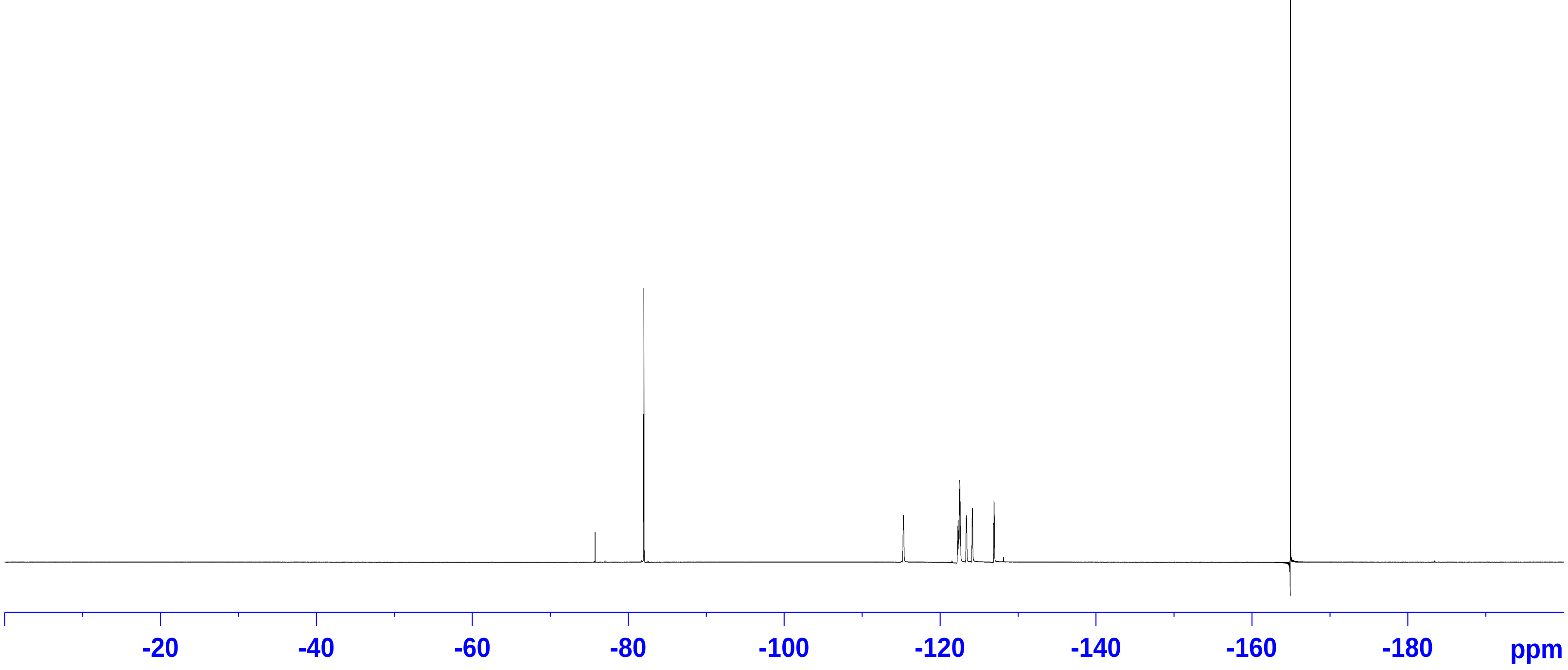

```
Current Data Parameters
NAME       Bear-1074-3-1
EXPNO                  1
PROCNO                 1

F2 - Acquisition Parameters
Date_           20150917
Time                9.35
INSTRUM            spect
PROBHD   5 mm DUL 13C-1
PULPROG             zg30
TD                 32768
SOLVENT             MeOD
NS                    22
DS                     0
SWH             6410.256 Hz
FIDRES          0.195625 Hz
AQ             2.5559540 sec
RG                   322
DW                78.000 usec
DE                  6.00 usec
TE                 300.0 K
D1            2.00000000 sec
TD0                    1

======== CHANNEL f1 ========
NUC1                  1H
P1                 10.00 usec
PL1                -2.40 dB
SFO1         400.1528010 MHz

F2 - Processing parameters
SI                 16384
SF           400.1500069 MHz
WDW                   EM
SSB      0
LB       0 Hz
GB       0
PC                  1.00
```

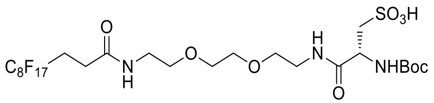

12

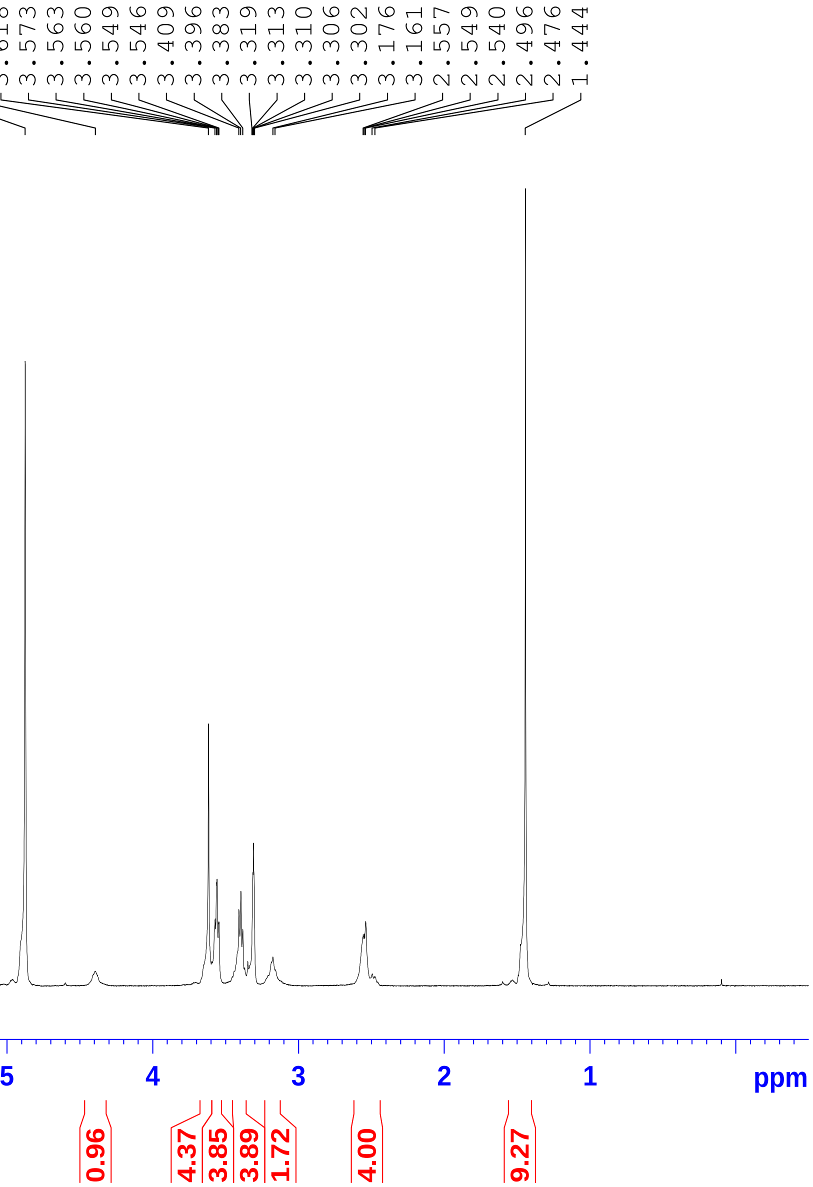

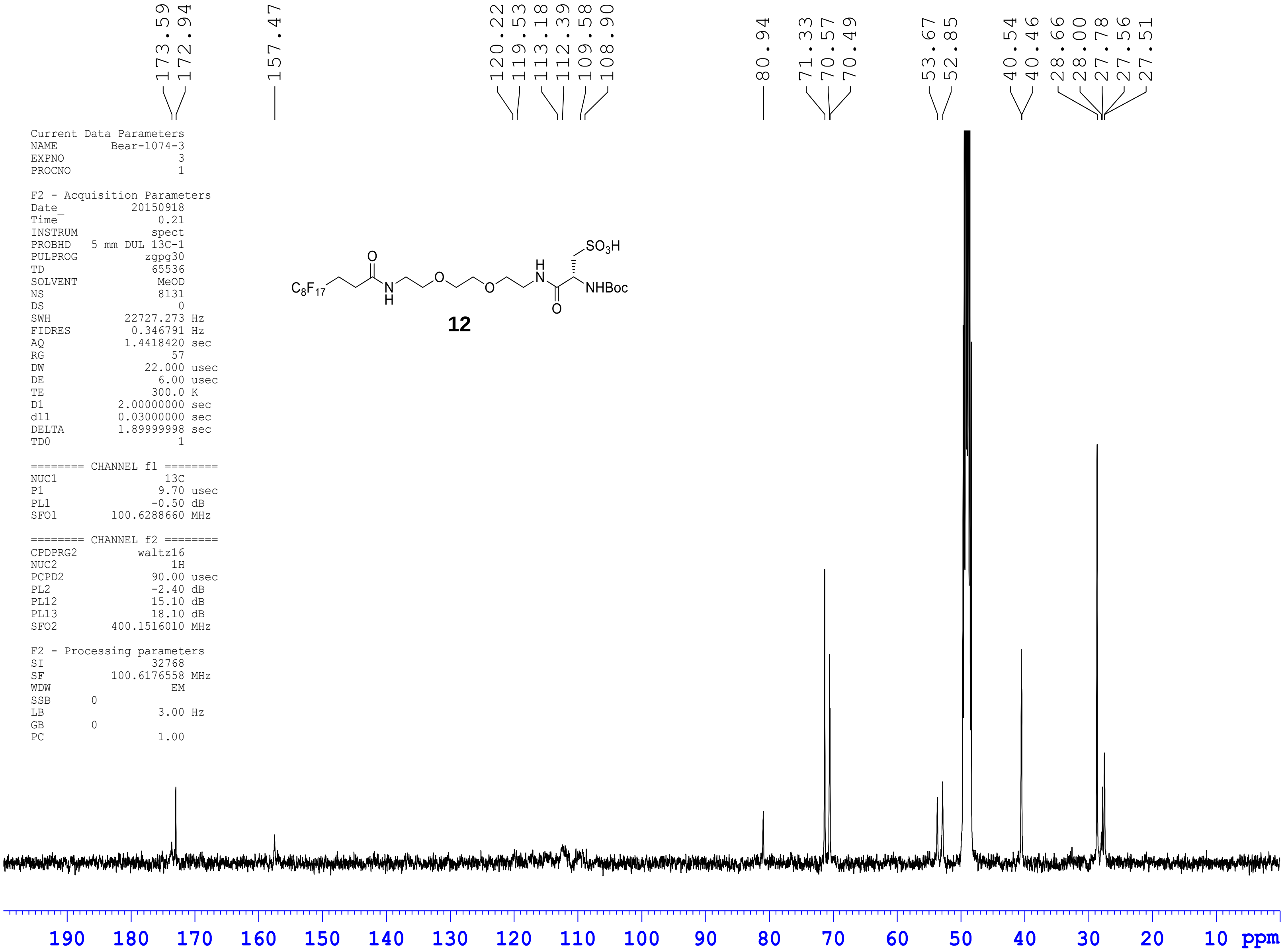
173.59
172.94
157.47
120.22
119.53
113.18
112.39
109.58
108.90
80.94
71.33
70.57
70.49
53.67
52.85
40.54
40.46
28.66
28.00
27.78
27.56
27.51
Current Data Parameters
NAME Bear-1074-3
EXPNO 3
PROCNO 1
F2 - Acquisition Parameters
Date_ 20150918
Time 0.21
INSTRUM spect
PROBHD 5 mm DUL 13C-1
PULPROG zgpg30
TD 65536
SOLVENT MeOD
NS 8131
DS 0
SWH 22727.273 Hz
FIDRES 0.346791 Hz
AQ 1.4418420 sec
RG 57
DW 22.000 usec
DE 6.00 usec
TE 300.0 K
D1 2.00000000 sec
d11 0.03000000 sec
DELTA 1.89999998 sec
TD0 1
======== CHANNEL f1 ========
NUC1 13C
P1 9.70 usec
PL1 -0.50 dB
SFO1 100.6288660 MHz
======== CHANNEL f2 ========
CPDPRG2 waltz16
NUC2 1H
PCPD2 90.00 usec
PL2 -2.40 dB
PL12 15.10 dB
PL13 18.10 dB
SFO2 400.1516010 MHz
F2 - Processing parameters
SI 32768
SF 100.6176558 MHz
WDW EM
SSB 0
LB 3.00 Hz
GB 0
PC 1.00
190 180 170 160 150 140 130 120 110 100 90 80 70 60 50 40 30 20 10 ppm
C8F17
O
N
H
O
O
H
N
SO3H
NHBoc
O

12

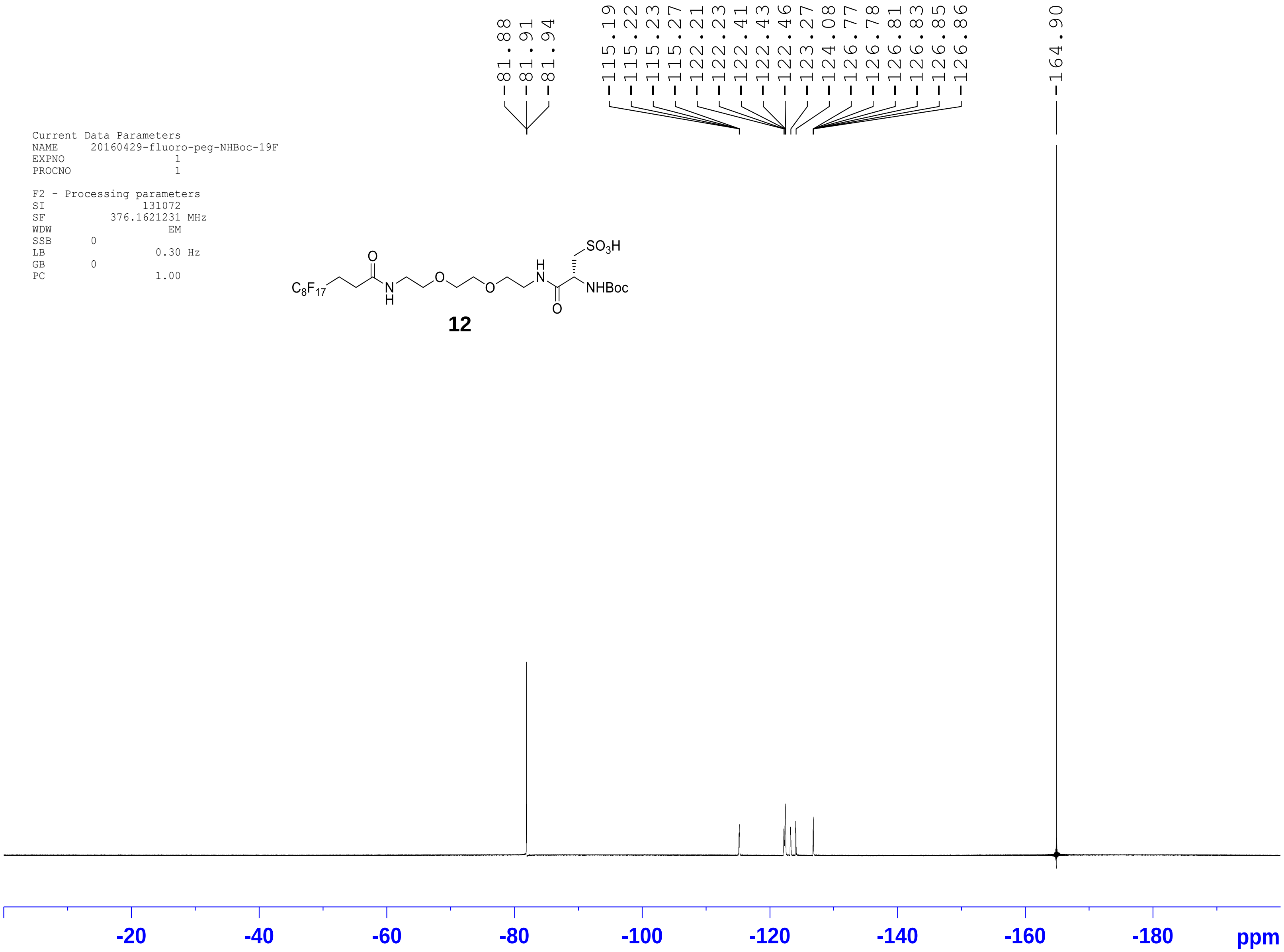

-81.88
-81.91
-81.94
-115.19
-115.22
-115.23
-115.27
-122.21
-122.23
-122.41
-122.43
-122.46
-123.27
-124.08
-126.77
-126.78
-126.81
-126.83
-126.85
-126.86
-164.90
Current Data Parameters
NAME 20160429-fluoro-peg-NHBoc-19F
EXPNO 1
PROCNO 1
F2 - Processing parameters
SI 131072
SF 376.1621231 MHz
WDW EM
SSB 0
LB 0.30 Hz
GB 0
PC 1.00
C8F17
O
N
H
O
O
H
N
SO3H
NHBoc
O
12
-20
-40
-60
-80
-100
-120
-140
-160
-180
ppm

Current Data Parameters
NAME Bear-1139
EXPNO 1
PROCNO 1

F2 - Acquisition Parameters
Date_ 20160510
Time 11.16
INSTRUM spect
PROBHD 5 mm DUL 13C-1
PULPROG zg30
TD 32768
SOLVENT MeOD
NS 15
DS 0
SWH 6410.256 Hz
FIDRES 0.195625 Hz
AQ 2.5559540 sec
RG 128
DW 78.000 usec
DE 6.00 usec
TE 300.0 K
D1 2.00000000 sec
TD0 1

======== CHANNEL f1 ========
NUC1 1H
P1 10.00 usec
PL1 -2.40 dB
SFO1 400.1528010 MHz

F2 - Processing parameters
SI 16384
SF 400.1500068 MHz
WDW EM
SSB 0
LB 0 Hz
GB 0
PC 1.00

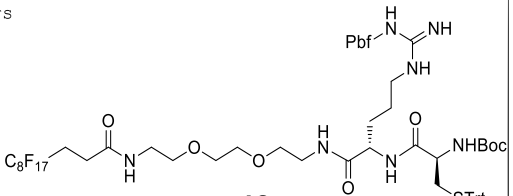

13

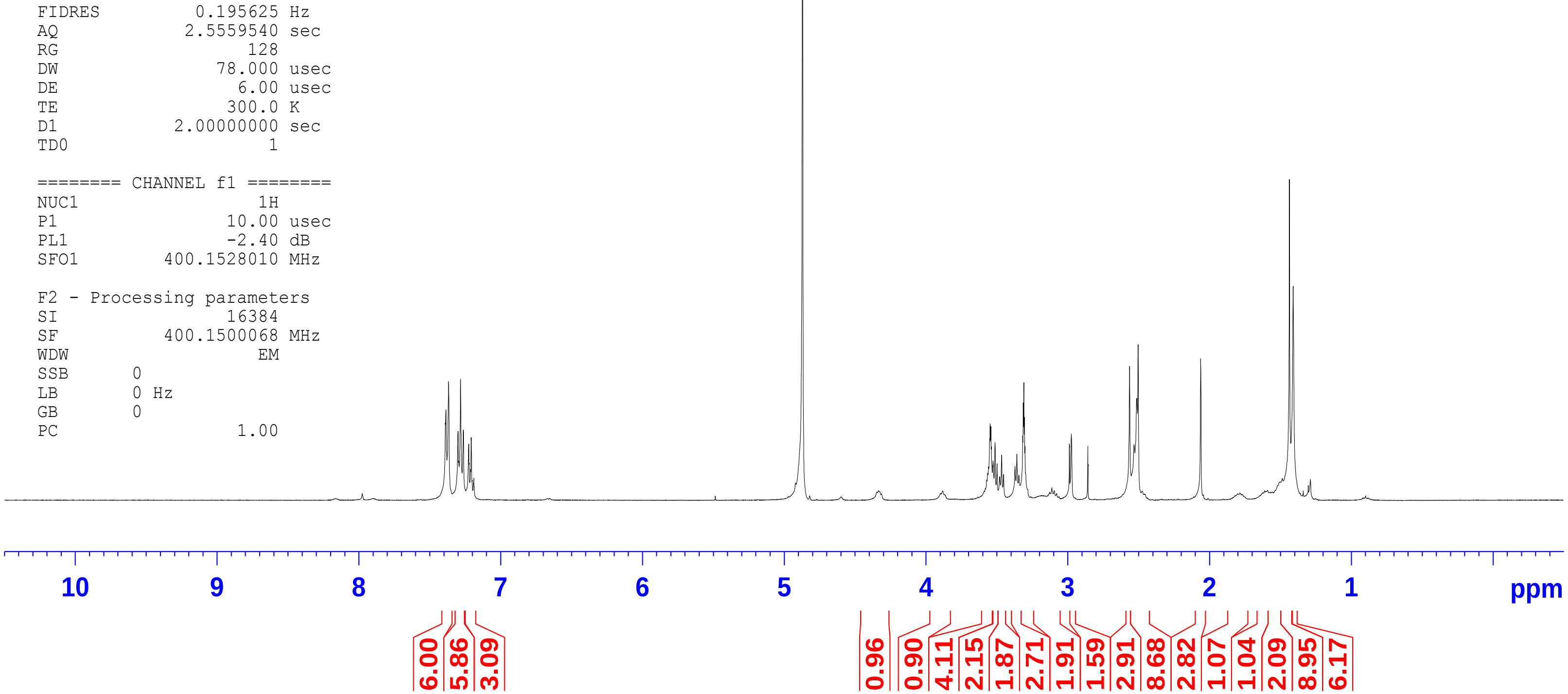

```
Current Data Parameters
NAME      Bear-1139-20160513
EXPNO                    1
PROCNO                   1

F2 - Acquisition Parameters
Date_             20160513
Time                 19.15
INSTRUM              spect
PROBHD    5 mm DUL 13C-1
PULPROG             zgpg30
TD                   65536
SOLVENT               MeOD
NS                     680
DS                       0
SWH              22727.273 Hz
FIDRES            0.346791 Hz
AQ               1.4418420 sec
RG                    50.8
DW                  22.000 usec
DE                    6.00 usec
TE                   300.0 K
D1              2.00000000 sec
d11             0.03000000 sec
DELTA           1.89999998 sec
TD0                      1

======== CHANNEL f1 ========
NUC1                   13C
P1                    9.70 usec
PL1                  -0.50 dB
SFO1           100.6288660 MHz

======== CHANNEL f2 ========
CPDPRG2            waltz16
NUC2                    1H
PCPD2                90.00 usec
PL2                  -2.40 dB
PL12                 15.10 dB
PL13                 18.10 dB
SFO2           400.1516010 MHz

F2 - Processing parameters
SI                   32768
SF             100.6176590 MHz
WDW                     EM
SSB      0
LB                    3.00 Hz
GB       0
PC                    1.00
```

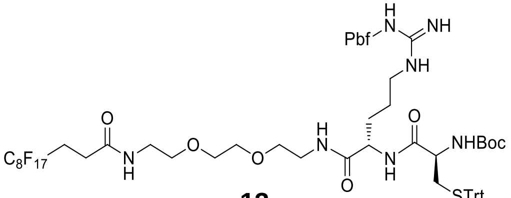

13

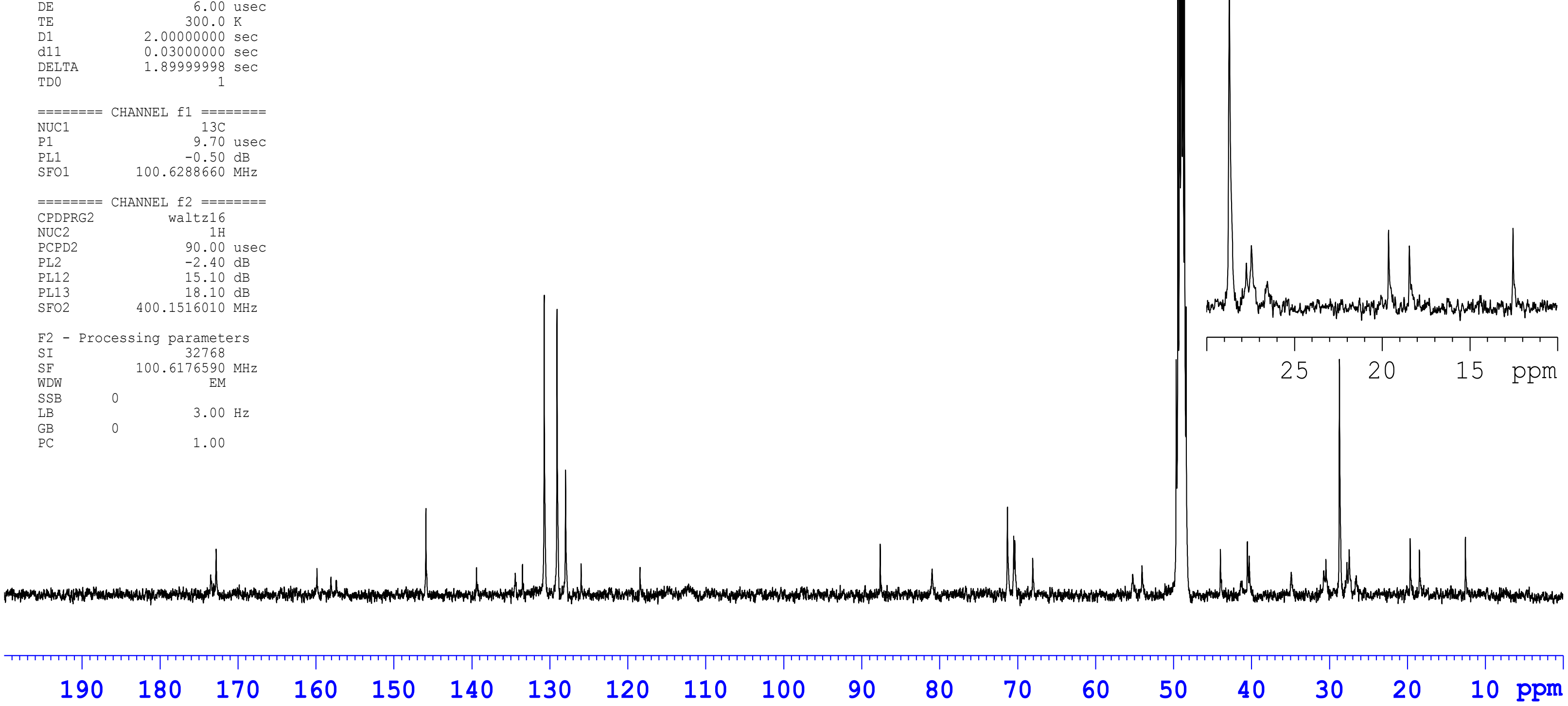

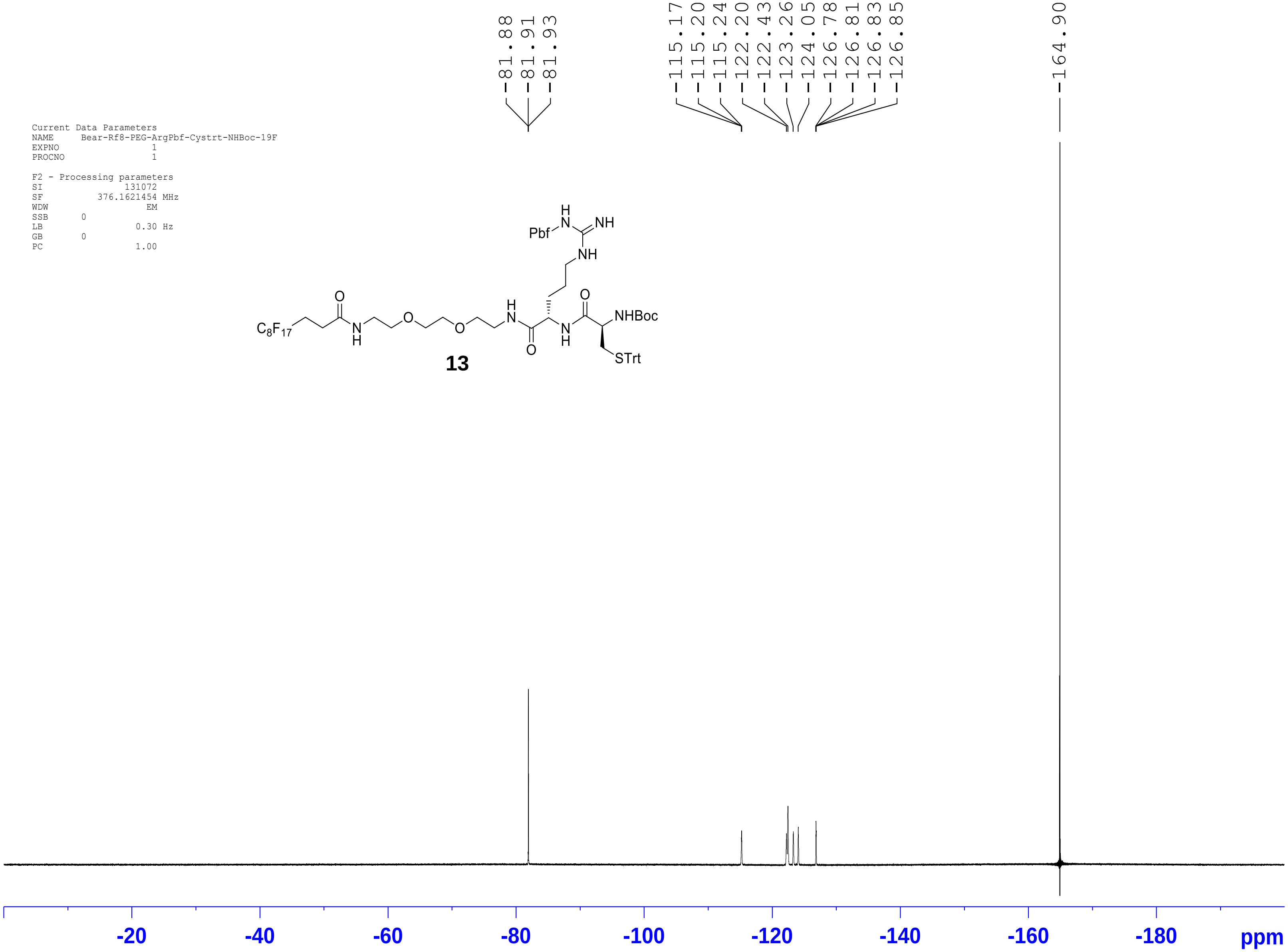
-81.88
-81.91
-81.93
-115.17
-115.20
-115.24
-122.20
-122.43
-123.26
-124.05
-126.78
-126.81
-126.83
-126.85
-164.90
Current Data Parameters
NAME Bear-Rf8-PEG-ArgPbf-Cystrt-NHBoc-19F
EXPNO 1
PROCNO 1
F2 - Processing parameters
SI 131072
SF 376.1621454 MHz
WDW EM
SSB 0
LB 0.30 Hz
GB 0
PC 1.00
Pbf
NH
NHBoc
STrt
C_8F_{17}
13
-20
-40
-60
-80
-100
-120
-140
-160
-180
ppm

```
Current Data Parameters
NAME          Bear-1138-1
EXPNO                   1
PROCNO                  1

F2 - Acquisition Parameters
Date_            20160421
Time                13.42
INSTRUM             spect
PROBHD   5 mm DUL 13C-1
PULPROG              zg30
TD                  32768
SOLVENT               MeOD
NS                     11
DS                      0
SWH              6410.256 Hz
FIDRES           0.195625 Hz
AQ              2.5559540 sec
RG                     57
DW                 78.000 usec
DE                   6.00 usec
TE                  300.0 K
D1             2.00000000 sec
TD0                     1

======== CHANNEL f1 ========
NUC1                   1H
P1                  10.00 usec
PL1                 -2.40 dB
SFO1          400.1528010 MHz

F2 - Processing parameters
SI                  16384
SF            400.1500068 MHz
WDW                    EM
SSB       0
LB        0 Hz
GB        0
PC                   1.00
```

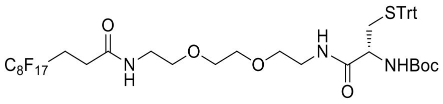

14

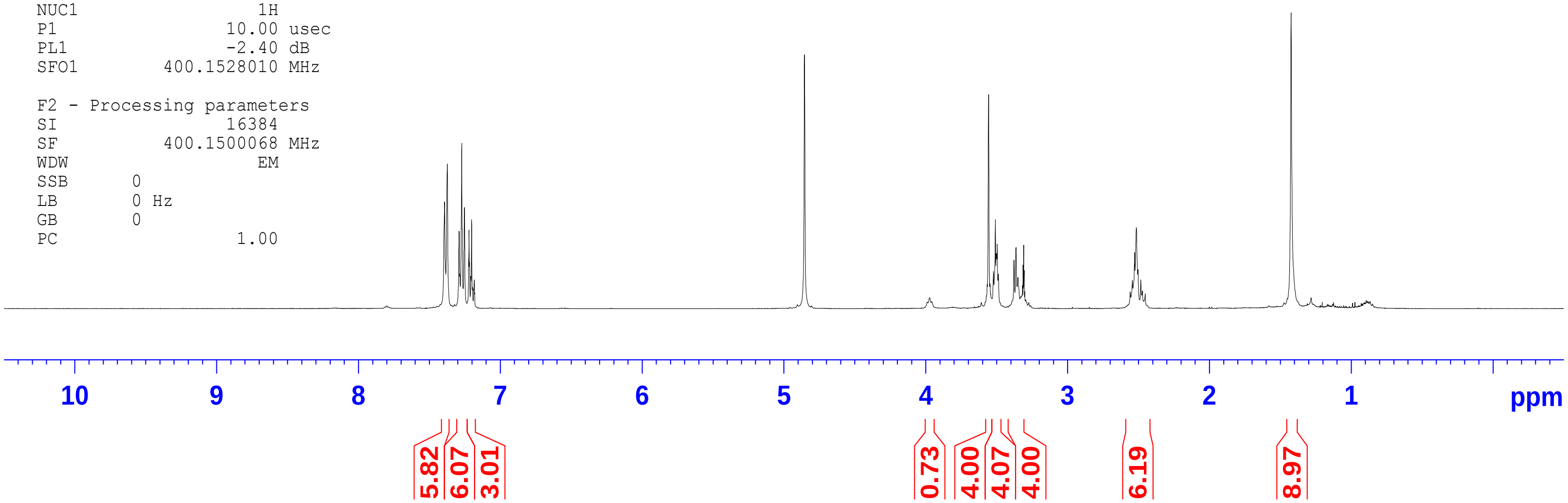

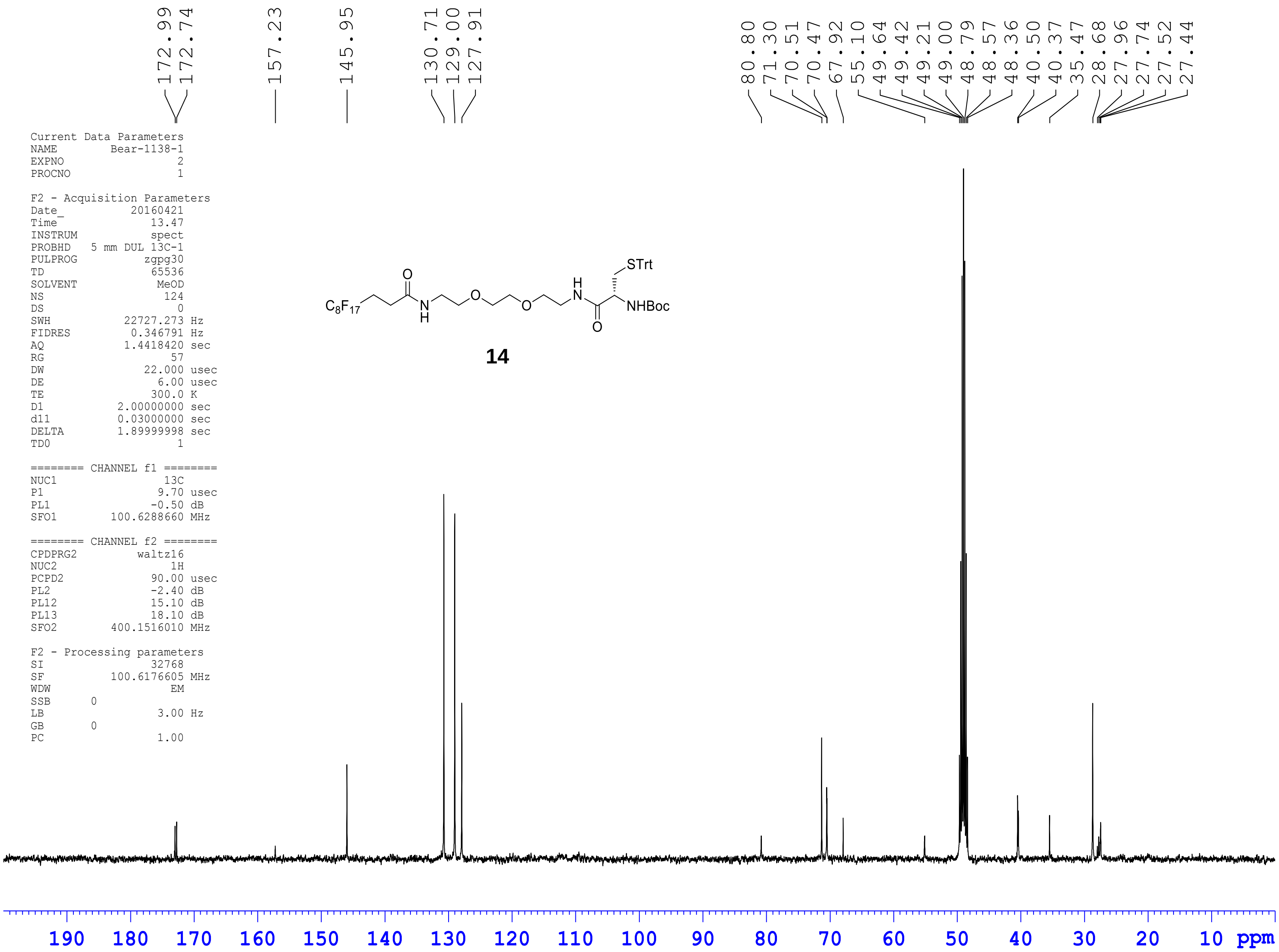
172.99
172.74
157.23
145.95
130.71
129.00
127.91
80.80
71.30
70.51
70.47
67.92
55.10
49.64
49.42
49.21
49.00
48.79
48.57
48.36
40.50
40.37
35.47
28.68
27.96
27.74
27.52
27.44
Current Data Parameters
NAME Bear-1138-1
EXPNO 2
PROCNO 1
F2 - Acquisition Parameters
Date_ 20160421
Time 13.47
INSTRUM spect
PROBHD 5 mm DUL 13C-1
PULPROG zgpg30
TD 65536
SOLVENT MeOD
NS 124
DS 0
SWH 22727.273 Hz
FIDRES 0.346791 Hz
AQ 1.4418420 sec
RG 57
DW 22.000 usec
DE 6.00 usec
TE 300.0 K
D1 2.00000000 sec
d11 0.03000000 sec
DELTA 1.89999998 sec
TD0 1
======== CHANNEL f1 ========
NUC1 13C
P1 9.70 usec
PL1 -0.50 dB
SFO1 100.6288660 MHz
======== CHANNEL f2 ========
CPDPRG2 waltz16
NUC2 1H
PCPD2 90.00 usec
PL2 -2.40 dB
PL12 15.10 dB
PL13 18.10 dB
SFO2 400.1516010 MHz
F2 - Processing parameters
SI 32768
SF 100.6176605 MHz
WDW EM
SSB 0
LB 3.00 Hz
GB 0
PC 1.00
STrt
NHBoc
C_8F_{17}
14
190 180 170 160 150 140 130 120 110 100 90 80 70 60 50 40 30 20 10 ppm

Current Data Parameters
NAME Bear-1138-1-19F
EXPNO 1
PROCNO 1

F2 - Processing parameters
SI 131072
SF 376.1623062 MHz
WDW EM
SSB 0
LB 0.30 Hz
GB 0
PC 1.00

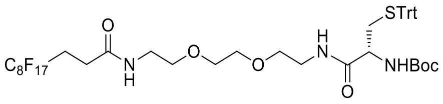

14

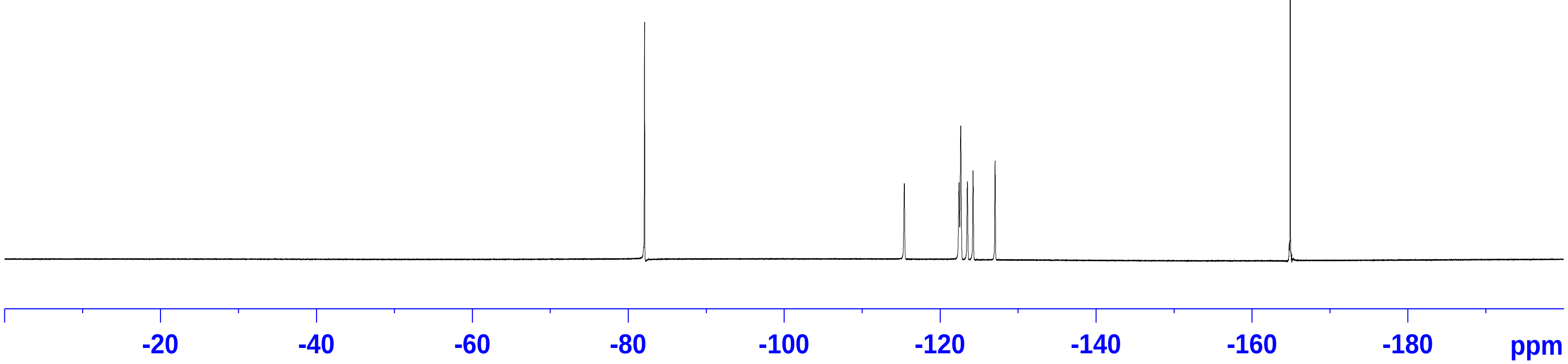

Current Data Parameters
NAME Bear-560
EXPNO 5
PROCNO 1

F2 - Acquisition Parameters
Date_ 20120608
Time 13.57
INSTRUM spect
PROBHD 5 mm DUL 13C-1
PULPROG zg30
TD 32768
SOLVENT MeOD
NS 9
DS 0
SWH 6410.256 Hz
FIDRES 0.195625 Hz
AQ 2.5559540 sec
RG 4
DW 78.000 usec
DE 6.00 usec
TE 300.0 K
D1 2.00000000 sec
TD0 1

======== CHANNEL f1 ========
NUC1 1H
P1 10.00 usec
PL1 -2.40 dB
SFO1 400.1528010 MHz

F2 - Processing parameters
SI 16384
SF 400.1500064 MHz
WDW EM
SSB 0
LB 0 Hz
GB 0
PC 1.00

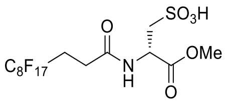

15

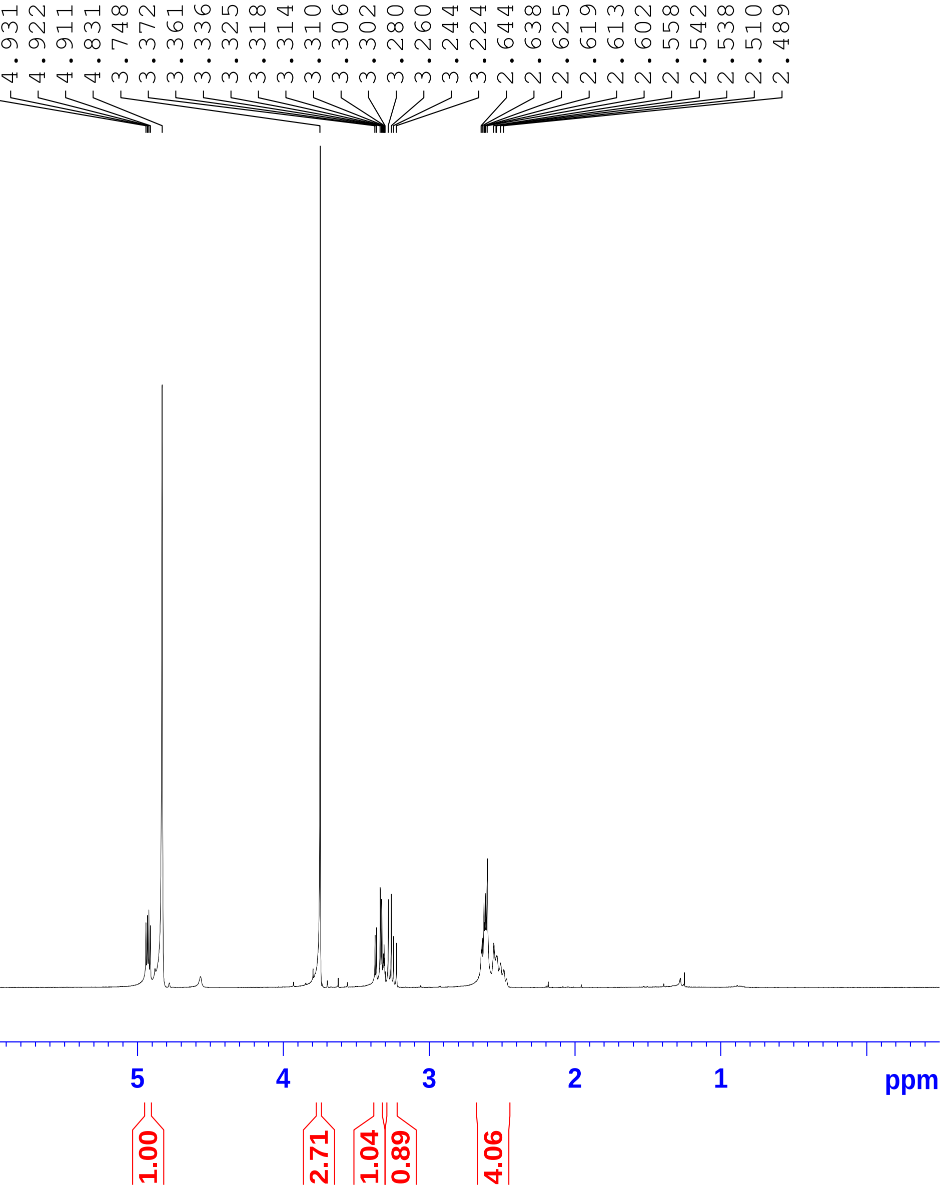

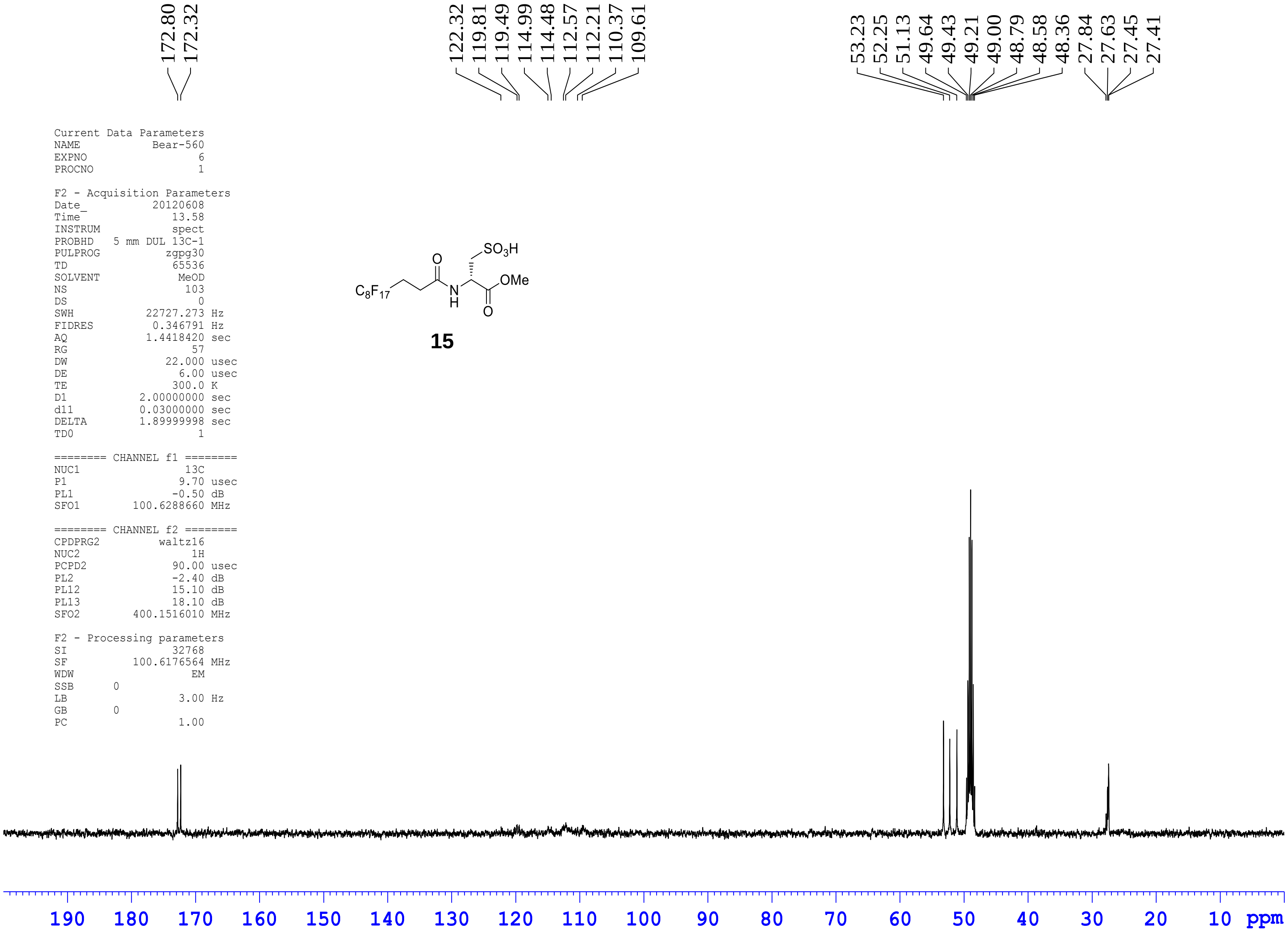
172.80
172.32
122.32
119.81
119.49
114.99
114.48
112.57
112.21
110.37
109.61
53.23
52.25
51.13
49.64
49.43
49.21
49.00
48.79
48.58
48.36
27.84
27.63
27.45
27.41
Current Data Parameters
NAME Bear-560
EXPNO 6
PROCNO 1
F2 - Acquisition Parameters
Date_ 20120608
Time 13.58
INSTRUM spect
PROBHD 5 mm DUL 13C-1
PULPROG zgpg30
TD 65536
SOLVENT MeOD
NS 103
DS 0
SWH 22727.273 Hz
FIDRES 0.346791 Hz
AQ 1.4418420 sec
RG 57
DW 22.000 usec
DE 6.00 usec
TE 300.0 K
D1 2.00000000 sec
d11 0.03000000 sec
DELTA 1.89999998 sec
TD0 1
======== CHANNEL f1 ========
NUC1 13C
P1 9.70 usec
PL1 -0.50 dB
SFO1 100.6288660 MHz
======== CHANNEL f2 ========
CPDPRG2 waltz16
NUC2 1H
PCPD2 90.00 usec
PL2 -2.40 dB
PL12 15.10 dB
PL13 18.10 dB
SFO2 400.1516010 MHz
F2 - Processing parameters
SI 32768
SF 100.6176564 MHz
WDW EM
SSB 0
LB 3.00 Hz
GB 0
PC 1.00
C8F17
O
N
H
SO3H
OMe
O
15
190 180 170 160 150 140 130 120 110 100 90 80 70 60 50 40 30 20 10 ppm

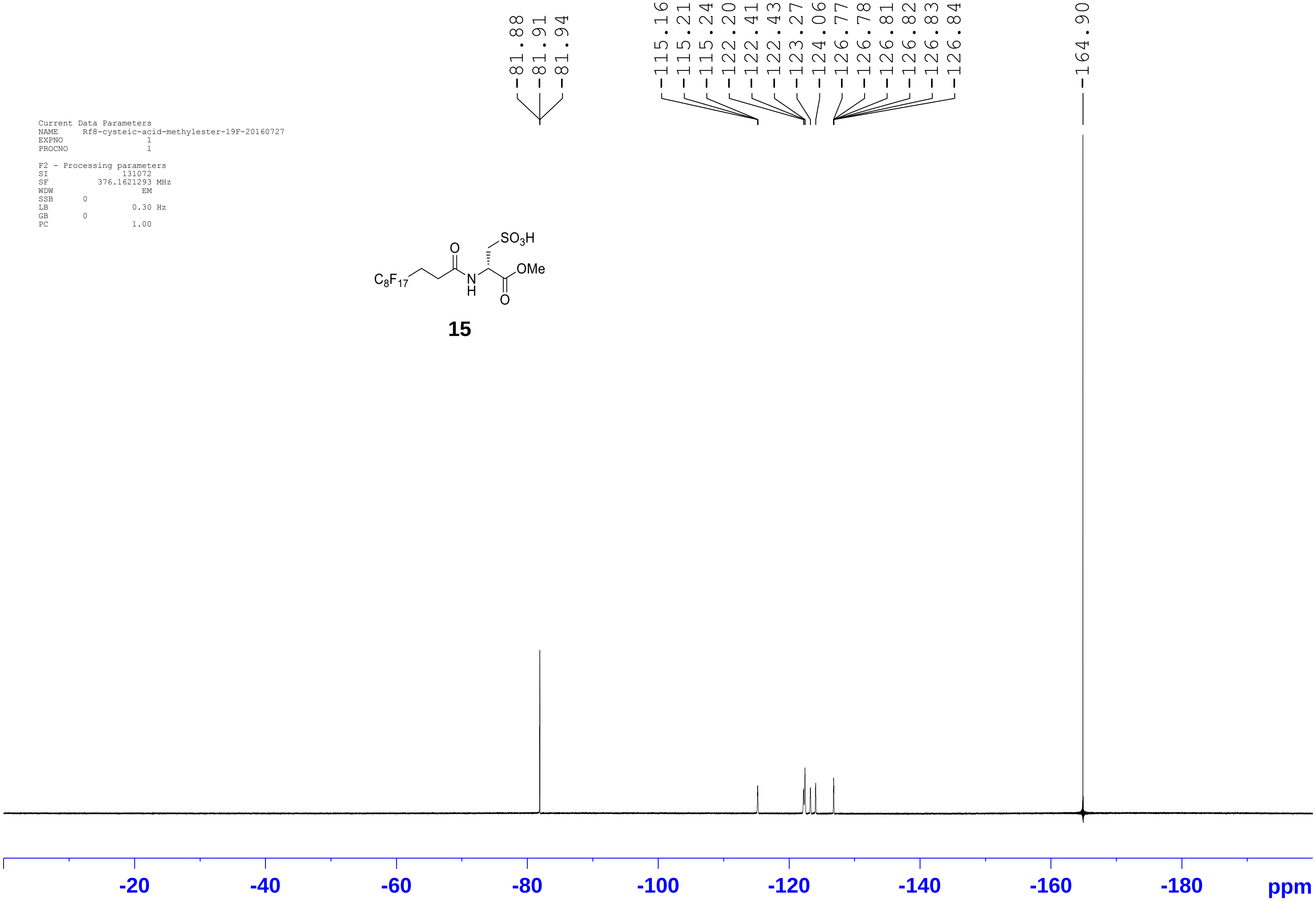
Current Data Parameters
NAME Rf8-cysteic-acid-methylester-19F-20160727
EXPNO 1
PROCNO 1
F2 - Processing parameters
SI 131072
SF 376.1621293 MHz
WDW EM
SSB 0
LB 0.30 Hz
GB 0
PC 1.00
-81.88
-81.91
-81.94
-115.16
-115.21
-115.24
-122.20
-122.41
-122.43
-123.27
-124.06
-126.77
-126.78
-126.81
-126.82
-126.83
-126.84
-164.90
SO3H
O
OMe
C8F17
N
H
O
-20
-40
-60
-80
-100
-120
-140
-160
-180
ppm

15
